# A New Susceptibility Locus for Myocardial Infarction, Hypertension, Type 2 Diabetes Mellitus, and Dyslipidemia on Chromosome 12q24

**DOI:** 10.1155/2014/291419

**Published:** 2014-06-26

**Authors:** Salma M. Wakil, Nzioka P. Muiya, Asma I. Tahir, Mohammed Al-Najai, Batoul Baz, Editha Andres, Nejat Mazhar, Nada Al Tassan, Maie Alshahid, Brian F. Meyer, Nduna Dzimiri

**Affiliations:** ^1^Genetics Department, King Faisal Specialist Hospital and Research Centre, Riyadh 11211, Saudi Arabia; ^2^King Faisal Heart Institute, King Faisal Specialist Hospital and Research Centre, Riyadh 11211, Saudi Arabia; ^3^Cardiovascular and Pharmacogenomics Unit, MBC-03-05, Genetics Department, King Faisal Specialist Hospital and Research Centre, P.O. Box 3354, Riyadh 11211, Saudi Arabia

## Abstract

We examined the role of hepatic nuclear factor-1 alpha (*HNF1a*) gene polymorphism on coronary artery disease (CAD) traits in 4631 Saudi angiographed individuals (2419 CAD versus 2212 controls) using TaqMan assay on ABI Prism 7900HT sequence detection system. Following adjustment for confounders, the rs2259820_CC (1.19 (1.01–1.42); *P* = 0.041), rs2464196_TT (1.19 (1.00–1.40); *P* = 0.045), and rs2259816_T (1.13 (1.01–1.26); *P* = 0.031) were associated with MI. The rs2259820_T (1.14 (1.03–1.26); *P* = 0.011) and rs2464196_C (1.12 (1.02–1.24); *P* = 0.024) were associated with type 2 diabetes mellitus (T2DM), while the rs2393791_T (1.14 (1.01–1.28); *P* = 0.032), rs7310409_G (1.16 (1.03–1.30); *P* = 0.013), and rs2464196_AG+GG (1.25 (1.05–1.49); *P* = 0.012) were implicated in hypertension. Hypertriglyceridemia was linked to the rs2393791_T (1.14 (1.02–1.27); *P* = 0.018), rs7310409_G (1.12 (1.01–1.25); *P* = 0.031), rs1169310_G (1.15 (1.04–1.28); *P* = 0.010), and rs1169313_CT+TT (1.24 (1.06–1.45); *P* = 0.008) and high low density lipoprotein-cholesterol levels were associated with rs2259820_T (1.23 (1.07–1.41); *P* = 0.004), rs2464196_T (1.22 (1.06–1.39); *P* = 0.004), and rs2259816_T (1.18 (1.02–1.36); *P* = 0.023). A 7-mer haplotype CATATAC (*χ* ^2^ = 7.50; *P* = 0.0062), constructed from the studied SNPs, was associated with MI, and CATATA implicated in T2DM (*χ* ^2^ = 3.94; *P* = 0.047). Hypertriglyceridemia was linked to TGCGGG (*χ* ^2^ = 4.26; *P* = 0.039), and obesity to ACGGGT (*χ* ^2^ = 5.04; *P* = 0.025). Our results suggest that the *HNF1a* is a common susceptibility gene for MI, T2DM, hypertension, and dyslipidemia.

## 1. Background

**Figure 1:**
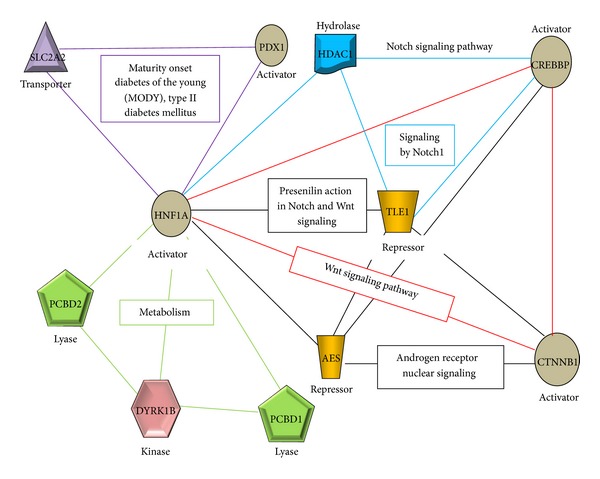
Schematic diagram representation of some of the pathways regulated by HNF1a. AES: androgen receptor nuclear signaling, Wnt signalling pathway. CREBBP: Notch signaling pathways, signaling by Notch1. CTNNB1: androgen receptor nuclear signaling, Wnt signalling pathway. DYRK1B: glucose/energy metabolism. HDAC1: Notch signaling pathways, signaling by Notch1. HNF1a: maturity onset diabetes of the young (MODY), type II diabetes mellitus, presenilin action in Notch and Wnt signaling, and metabolism. PCBD1: metabolism. PCBD2: metabolism. PDX1: maturity onset diabetes of the young, type II diabetes mellitus. SLC2A2: maturity onset diabetes of the young, type II diabetes mellitus. TLE1: Wnt signaling pathway, signaling by Notch1, and presenilin action in Notch and Wnt signaling.

The hepatocyte nuclear factor 1a gene (*HNF1a*, also known as transcription factor-1) on chromosome 12q24 encodes a transcription factor (TF) that binds to promoters of a variety of genes expressed predominantly in the liver [1, 2]. Different sections of the* HNF1a* gene are transcribed to specific HNF1a isoforms, which might serve different yet unidentified functions [3–6]. This TF regulates several target genes involved in lipoprotein metabolism, including apolipoproteins, cholesterol synthesizing enzymes, and bile acid transporters, as well as glucose-stimulated insulin secretion [7, 8] (Figure 1). Hence, alterations in the encoding gene are likely to lead to disorders in the various metabolic pathways associated with liver function. Currently, mutations in the* HNF1a* gene are known to be the most common cause of maturity onset diabetes of the young (MODY), a severe dominantly inherited form of nonketotic diabetes mellitus that is characterized by pancreatic beta-cell dysfunction [9–15]. This disease is a result of a primary defect in insulin secretion and usually develops at childhood, adolescence, or young adulthood. However, while MODY is predominantly inherited, its penetrance and expression may vary in presence or absence of a family history of diabetes [16]. Moreover, there appears to be a substantial heterogeneity in the etiology of the disease, even if the source of the disease may be the same [16–18]. Besides,* HNF1a* mutations have also been implicated in diabetic nephropathy related to type 1 diabetes [19] and non-insulin-dependent, type 2 diabetes mellitus (T2DM) [20–23], albeit not as pronounced as in MODY disorders [24–26].

Apart from diabetic disorders, single nucleotide polymorphisms (SNPs) in the* HNF1a* have also been linked to changes in plasma concentrations of the C-reactive protein (CRP) [27–35], arguably a powerful risk marker for cardiovascular disease. The gene has similarly been implicated in coronary artery disease (CAD)/myocardial infarction (MI) [30, 36] as well as its risk traits, including dyslipidemic disorders [30, 37], whereby an increased risk for CAD was also observed in T2DM patients harbouring* HNF1a* mutations [34]. However, these findings have either not been replicable in other studies or partly refuted by other investigators or meta-analysis studies [38–41]. Hence, the role of the* HNF1A* in the onset and development of CAD is yet to be clearly defined. Besides, the fact that* HNF1A* polymorphism has been linked with susceptibility to both CAD and its important risk traits points to some pleiotropic actions of the gene on disease pathways leading to atherosclerosis. In a preliminary linkage study in a heterozygous familial hypercholesterolemia setting, we established a link for early onset CAD to the locus of the* HNF1a* gene on chromosome 12q24 and suggested a role for this gene in this disease process. In the present study, therefore, we elected to test this notion further and to evaluate the potential role of the gene variants at this locus in CAD risk traits and its manifestation in a large cohort of angiographed Saudi individuals.

## 2. Materials and Methods

### 2.1. Study Population

The initial linkage study was performed in a family of eleven with heterozygous familial hypercholesterolemia (see* HNF1a* Supplementary Data 1 in the Supplementary Material available online at http://dx.doi.org/10.1155/2014/291419), in which the primary proband underwent triple bypass surgery at the age of 14. Sequencing the* HNF1a* gene for variants of interest was accomplished in the family members as well as 200 other individuals from the general population. This was followed by a case-control study in a total of 4631 candidates consisting of 2419 CAD patients (1852 males and 567 females, mean age 59.7 ± 0.2 yr) with angiographically determined narrowing of the coronary vessels by at least 50% and 2212 angiographed controls (1183 male and 1029 female, mean age 47.1 ± 0.4 yr) with no evidence of the CAD disease (Table 1(a)). Among these 4631 individuals, 3044 candidates had established MI, 3528 had primary (essential) hypertension (HTN), and 2550 had T2DM (formerly known as non-insulin-dependent diabetes mellitus or adult onset diabetes) (Table 1(b)). The National Diabetes Data Group of the USA and the second World Health Organization Expert Committee on Diabetes Mellitus (1998) [42] define T2DM as a metabolic disorder that is characterized by high blood glucose (generally defined as fasting glucose level >126 mg/dL) in the context of insulin resistance and relative insulin deficiency. Primary hypertension was defined as ≥140 mm Hg systolic blood pressure or ≥90 mm Hg diastolic pressure based on the Sixth Report of the Joint National Committee on Prevention, Detection, Evaluation, and Treatment of High Blood Pressure (JNC VI) criteria [43]. Accordingly, essential, primary, or idiopathic hypertension is defined as high blood pressure (HBP) in which secondary causes such as renovascular disease, renal failure, pheochromocytoma, aldosteronism, or other causes of secondary hypertension or Mendelian forms (monogenic) are not present [43]. The fourth group comprised 1759 obese candidates with body mass index of ≥30.0 kg/m^2^ and is classified as the obesity (OBS) subset. Among these subsets of patients, some individuals harboured a combination of two or possibly three of the cardiovascular risk traits. The overall exclusion criteria for the disease cases were major cardiac rhythm disturbances, incapacitating or life-threatening illness, major psychiatric illness or substance abuse, history of cerebral vascular disease, neurological disorder, and administration of psychotropic medication. The controls (CON) for CAD were a group of individuals undergoing surgery for heart valvular diseases and those who may have reported with chest pain but were established to have no significant coronary stenosis by angiography. Exclusion criteria for this group were among others diseases such as cancer, autoimmune disease, or any other disorders likely to interfere with variables under investigation. This study was performed in accordance with the regulations laid down by the King Faisal Specialist Hospital and Research Centre Ethics Committee in compliance with the Helsinki Declaration (http://www.wma.net/en/30publications/10policies/b3/index.html) and all participants signed an informed consent.

### 2.2. Linkage Analysis and Screening for Mutations

Five mL of peripheral blood was sampled in EDTA tubes from each of the study individuals after obtaining their written consent, and genomic DNA extracted from leukocytes by the standard salt method using Puregene DNA isolation kit (Gentra system, Minneapolis, MN, USA). For the whole genome-wide scanning with the Affymetrix Gene Chip 250 Sty1 mapping array (Affymetrix Inc., Santa Clara, CA, USA), 250 ng of genomic DNA was digested with the restriction endonuclease StyI, mixed with Sty1 adaptors, and ligated with T4 DNA ligase. The mixture was added to four separate PCR reactions, amplified, pooled, and purified to remove the unincorporated dNTPs. The PCR product was then fragmented, biotinylated, hybridized to the 250 Sty1 array for 18 h, washed, stained, and scanned as recommended by the manufacturer. SNP genotypes, linear chromosomal locations, and marker ordering were accomplished using the Affymetrix GeneChip Genotyping Analysis Software (GTYPE) version 4.0. Multipoint parametric linkage analysis was performed using the GeneHunter Easy Linkage analysis software 4.0 (Affymetrix, Inc., Santa Clara, CA, USA) for estimating the LOD scores. Genotype calling rates for the samples ranged from 98.23% to 99.83%, which was high and accurate enough for their subsequent evaluation. The disease was assumed to be an autosomal dominant trait with 100% penetrance. Copy Number Analyzer for GeneChip Ver. 3.0 (CNAG) (Affymetrix, Inc., Santa Clara, CA, USA) was employed to establish the shared chromosomal regions of homozygosity.

### 2.3. Sequencing of the* HFN1a* Gene

The linkage study was followed by sequencing exons and intron-exon junctions and flanking regions of the* HFN1A *gene in the family members and 200 individuals from the general population using the MegaBACE DNA analysis system (Amersham Biosciences, Sunnyvale, CA, USA). Briefly, the DNA was subjected to PCR by standard methods described elsewhere. Five *μ*L of PCR product was then treated with 2 *μ*L of ExoSAP-IT (USB Corporation, Cleveland, Ohio, USA) at 37°C for 30 min to allow the hydrolytic removal of excess primers and dNTPs by Exonuclease 1 and shrimp alkaline phosphatase. The enzymes were inactivated at 80°C for 15 min, and the sequencing reaction was initiated by mixing 2 *μ*L DNA, 1 *μ*L of 5 *μ*mol primer, 8 *μ*L of DYEnamic ET Dye Terminator (Amersham Biosciences, Buckinghamshire, UK), and 9 *μ*L of distilled water. The mixture was thermally cycled 40x at 95°C for 20 sec, 50°C for 15 sec, and 60°C for 1 min. Unincorporated dye-labelled terminators were removed by gel-filtration through the DyeEx 96 plate (Qiagen, GmbH, Hilden, Germany). The eluent was vacuum-dried and dissolved in 10 *μ*L of loading solution (GE Healthcare UK Ltd, Little Chalfont, Buckinghamshire UK) for sequencing. Data were analyzed for SNPs by Lasergene software (DNASTAR, Inc., Madison, WI, USA).

### 2.4. Association Experiments

Once the SNPs of interest were identified, genotyping was achieved by TaqMan chemistry using the Applied Biosystems real-time Prism 7900HT Sequence Detection System (ABI Inc., CA, USA). Primers and the TaqMan fluorogenic probes bearing a suitable reporter dye on the 5′-end and a quencher dye on the 3′-end were designed using the Primer Express software V2.0 (ABI Inc., Foster City, CA, USA) and procured from Applied Biosystems (ABI, Warrington, UK). One probe (for allele 1) was labeled with VIC dye and the other (for allele 2) with FAM dye at the 5′-end, and serial dilutions were run to determine the optimal working concentration. For each reaction, a 25 *μ*L reaction was prepared by mixing 5 *μ*L containing 50 ng DNA, 12.5 *μ*L of 2x Universal mix (Eurogentec, Liege Science Park, Seraing, Belgium), 1.25 *μ*L of 20x probe assay mix, and 6.25 *μ*L DNase-free distilled water. Three no-template controls were included in each plate for normalization of emission signal. The thermal amplification profile for the first cycle occurred at 50°C for 2 min and 95°C for 10 min followed by 40 cycles of 94°C for 15 sec and 60°C for 30 sec. The plates were then scanned for FRET signal using the 7900HT sequence detection system and data analyzed using SDS 2.0 software (ABI, Foster City, CA, USA).

## 3. Statistical Analysis

Mann-Whitney* U* (MWU) test, Bonferroni adjustment, and binary logistic regression analyses were employed to compute odds ratios and their 95% confidence intervals as well as estimate the confounding effects of the different cardiovascular risk traits on the respective relationships. For the haplotype-based association analysis, we used the haplo.stats package (http://mayoresearch.mayo.edu/mayo/research/schaid_lab/software.cfm) in the R Statistical Computing Software (http://www.r-project.org/). Odd ratios for haplotypes were calculated using the baseline haplotype CATATAC (frequency = 0.505) as reference, and the haplotype score statistic for the association of a haplotype with the binary trait was calculated as in Schaid et al. (2002) [44] and Lake et al. (2003) [45]. Significance of association was determined between haplotypes and the case-control status—a binomial trait denoting whether or not a patient had the disease. All other statistical analyses were performed using the SPSS software version 20 (SPSS Inc., Chicago, USA), and data are expressed as mean ± SEM. Associations with a two-tailed* P *value <0.05 were considered statistically significant.

## 4. Results

**Figure 2:**
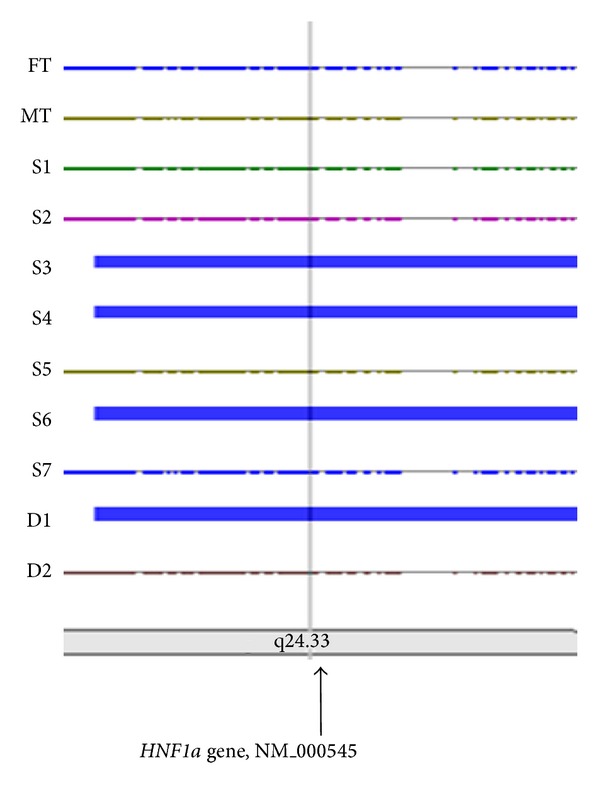
Homozygosity mapping for early onset of coronary artery disease in heterozygous familial hypercholesterolemia.

**Figure 3:**
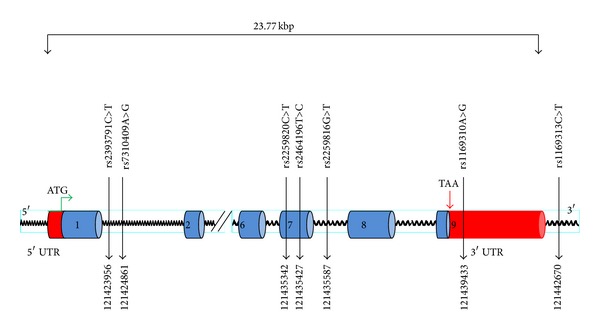
Schematic presentation of the studied gene variants.

The results of whole genome scan experiments using the Affymetrix Gene Chip 250 Sty1 mapping array pointed to a number of genomic loci as potential risk for HFH and early onset of CAD in a study involving a family of 11 members harbouring HFH. These loci included that of the* HNF1a* gene on chromosome 12q24 (Figure 2). The ensuring sequencing of the gene in the family members and 200 other individuals from the general population revealed several informative SNPs (with frequencies of >0.1) that were of potential interest, from which seven were selected for the association studies in a population of 4631 candidates. These SNPs included (1) rs2393791_C>T, (2) rs7310409_A>G, (3) rs2259820_C>T (p.Leu459Leu), (4) rs2464196_T>C, (p.Ser487Asn), (5) rs2259816_G>T, (6) rs1169310_A>G, and (7) rs1169313_C>T numbered sequentially by their chromosomal positions (Figure 3).

We first performed the MWU test on the data, which demonstrated significant associations of the various SNPs with different disease traits (*HNF1a* Supplementary Data 2). We then subjected the data to binary logistical regression analysis for the respective conditions, whereby the coexisting disease traits and other covariates were treated as confounders, and Bonferroni adjustment for age and sex was performed on the data (*HNF1a* Supplementary Data 3). To begin with, the initial univariate analysis suggested that six of these variants rs7310409 A>G (*P* = 0.041), rs2259820_C>T (*P* = 0.011), rs2464196_C>T (*P* = 0.016), rs2259816_G>T (0.007), rs1169310_A>G (*P* = 0.016), and rs1169313_C>T (*P* = 0.023) conferred risk for MI (3044 cases versus 1587 controls). However, following adjustment for possible confounding effects of all other risk covariates, only the rs2259820_CC (odds ratio (95% confidence interval) = 1.19 (1.01–1.41); *P* = 0.013), rs2464196_TT (1.19 (1.00–1.40); *P* = 0.042), and rs2259816_T (1.13 (1.01–1.26); *P* = 0.031) retained their significant association with the disease, while the relationships for the other three variants turned weaker or diminished (Table 2). Notably, the tests for the association of these variants with the classical CAD/MI risk traits also implicated the rs2259820_T (1.14 (1.03–1.26); *P* = 0.011) and rs2464196_C (1.12 (1.02–1.24); *P* = 0.024) further in T2DM (2550 versus 2081), as well as the rs2393791_T (1.14 (1.01–1.28); *P* = 0.032), rs7310409_G (1.16 (1.03–1.30); *P* = 0.013), and rs2464196_AG+GG (1.25 (1.05–1.49); *P* = 0.012) in HTN (3528 versus 1103), following adjustment for possible confounding effects of coexisting disease traits and other covariates (*HNF1a* Supplementary Data 4). Interestingly, dyslipidemia was linked to all the variants, with four of them the rs2393791_T (1.14 (1.02–1.27); *P* = 0.018), rs7310409 (1.12 (1.01–1.25); *P* = 0.031), rs1169310_G (1.15 (1.04–1.28); *P* = 0.010), and rs1169313_CT+TT (1.24 (1.06–1.45); *P* = 0.008) being implicated in hypertriglyceridemia (hTG; 1160 versus 3156) as well as rs2259820_T (1.23 (1.07–1.41); *P* = 0.004), rs2464196_T (1.22 (1.06–1.39); *P* = 0.004), and rs2259816_T (1.18 (1.02–1.36); *P* = 0.023) conferring a risk for harbouring high low-density lipoprotein-cholesterol (hLDLC) levels. None of the studied SNPs was associated with gender or history of CAD.

**Figure 4:**
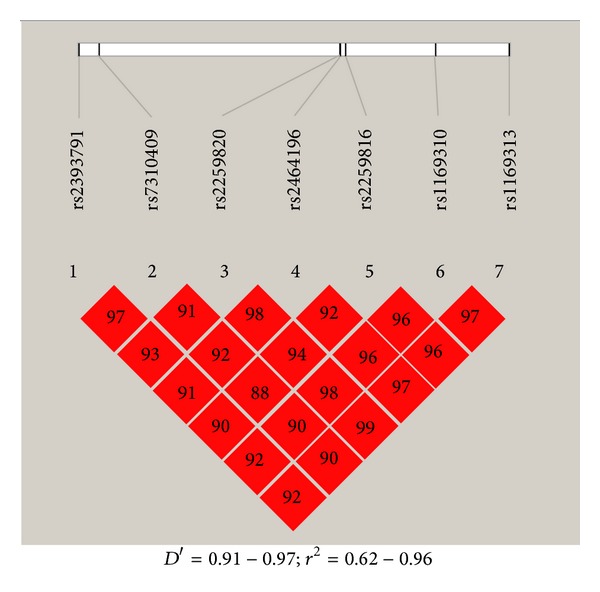
The linkage disequilibrium plot for the studied SNPs.

We further evaluated the possible impact of the haplotypes at this locus on the various disease traits. The linkage disequilibrium plot for the studied SNPs is given in Figure 4. We used the most common 7-mer haplotype CATATAC (frequency = 0.505) constructed from the SNPs as a baseline for comparing their relationships with the disease traits (Table 3). Our results revealed that the baseline 7-mer haplotype CATATAC (*χ* ^2^ = 7.50; *P* = 0.0062) conferred a risk for MI (*HNF1a* Supplementary Data 5). Notably, its 6-mer (1–6) CATATA (*χ* ^2^ = 7.48; *P* = 0.0062) and 5-mer (1–5) CATAT (*χ* ^2^ = 7.68; *P* = 0.0058) derivatives were equally implicated in the disease. Further analysis pointed to the G>T change at this locus as explaining the difference between being causative and protective, as demonstrated by the fact that the 7-mer CATAGAC (*χ* ^2^ = 6.06; *P* = 0.014) was equally protective. Interestingly, the haplotype CATATAC (*χ* ^2^ = 3.94; *P* = 0.047) and its 4-mer derivative CATA was also implicated, albeit less significantly so in T2DM, pointing to MI and T2DM sharing common causative genomic sequences at this locus. No haplotype was positively associated with either hypercholesterolemia (hChol) or the harbouring low levels of high density lipoprotein-cholesterol (lHDLC). However, hypertriglyceridemia (hTG) was linked to TGCGGG (*χ* ^2^ = 4.26; *P* = 0.039) and its 5-mer TGCGG (*χ* ^2^ = 4.61; *P* = 0.032) derivative, while obesity was associated with ACGGGT (*χ* ^2^ = 5.04; *P* = 0.025) and its 5-mer derivative ACGGG (*χ* ^2^ = 5.84; *P* = 0.016).

## 5. Discussion

The present study evaluated the relationships of gene variants on chromosomal 12q24 with CAD/MI and its various risk traits. We described the association for at least three of the studied SNPs, rs2259820, rs2464196, and rs2259816, and a weak link for two others, with the risk for MI. These variants reside in the chromosomal region that harbours the* HNF1a* gene, possibly pointing directly to this gene as the potential culprit. Currently, there appears to be stealth of information on the role of the* HNF1a* gene or its genomic locus in CAD/MI, in general. The available literature is somewhat conflicting, with some investigators implicating this locus in the disease [30, 36] and others failing to establish such a relationship [38–41]. For example, although five of the variants included in the present study, rs2293791, rs7310409, rs2464196, rs2259816, and rs1169310, have been recently linked with changes in CRP [28, 32–34], a marker for cardiovascular disease, their direct involvement in CAD manifestation remains disputable. Thus, while some studies have implicated variants, such as the rs7310409 in acute coronary syndrome [40], rs2259816 in CAD [34], and rs2464196 in subclinical coronary atherosclerosis [30], others reported only a weak or no link at all for rs2259816 with CAD in the presence of a decrease in C-reactive protein levels [28]. The observation of associations for these variants with MI in the present study clearly indicates that further studies are warranted to ascertain globally the role of this genomic locus in atherosclerosis.

Our present data also implicated both the rs2259820 and rs2464196 in T2DM, reaffirming a role for this genomic region in diabetic disease pathways. Interestingly, the rs2464196 and two other variants linked to MI were also implicated in HTN, pointing to a possible common link for the three cardiovascular diseases. To our knowledge, this is the first report linking the rs2259820 to T2DM. This variant resides on exon 7 of the* HNF1a* gene, suggesting that this genic region is important for the manifestation of this disease. However, as in the case of CAD, a number of investigations addressing the role of the* HNF1a* gene polymorphism with respect to diabetic disease conditions have yielded somewhat inconsistent results. Thus, for example, among the variants included in the present study, the rs2259816_G>T has been associated with diabetic nephropathy in type 1 diabetes patients but not with T2DM [19] or other cardiovascular risk traits including dyslipidemia, hypertension, or obesity [40]. On the other hand, a study by Qi et al. (2011) [46] suggested that the combined risk of acquiring CAD in diabetic patients increased significantly in the presence of the rs2259816 and other risk loci related to various genes. As discussed above, in the present study, this variant was linked to MI but only weakly associated with T2DM, adding to the inconsistency in the literature about the impact of the* HNF1a* polymorphism on disease manifestation, in general.

As demonstrated in Figure 1, HNF1a regulates several genes involved in lipoprotein metabolism, such as apolipoproteins, cholesterol synthesizing enzymes, and bile acid transporters as well as glucose-stimulated insulin secretion [1, 7]. Hence, we deemed it necessary to evaluate its potential influence on lipid profiles in our study population. Our data indicates that the variants exhibited even stronger relationships with hTG and the harbouring of hLDLC levels than with the diseases discussed above. The findings of an association with dyslipidemia are in agreement with previous studies implicating some of the* HNF1a* gene variants in dyslipidemia, T2DM [38], hTG, and high low-density lipoprotein-cholesterol (hLDLC) levels [37]. Specifically, the rs2258287 has been linked to acquiring hLDLC levels [37], and a study in the Oji-Cree population described an association of the G319S with “hypertriglycemic waist” contributing to greater risk of T2DM [22]. Besides, mice null for* HNF1a* have also shown altered plasma cholesterol levels [7]. Put together, our findings furnish unequivocal support to the notion that the chromosomal region encompassing the* HNF1a* gene is a susceptibility locus for MI, HTN, T2DM, and dyslipidemia, the important causes for atherosclerosis. This scenario also points to possible complex interactions between diabetes and dyslipidemia in atherosclerosis disease pathways. Similar interaction has been suggested recently between* HNFIa* gene, hyperglycemia, cardiovascular risk, and T2DM [39].

Even more important is the fact that, while the large majority of* HNF1a* variants associated with disease to date are coding, in the present study, it was primarily noncoding SNPs that were implicated. These include the rs2259816 located on intron 7 of the gene, which was associated with MI but was protective against T2DM. These observations seem to point to the likelihood that other entities related to the function of this locus might offer an explanation for these observations. Hence, we thought it worthwhile to explore further the probability of haplotyping this region as being more informative than individual SNPs in establishing the impact of the changes in the* HNF1a* gene locus on disease manifestation. Indeed, our study revealed several stretches of sequences that were associated with MI, T2DM, and changes in triglyceride and LDL-cholesterol levels. Notably, the difference between the causative 7-mer CATATAC and protective CATAGAC for MI was the genomic position of the rs2259816, which displayed the most significant association with the disease. Even more interesting was the observation of an association of the former haplotype with T2DM, demonstrating a link for the two diseases at haplotype level. Thus, MI and T2DM share not only common causative variants but also a common genomic region at the locus of the* HNF1a* gene. However, hTG was also associated with a number of other haplotypes that differed, however, from those implicated in MI and T2DM, suggestive of other independent entities as the possible underlying causes of these disorders. This could occur in various fashions, including the likelihood of the changes influencing gene expression as cis-acting regulators of nearby genes. For potential mechanisms involving SNPs in the 3-untranslated region (3′-UTR), it can be speculated that such genic changes are likely to interfere with actions of gene regulatory elements, such as the microRNAs, thereby influencing mRNA maturation processes, for example. However, this notion needs to be verified further.

We conclude that the* HNF1a* locus constitutes a risk factor for MI, T2DM HTN, and dyslipidemia. Furthermore, since the majority of implicated variants were noncoding, we speculate that some other entities at this genomic locus, rather than the* HNF1A* gene per se, are likely to be the primary contributors to the disease pathways of atherosclerosis.

## Supplementary Material

The HNF1a Suppl data consist of four data sets. The first summarises the important clinical and demographic characteristics and genotyping of the studied heterozygous familial hypercholesterolaemia family. The second set (HNF1a Suppl data 2), is a summary of the Mann-Whitney-Wilcoxon analysis tests, while the third set (HNF1a Suppl data 3) depicts the Bonferroni adjustment for age. In the fourth (HNF1a Suppl data 4) we provide the summary of the Binary logistical regression analysis for the HNF1a gene variants with the disease traits.

## Figures and Tables

**Table:** (a) Demographics of the coronary artery disease set.

	Controls			Cases		
	All	Male	Female	All	Male	Female
CAD	2212	1183 (53.5)	1029 (46.5)	2419	1852 (76.6)	567 (23.4)
Age	47.1 ± 0.4	47.2 ± 0.6	47.1 ± 0.4	59.7 ± 0.2	59.3 ± 0.2	60.8 ± 0.4
BMI	28.9 ± 0.2	29.9 ± 0.2	30.0 ± 0.3	29.2 ± 0.1	28.3 ± 0.1	31.6 ± 0.2
FH	536	293 (54.7)	243 (45.3)	381	307 (80.6)	74 (19.4)
T2DM	893	479 (53.6)	414 (46.4)	1657	1227 (74.0)	430 (26.0)
HTN	1490	774 (51.9)	716 (45.1)	2038	1532 (75.2)	506 (24.8)
OBS	854	363 (42.5)	491 (57.5)	905	601 (66.4)	304 (33.6)
hChol	566	289 (51.1)	277 (48.9)	1113	823 (73.9)	290 (26.1)
hLDLC	272	144 (52.9)	128 (47.1)	359	262 (73.0)	225 (27.0)
lHDLC	695	458 (65.9)	237 (34.1)	1230	1019 (82.8)	448 (17.2)
hTG	402	248 (61.7)	154 (38.3)	758	577 (76.1)	181 (23.9)
Smokers	653	607 (93.0)	46 (7.0)	1108	1079 (97.4)	29 (2.6)
VD						
One	0	0	0	918	667 (72.7)	251 (27.3)
Two	0	0	0	483	378 (78.3)	105 (21.7)
Multiple	0	0	0	1018	807 (79.3)	211 (20.7)

The numbers in brackets give the percentages of the total (all) values of the group. BMI: body mass index; FH: family history of CAD; hLDLC: high low-density lipoprotein-cholesterol level; lHDLC: low high-density lipoprotein-cholesterol level; hTG: hypertriglyceridemia; hChol: hypercholesterolemia; HTN: hypertension; OBS: obesity; T2DM: type 2 diabetes mellitus; VD: number of diseased vessels.

**Table:** (b) Demographics for the studied coronary artery disease traits.

	Controls			Cases		
	All	Male	Female	All	Male	Female
MI	1587	786 (49.5)	801 (50.5)	3044	2249 (97.9)	795 (26.1)
T2DM	2081	1329 (63.9)	752 (36.1)	2550	1706 (66.9)	844 (33.1)
HTN	1103	729 (66.1)	374 (33.9)	3528	2306 (65.4)	1222 (34.6)
OBS	2573	1876 (72.9)	697 (27.1)	1759	964 (54.8)	795 (45.2)
hChol	2733	1793 (65.6)	940 (34.4)	1679	1112 (66.2)	567 (33.8)
hLDLC	3681	2438 (66.2)	1243 (33.8)	631	406 (64.3)	225 (35.7)
lHDLC	2393	1369 (57.2)	1024 (42.8)	1925	1477 (76.7)	448 (23.3)
hTG	3156	2019 (64.0)	1137 (36.0)	1160	825 (71.1)	335 (28.9)

The numbers in brackets give the percentages of the total (all) values of the group. hChol: hypercholesterolemia; hLDLC: high low-density lipoprotein-cholesterol level; hTG: hypertriglyceridemia; HTN: hypertension; lHDLC: low high-density lipoprotein-cholesterol level; OBS: obesity; MI: myocardial infarction; T2DM: type 2 diabetes mellitus.

**Table 2:** Association of hepatic nuclear factor 1 alpha with cardiovascular disease traits.

	*B*	S.E.	Wald	*P* value	Exp (*B*)	95% C.I. for Exp (*B*)	
						Lower	Upper
Myocardial infarction							
rs2259820_CC	0.178	0.087	4.20	0.041	1.19	1.01	1.42
rs2464196_TT	0.171	0.086	4.01	0.045	1.19	1.00	1.40
rs2259816_T	0.123	0.057	4.67	0.031	1.13	1.01	1.26
Type 2 diabetes mellitus							
rs2259820_T	0.129	0.051	6.40	0.011∗	1.14	1.03	1.26
rs2259820_CT+TT	0.178	0.085	4.39	0.036	1.20	1.01	1.41
rs2464196_C	0.114	0.051	5.06	0.024	1.12	1.02	1.24
rs1169310_G	−0.104	0.051	4.17	0.041	0.90	0.82	1.00
Hypertension							
rs2393791_T	0.127	0.059	4.58	0.032	1.14	1.01	1.28
rs7310409_G	0.148	0.059	6.16	0.013∗	1.16	1.03	1.30
rs2259820_T	−0.149	0.059	6.46	0.011∗	0.86	0.77	0.97
rs2464196_AG+GG	0.223	0.088	6.39	0.012∗	1.25	1.05	1.49
Hypertriglyceridemia							
rs2393791_T	0.129	0.054	5.59	0.018∗	1.14	1.02	1.27
rs7310409_G	0.117	0.054	4.64	0.031	1.12	1.01	1.25
rs2259820_C	−0.130	0.054	5.82	0.016∗	0.88	0.79	0.98
rs2464196_T	−0.108	0.054	4.04	0.044	0.90	0.81	1.00
rs2259816_T	−0.117	0.055	4.52	0.034	0.90	0.80	0.99
rs1169310_G	0.140	0.054	6.71	0.010∗	1.15	1.04	1.28
rs1169313_(CT+TT)	0.215	0.080	7.14	0.008∗	1.24	1.06	1.45
High low-density lipoprotein-cholesterol level							
rs2259820_T	0.203	0.070	8.44	0.004∗∗	1.23	1.07	1.41
rs2464196_T	0.198	0.069	8.16	0.004∗∗	1.22	1.06	1.39
rs2259816_T	0.164	0.072	5.19	0.023	1.18	1.02	1.36
rs1169310_G	−0.175	0.071	6.13	0.013∗	0.84	0.73	0.96
rs1169313_T	−0.160	0.071	5.10	0.024	0.85	0.74	0.98

The table lists the variants associated with myocardial infarction (MI; 3044 cases versus 1587 controls), type 2 diabetes mellitus (2550 versus 2081), hypertension (3528 versus 1103), hypertriglyceridemia (1160 versus 3156), and high low-density lipoprotein-cholesterol level (631 versus 3681) among the 4631 studied individuals before and following adjustments for the influences of other risks factors, following Bonferroni correction and adjustment confounding effects of coexisting diseases and covariates (see Supplementary Data). *B*: beta coefficient; C.I.: confidence interval; Exp: exponential; *P* values: adjusted significance value after adjustments for confounders. *P < 0.02; **P < 0.005.

**Table 3:** Association of selected *HNF1a* haplotypes with disease.

Block	Haplotype	Pooled	Cases	Control	*χ* ^2^	*P* value
Myocardial infarction						
1–7	CATATAC	0.505	0.516	0.486	7.50	0.0062∗
	TGCGGGT	0.356	0.346	0.375	7.42	0.0064∗
	CATAGAC	0.011	0.009	0.014	6.06	0.014
1–6	CATATA	0.504	0.514	0.484	7.48	0.0062∗
	TGCGGG	0.357	0.349	0.484	5.81	0.0159
	CATAGA	0.011	0.009	0.015	7.66	0.0056∗
	ATATAC	0.505	0.515	0.486	6.90	0.0086∗
	GCGGGT	0.357	0.347	0.374	6.47	0.011∗
1–5	CATAT	0.503	0.513	0.483	7.68	0.0056∗
	TGCGG	0.361	0.353	0.377	5.22	0.022
	CATAG	0.012	0.009	0.016	7.28	0.007∗
3–7	TATAC	0.514	0.525	0.494	7.97	0.0048
	CGGGT	0.396	0.387	0.412	5.33	0.021
2–6	ATATA	0.504	0.514	0.485	7.02	0.0081∗
	GCGGG	0.359	0.351	0.374	4.94	0.026
2–5	ATAT	0.503	0.513	0.484	6.70	0.0096∗
3–6	TATA	0.514	0.525	0.493	8.36	0.0038∗∗
4–7	ATAC	0.515	0.525	0.496	7.26	0.0071∗
1–4	CATA	0.513	0.521	0.498	4.36	0.037
Hypertriglyceridemia						
1–6	TGCGGG	0.357	0.376	0.352	4.26	0.039
1–5	TGCGG	0.361	0.380	0.355	4.61	0.032
3–7	CGGGT	0.396	0.415	0.390	4.23	0.039
2–6	GCGGG	0.359	0.377	0.353	3.90	0.048
4–7	GGGT	0.395	0.414	0.390	4.27	0.039
Obesity						
2–7	ACGGGT	0.04	0.046	0.036	5.04	0.025
2–6	ACGGG	0.04	0.047	0.036	5.84	0.016
Type 2 diabetes mellitus						
1–7	CATATAC	0.505	0.515	0.494	3.94	0.047
1–4	CATA	0.513	0.523	0.502	3.96	0.047

The table shows selected haplotypes associated with disease. The most frequent 7-mer haplotype CATATAC (0.505) was employed as the baseline to determine the relative effects of the other haplotypes. The studied SNPs are (1) rs2393791, (2) rs7310409, (3) rs2259820, (4) rs2464196, (5) rs2259816, (6) rs1169310, and (7) rs1169313 arranged sequentially by their chromosomal positions, and blocks represent the range of variants constituting the respective haplotypes. *P < 0.01; **P < 0.005 by *χ* ^2^ test.
