# Supplementary material for: A New Susceptibility Locus for Myocardial Infarction, Hypertension, Type 2 Diabetes Mellitus, and Dyslipidemia on Chromosome 12q24
Source: Dis Markers. 2014 Jun 26;2014:291419. doi: 10.1155/2014/291419 (PMC4098619; doi:10.1155/2014/291419)
Supplement: Supplementary file 1 — The HNF1a Suppl data consist of four data sets. The first summarises the important clinical and demographic characteristics and genotyping of the studied heterozygous familial hypercholesterolaemia family. The second set (HNF1a Suppl data 2), is a summary of the Mann-Whitney-Wilcoxon analysis tests, while the third set (HNF1a Suppl data 3) depicts the Bonferroni adjustment for age. In the fourth (HNF1a Suppl data 4) we provide the summary of the Binary logistical regression analysis for the HNF1a gene variants with the disease traits. [file 291419.f1.pdf]

HNF1a Supplementary data

HNF1a Suppl data 1

Important clinical and demographic characteristics and genotyping of the studied heterozygous familial hypercholesterolaemia family

Serial No.	Relation	Sex	Age	Status	Chol	TG	LDL-C	HDL-C	rs2464196T>C	rc2259820C>T	rs1169310A>G	rs2259816G>T	rs1169313 C>T	rs2393791C>T	rs7310409A>G
1001	FT*	1	40	2	6.1*	0.8	4.0	1.06	TC	CT	AG	GT	CT	CT	AG
1002	MT**	2	40	1	6.3**	0.5	4.2**	1.04	CC	CC	GG	GG	TT	TT	GG
1004	S1	1	20	2	5.3	0.4	3.6	0.86**	CC	CC	GG	GG	TT	TT	GG
1007	S2	1	17	0	3.1	0.2	1.5	1.35	TC	CT	AG	GT	CT	CT	AG
**1003**	**S3*** **	**1**	**14**	**1**	**10.1*** *	0.3	**7.9****	**0.51****	TC	CT	AG	GT	CT	CT	AG
1005	S4***	1	12	1	6.2*	1.0	4.4**	0.98**	CC	CC	GG	GG	TT	TT	GG
1008	S5	1	10	0	4.7	0.6	2.6	1.36	TC	CT	AG	GT	CT	CT	AG
1006	S6***	1	9	1	6.9**	1.0	4.6**	1.05	TC	CT	AG	GT	CT	CT	AG
1009	S7	1	3	0	3.3	0.5	1.4	1.61	TC	CT	AG	GT	CT	CT	AG
1010	D2***	2	5	1	6.2*	0.7	4.3**	1.00**	TC	CT	AG	GT	CT	CT	AG
1011	D1	2	15	2	4.8	0.8	3.2	0.67**	CC	CC	GG	GG	TT	TT	GG

Lipid level ranges are: Total Cholesterol, total (Desirable: < 5.2 mmol/L; Borderline high: 5.2 - 6.2 mmol/L; High: ≥ 6.2 mmol/L); Triglycerides (Normal: < 1.7 mmol/L; Borderline high: 1.7 -2.25 mmol/L; High: 2.26 -5.64 mmol/L; Very high:≥ 5.65 mmol/L); LDL-cholesterol (Optimal: < 2.59 mmol/L; Low risk: 2.59 – 3.34 mmol/L; Borderline high: 3.37 – 4.12 mmol/L; high: 4.14 – 4.90 mmol/L; Very high: ≥ 4.92 mmol/L) and HDL-Cholesterol (Low HDL: <1.04 mmol/L; Normal: 1.04 – 1.55 mmol/L; Desirable: > 1.55 mmol/L); *Borderline high total cholesterol levels; **Out-of range levels.

HNF1a Suppl data 2: Mann-Whitney-Wilcoxon analysis; yellow significant following test

a. Grouping Variable: CAD				
	Mann-Whitney U	Wilcoxon W	Z	Asymp. Sig. (2-tailed)
rs2393791C	10480814.000	20268914.000	-2.045	.041
rs2393791T	10492690.000	22198231.000	-1.928	.054
rs7310409T	10587664.000	22293205.000	-1.053	.292
rs7310409C	10587664.000	22293205.000	-1.053	.292
rs2259820C	10617570.000	22323111.000	-.901	.368
rs2259820T	10596740.000	20384840.000	-1.082	.279
rs2464196G	10528486.000	22234027.000	-1.686	.092
rs2464196AA	10528486.000	22234027.000	-1.686	.092
rs2259816T	10457218.000	20245318.000	-2.606	.009
rs2259816G	10457218.000	20245318.000	-2.606	.009
rs1169310AA	10574854.000	20362954.000	-1.190	.234
rs1169310G	10564894.000	22270435.000	-1.280	.201
rs1169313C	10541272.000	20329372.000	-1.511	.131
rs1169313T	10569992.000	22275533.000	-1.225	.221

a. Grouping Variable: MI				
	Mann-Whitney U	Wilcoxon W	Z	Asymp. Sig. (2-tailed)
rs2393791C	9333838.000	14372563.000	-3.195	.001
rs2393791T	9333892.000	27868808.000	-3.183	.001
rs7310409T	9424378.000	27959294.000	-2.307	.021
rs7310409C	9424378.000	27959294.000	-2.307	.021
rs2259820C	9490666.000	28025582.000	-1.927	.054
rs2259820T	9510588.000	14549313.000	-1.640	.101
rs2464196G	9285168.000	27820084.000	-3.858	.000
rs2464196AA	9285168.000	27820084.000	-3.858	.000

rs2259816T	9285028.000	14323753.000	-4.225	.000
rs2259816G	9285028.000	14323753.000	-4.225	.000
rs1169310AA	9342938.000	14381663.000	-3.147	.002
rs1169310G	9347466.000	27882382.000	-3.095	.002
rs1169313C	9348452.000	14387177.000	-3.105	.002
rs1169313T	9318694.000	27853610.000	-3.358	.001

a. Grouping Variable: DM				
	Mann-Whitney U	Wilcoxon W	Z	Asymp. Sig. (2-tailed)
rs2393791C	10397444.000	19060647.000	-2.006	.045
rs2393791T	10409228.000	23416778.000	-1.889	.059
rs7310409T	10464272.000	23471822.000	-1.381	.167
rs7310409C	10464272.000	23471822.000	-1.381	.167
rs2259820C	10308176.000	23315726.000	-3.279	.001
rs2259820T	10310692.000	18973895.000	-3.132	.002
rs2464196G	10470062.000	23477612.000	-1.398	.162
rs2464196AA	10470062.000	23477612.000	-1.398	.162
rs2259816T	10468326.000	19131529.000	-1.550	.121
rs2259816G	10468326.000	19131529.000	-1.550	.121
rs1169310AA	10436188.000	19099391.000	-1.667	.096
rs1169310G	10412682.000	23420232.000	-1.884	.060
rs1169313C	10460104.000	19123307.000	-1.447	.148
rs1169313T	10457880.000	23465430.000	-1.450	.147

a. Grouping Variable: HTN				
	Mann-Whitney U	Wilcoxon W	Z	Asymp. Sig. (2-tailed)
rs2393791C	7600186.000	32497282.000	-1.983	.047
rs2393791T	7594038.000	10028359.000	-2.042	.041

rs7310409T	7551248.000	9985569.000	-2.508	.012
rs7310409C	7551248.000	9985569.000	-2.508	.012
rs2259820C	7715366.000	10149687.000	-.846	.397
rs2259820T	7731150.000	32628246.000	-.624	.532
rs2464196G	7587586.000	10021907.000	-2.228	.026
rs2464196AA	7587586.000	10021907.000	-2.228	.026
rs2259816T	7665958.000	32563054.000	-1.460	.144
rs2259816G	7665958.000	32563054.000	-1.460	.144
rs1169310AA	7637154.000	32534250.000	-1.602	.109
rs1169310G	7642020.000	10076341.000	-1.545	.122
rs1169313C	7658744.000	32555840.000	-1.370	.171
rs1169313T	7606772.000	10041093.000	-1.920	.055

a. Grouping Variable: HCHOL

	Mann-Whitney U	Wilcoxon W	Z	Asymp. Sig. (2-tailed)
rs2393791C	8888734.000	23830045.000	-2.957	.003
rs2393791T	8905842.000	14545603.000	-2.771	.006
rs7310409T	8877728.000	14517489.000	-3.062	.002
rs7310409C	8877728.000	14517489.000	-3.062	.002
rs2259820C	9063288.000	14703049.000	-1.354	.176
rs2259820T	9085130.000	24026441.000	-1.054	.292
rs2464196G	8973010.000	14612771.000	-2.197	.028
rs2464196AA	8973010.000	14612771.000	-2.197	.028
rs2259816T	9127630.000	24068941.000	-.588	.557
rs2259816G	9127630.000	24068941.000	-.588	.557
rs1169310AA	8946656.000	23887967.000	-2.395	.017
rs1169310G	8931042.000	14570803.000	-2.550	.011
rs1169313C	8964378.000	23905689.000	-2.218	.027
rs1169313T	8998520.000	14638281.000	-1.840	.066

a. Grouping Variable: LHDL				
	Mann-Whitney U	Wilcoxon W	Z	Asymp. Sig. (2-tailed)
rs2393791C	9139112.000	20594403.000	-.765	.444
rs2393791T	9131836.000	16545011.000	-.837	.403
rs7310409T	9062642.000	16475817.000	-1.552	.121
rs7310409C	9062642.000	16475817.000	-1.552	.121
rs2259820C	9196824.000	20652115.000	-.194	.846
rs2259820T	9143472.000	16556647.000	-.801	.423
rs2464196G	9204702.000	16617877.000	-.091	.928
rs2464196AA	9204702.000	16617877.000	-.091	.928
rs2259816T	9144682.000	20599973.000	-.813	.416
rs2259816G	9144682.000	20599973.000	-.813	.416
rs1169310AA	9162918.000	20618209.000	-.526	.599
rs1169310G	9175616.000	16588791.000	-.391	.696
rs1169313C	9180914.000	20636205.000	-.338	.735
rs1169313T	9208818.000	16621993.000	-.044	.965

a. Grouping Variable: HLDL				
	Mann-Whitney U	Wilcoxon W	Z	Asymp. Sig. (2-tailed)
rs2393791C	4517876.000	31621079.000	-1.860	.063
rs2393791T	4500416.000	5297369.000	-2.107	.035
rs7310409T	4514930.000	5311883.000	-1.898	.058
rs7310409C	4514930.000	5311883.000	-1.898	.058
rs2259820C	4516110.000	5313063.000	-2.177	.029
rs2259820T	4510226.000	31613429.000	-2.193	.028
rs2464196G	4454788.000	5251741.000	-2.919	.004
rs2464196AA	4454788.000	5251741.000	-2.919	.004

rs2259816T	4611816.000	31715019.000	-.563	.573
rs2259816G	4611816.000	31715019.000	-.563	.573
rs1169310AA	4465716.000	31568919.000	-2.656	.008
rs1169310G	4455198.000	5252151.000	-2.803	.005
rs1169313C	4455200.000	31558403.000	-2.820	.005
rs1169313T	4497264.000	5294217.000	-2.169	.030

a. Grouping Variable: OBS

	Mann-Whitney U	Wilcoxon W	Z	Asymp. Sig. (2-tailed)
rs2393791C	9048958.000	22292189.000	-.030	.976
rs2393791T	9012618.000	15202539.000	-.407	.684
rs7310409T	9017240.000	15207161.000	-.359	.719
rs7310409C	9017240.000	15207161.000	-.359	.719
rs2259820C	8994576.000	22237807.000	-.688	.491
rs2259820T	9026678.000	22269909.000	-.291	.771
rs2464196G	9038122.000	15228043.000	-.150	.881
rs2464196AA	9038122.000	15228043.000	-.150	.881
rs2259816T	8993472.000	15183393.000	-.698	.485
rs2259816G	8993472.000	15183393.000	-.698	.485
rs1169310AA	9032028.000	15221949.000	-.209	.835
rs1169310G	9034498.000	22277729.000	-.182	.855
rs1169313C	9000310.000	15190231.000	-.546	.585
rs1169313T	9018330.000	22261561.000	-.350	.726

HNF1a Suppl data 3: Bonferroni adjustment for age; Blue remains significant after correction; yellow turned non-significant following correction

Dependent Variable: CAD

Parameter	B	Std. Error	t	Sig.	Corrected P-value	95% Confidence Interval	
						Lower Bound	Upper Bound
Intercept	.462	.097	4.782	.000		.273	.651
[AGE_NEW=1] * rs2393791C	-.196	.120	-1.635	.102	.408	-.430	.039
[AGE_NEW=2] * rs2393791C	.195	.132	1.478	.139	.558	-.064	.453
[AGE_NEW=3] * rs2393791C	.030	.114	.263	.793	3.171	-.193	.253
[AGE_NEW=4] * rs2393791C	.405	.135	2.989	.003	.011	.139	.670
[AGE_NEW=5] * rs2393791C	.179	.152	1.174	.240	.961	-.120	.477
[AGE_NEW=1] * rs2393791T	-.212	.119	-1.784	.074	.298	-.445	.021
[AGE_NEW=2] * rs2393791T	.044	.122	.360	.719	2.877	-.195	.282
[AGE_NEW=3] * rs2393791T	.039	.110	.355	.723	2.891	-.176	.254
[AGE_NEW=4] * rs2393791T	.113	.150	.755	.450	1.802	-.181	.408
[AGE_NEW=5] * rs2393791T	.059	.149	.396	.692	2.767	-.233	.351
[AGE_NEW=1] * rs7310409T	.029	.074	.392	.695	2.781	-.117	.175
[AGE_NEW=2] * rs7310409T	.185	.073	2.540	.011	.044	.042	.329
[AGE_NEW=3] * rs7310409T	.020	.071	.283	.777	3.108	-.120	.160
[AGE_NEW=4] * rs7310409T	.196	.071	2.755	.006	.024	.057	.336
[AGE_NEW=5] * rs7310409T	.033	.077	.431	.666	2.666	-.118	.185
[AGE_NEW=1] * rs2259820C	-.128	.072	-1.771	.077	.307	-.269	.014
[AGE_NEW=2] * rs2259820C	-.118	.079	-1.499	.134	.535	-.273	.036
[AGE_NEW=3] * rs2259820C	.158	.080	1.985	.047	.189	.002	.314
[AGE_NEW=4] * rs2259820C	.071	.075	.954	.340	1.360	-.075	.217
[AGE_NEW=5] * rs2259820C	.161	.080	1.999	.046	.183	.003	.318
[AGE_NEW=1] * rs2259820T	-.117	.070	-1.673	.094	.378	-.254	.020
[AGE_NEW=2] * rs2259820T	-.195	.075	-2.599	.009	.037	-.341	-.048
[AGE_NEW=3] * rs2259820T	.179	.076	2.347	.019	.076	.030	.329
[AGE_NEW=4] * rs2259820T	-.034	.073	-.461	.645	2.578	-.177	.110
[AGE_NEW=5] * rs2259820T	.146	.077	1.885	.059	.238	-.006	.298
[AGE_NEW=1] * rs2464196G	3.806E-05	.045	.001	.999	3.997	-.087	.088
[AGE_NEW=2] * rs2464196G	-.040	.046	-.858	.391	1.563	-.130	.051
[AGE_NEW=3] * rs2464196G	.040	.046	.858	.391	1.563	-.051	.131
[AGE_NEW=4] * rs2464196G	-.063	.046	-1.352	.177	.706	-.154	.028

[AGE_NEW=5] * rs2464196G	-.046	.046	-1.012	.311	1.246	-.136	.043
[AGE_NEW=1] * rs2259816T	.070	.034	2.043	.041	.164	.003	.137
[AGE_NEW=2] * rs2259816T	.056	.041	1.374	.169	.678	-.024	.135
[AGE_NEW=3] * rs2259816T	-.011	.043	-.267	.789	3.157	-.095	.072
[AGE_NEW=4] * rs2259816T	.083	.039	2.101	.036	.143	.006	.160
[AGE_NEW=5] * rs2259816T	-.026	.037	-.690	.490	1.962	-.099	.047
[AGE_NEW=1] * rs1169310AA	-.063	.159	-.398	.691	2.764	-.374	.248
[AGE_NEW=2] * rs1169310AA	-.216	.150	-1.439	.150	.600	-.510	.078
[AGE_NEW=3] * rs1169310AA	-.151	.147	-1.026	.305	1.219	-.438	.137
[AGE_NEW=4] * rs1169310AA	-.035	.168	-.209	.834	3.338	-.365	.294
[AGE_NEW=5] * rs1169310AA	.073	.147	.499	.618	2.471	-.215	.362
[AGE_NEW=1] * rs1169310G	-.074	.141	-.527	.598	2.392	-.350	.202
[AGE_NEW=2] * rs1169310G	-.041	.142	-.288	.773	3.092	-.319	.237
[AGE_NEW=3] * rs1169310G	-.092	.137	-.672	.502	2.006	-.360	.176
[AGE_NEW=4] * rs1169310G	-.092	.171	-.535	.593	2.370	-.427	.244
[AGE_NEW=5] * rs1169310G	.093	.142	.653	.514	2.054	-.185	.371
[AGE_NEW=1] * rs1169313C	.087	.095	.916	.359	1.438	-.099	.272
[AGE_NEW=2] * rs1169313C	.217	.095	2.296	.022	.087	.032	.402
[AGE_NEW=3] * rs1169313C	.096	.104	.930	.352	1.410	-.107	.299
[AGE_NEW=4] * rs1169313C	-.213	.124	-1.717	.086	.344	-.456	.030
[AGE_NEW=5] * rs1169313C	-.161	.118	-1.363	.173	.691	-.392	.070
[AGE_NEW=1] * rs1169313T	.068	.079	.869	.385	1.540	-.086	.223
[AGE_NEW=2] * rs1169313T	.031	.091	.337	.736	2.943	-.147	.209
[AGE_NEW=3] * rs1169313T	-.037	.085	-.438	.662	2.647	-.204	.130
[AGE_NEW=4] * rs1169313T	-.042	.108	-.391	.696	2.782	-.254	.169
[AGE_NEW=5] * rs1169313T	-.057	.095	-.599	.549	2.196	-.243	.129

**Dependent Variable: DM**							
Parameter	B	Std. Error	t	Sig.	Corrected P-value	95% Confidence Interval	
						Lower Bound	Upper Bound
Intercept	.571	.095	5.987	.000		.384	.759
[AGE_NEW=1] * rs2393791C	-.261	.118	-2.212	.027	.108	-.493	-.030
[AGE_NEW=2] * rs2393791C	.253	.130	1.946	.052	.207	-.002	.509
[AGE_NEW=3] * rs2393791C	.090	.112	.796	.426	1.704	-.131	.310
[AGE_NEW=4] * rs2393791C	.090	.134	.673	.501	2.003	-.172	.352
[AGE_NEW=5] * rs2393791C	.628	.150	4.176	.000	.000	.333	.923
[AGE_NEW=1] * rs2393791T	-.308	.117	-2.623	.009	.035	-.538	-.078

[AGE_NEW=2] * rs2393791T	.076	.120	.633	.527	2.108	-.160	.312
[AGE_NEW=3] * rs2393791T	.087	.108	.808	.419	1.676	-.125	.300
[AGE_NEW=4] * rs2393791T	.045	.148	.301	.763	3.052	-.246	.336
[AGE_NEW=5] * rs2393791T	.488	.147	3.317	.001	.004	.200	.777
[AGE_NEW=1] * rs7310409T	-.061	.074	-.824	.410	1.640	-.205	.084
[AGE_NEW=2] * rs7310409T	.161	.072	2.232	.026	.103	.020	.303
[AGE_NEW=3] * rs7310409T	.046	.071	.647	.518	2.071	-.093	.184
[AGE_NEW=4] * rs7310409T	.027	.070	.381	.703	2.813	-.111	.165
[AGE_NEW=5] * rs7310409T	.190	.076	2.491	.013	.051	.040	.339
[AGE_NEW=1] * rs2259820C	.090	.071	1.267	.205	.820	-.049	.230
[AGE_NEW=2] * rs2259820C	-.001	.078	-.007	.994	3.977	-.153	.152
[AGE_NEW=3] * rs2259820C	-.120	.079	-1.533	.125	.502	-.275	.034
[AGE_NEW=4] * rs2259820C	-.138	.074	-1.868	.062	.247	-.282	.007
[AGE_NEW=5] * rs2259820C	-.096	.079	-1.203	.229	.916	-.251	.060
[AGE_NEW=1] * rs2259820T	.126	.069	1.821	.069	.275	-.010	.261
[AGE_NEW=2] * rs2259820T	.013	.074	.182	.855	3.421	-.132	.158
[AGE_NEW=3] * rs2259820T	.037	.076	.495	.621	2.483	-.111	.185
[AGE_NEW=4] * rs2259820T	-.084	.072	-1.170	.242	.969	-.226	.057
[AGE_NEW=5] * rs2259820T	-.069	.076	-.900	.368	1.472	-.219	.081
[AGE_NEW=1] * rs2464196G	.004	.044	.080	.936	3.746	-.083	.090
[AGE_NEW=2] * rs2464196G	-.052	.046	-1.143	.253	1.011	-.142	.037
[AGE_NEW=3] * rs2464196G	.119	.046	2.595	.009	.038	.029	.209
[AGE_NEW=4] * rs2464196G	-.012	.046	-.255	.799	3.196	-.102	.078
[AGE_NEW=5] * rs2464196G	-.026	.045	-.573	.566	2.265	-.114	.062
[AGE_NEW=1] * rs2259816T	-.057	.034	-1.689	.091	.365	-.124	.009
[AGE_NEW=2] * rs2259816T	.013	.040	.336	.737	2.948	-.065	.092
[AGE_NEW=3] * rs2259816T	-.117	.042	-2.790	.005	.021	-.200	-.035
[AGE_NEW=4] * rs2259816T	-.054	.039	-1.395	.163	.652	-.130	.022
[AGE_NEW=5] * rs2259816T	.024	.037	.661	.509	2.035	-.048	.097
[AGE_NEW=1] * rs1169310AA	-.145	.157	-.928	.353	1.413	-.453	.162
[AGE_NEW=2] * rs1169310AA	-.244	.148	-1.645	.100	.400	-.534	.047
[AGE_NEW=3] * rs1169310AA	.017	.145	.118	.906	3.625	-.267	.301
[AGE_NEW=4] * rs1169310AA	.172	.166	1.033	.302	1.206	-.154	.497
[AGE_NEW=5] * rs1169310AA	-.199	.145	-1.367	.172	.687	-.484	.086
[AGE_NEW=1] * rs1169310G	.004	.139	.030	.976	3.905	-.269	.277
[AGE_NEW=2] * rs1169310G	-.197	.140	-1.404	.160	.641	-.471	.078

[AGE_NEW=3] * rs1169310G	-.087	.135	-.645	.519	2.076	-.352	.178
[AGE_NEW=4] * rs1169310G	.118	.169	.697	.486	1.942	-.214	.449
[AGE_NEW=5] * rs1169310G	-.358	.140	-2.550	.011	.043	-.632	-.083
[AGE_NEW=1] * rs1169313C	-.006	.093	-.061	.951	3.805	-.189	.177
[AGE_NEW=2] * rs1169313C	-.029	.093	-.314	.753	3.013	-.212	.154
[AGE_NEW=3] * rs1169313C	.069	.102	.678	.498	1.990	-.131	.270
[AGE_NEW=4] * rs1169313C	-.031	.122	-.257	.797	3.188	-.272	.209
[AGE_NEW=5] * rs1169313C	-.285	.117	-2.441	.015	.059	-.513	-.056
[AGE_NEW=1] * rs1169313T	-.078	.078	-.997	.319	1.275	-.230	.075
[AGE_NEW=2] * rs1169313T	-.041	.090	-.456	.648	2.594	-.217	.135
[AGE_NEW=3] * rs1169313T	.020	.084	.242	.809	3.235	-.145	.186
[AGE_NEW=4] * rs1169313T	.077	.107	.722	.470	1.880	-.132	.286
[AGE_NEW=5] * rs1169313T	-.146	.094	-1.555	.120	.480	-.330	.038

Dependent Variable: MI

Parameter	B	Std. Error	t	Sig.	Corrected P-value	95% Confidence Interval	
						Lower Bound	Upper Bound
Intercept	.607	.089	6.785	.000		.432	.782
[AGE_NEW=1] * rs2393791C	-.387	.111	-3.492	.000	.002	-.604	-.170
[AGE_NEW=2] * rs2393791C	.286	.122	2.340	.019	.077	.046	.525
[AGE_NEW=3] * rs2393791C	.098	.105	.927	.354	1.416	-.109	.304
[AGE_NEW=4] * rs2393791C	.263	.125	2.095	.036	.145	.017	.508
[AGE_NEW=5] * rs2393791C	.093	.141	.662	.508	2.033	-.183	.370
[AGE_NEW=1] * rs2393791T	-.444	.110	-4.031	.000	.000	-.659	-.228
[AGE_NEW=2] * rs2393791T	.205	.113	1.820	.069	.275	-.016	.426
[AGE_NEW=3] * rs2393791T	.024	.101	.241	.809	3.237	-.174	.223
[AGE_NEW=4] * rs2393791T	.056	.139	.403	.687	2.748	-.217	.329
[AGE_NEW=5] * rs2393791T	-.004	.138	-.028	.978	3.911	-.274	.267
[AGE_NEW=1] * rs7310409T	.087	.069	1.262	.207	.828	-.048	.222
[AGE_NEW=2] * rs7310409T	.087	.068	1.287	.198	.793	-.046	.220
[AGE_NEW=3] * rs7310409T	.077	.066	1.173	.241	.964	-.052	.207
[AGE_NEW=4] * rs7310409T	.170	.066	2.584	.010	.039	.041	.300
[AGE_NEW=5] * rs7310409T	.094	.071	1.318	.188	.751	-.046	.234
[AGE_NEW=1] * rs2259820C	-.098	.067	-1.471	.141	.565	-.229	.033
[AGE_NEW=2] * rs2259820C	-.164	.073	-2.246	.025	.099	-.307	-.021
[AGE_NEW=3] * rs2259820C	.192	.074	2.608	.009	.036	.048	.336
[AGE_NEW=4] * rs2259820C	.013	.069	.195	.846	3.382	-.122	.149
[AGE_NEW=5] * rs2259820C	.101	.074	1.350	.177	.708	-.045	.246

[AGE_NEW=1] * rs2259820T	-.126	.065	-1.951	.051	.204	-.253	.001
[AGE_NEW=2] * rs2259820T	-.211	.069	-3.050	.002	.009	-.347	-.076
[AGE_NEW=3] * rs2259820T	.120	.071	1.692	.091	.363	-.019	.259
[AGE_NEW=4] * rs2259820T	-.065	.068	-.967	.333	1.334	-.198	.067
[AGE_NEW=5] * rs2259820T	.070	.072	.978	.328	1.313	-.070	.211
[AGE_NEW=1] * rs2464196G	-.129	.041	-3.113	.002	.007	-.210	-.048
[AGE_NEW=2] * rs2464196G	-.033	.043	-.781	.435	1.740	-.118	.051
[AGE_NEW=3] * rs2464196G	-.088	.043	-2.051	.040	.161	-.172	-.004
[AGE_NEW=4] * rs2464196G	-.007	.043	-.168	.866	3.466	-.092	.077
[AGE_NEW=5] * rs2464196G	.031	.042	.728	.466	1.866	-.052	.114
[AGE_NEW=1] * rs2259816T	.080	.032	2.524	.012	.047	.018	.143
[AGE_NEW=2] * rs2259816T	.039	.038	1.043	.297	1.189	-.034	.113
[AGE_NEW=3] * rs2259816T	.069	.039	1.738	.082	.329	-.009	.146
[AGE_NEW=4] * rs2259816T	.083	.036	2.283	.022	.090	.012	.154
[AGE_NEW=5] * rs2259816T	.048	.035	1.403	.161	.642	-.019	.116
[AGE_NEW=1] * rs1169310AA	.340	.147	2.314	.021	.083	.052	.628
[AGE_NEW=2] * rs1169310AA	-.052	.139	-.372	.710	2.839	-.324	.220
[AGE_NEW=3] * rs1169310AA	-.010	.136	-.072	.942	3.770	-.276	.257
[AGE_NEW=4] * rs1169310AA	-.056	.156	-.358	.720	2.881	-.361	.249
[AGE_NEW=5] * rs1169310AA	.019	.136	.136	.892	3.567	-.249	.286
[AGE_NEW=1] * rs1169310G	.308	.130	2.362	.018	.073	.052	.563
[AGE_NEW=2] * rs1169310G	.091	.131	.693	.489	1.954	-.166	.348
[AGE_NEW=3] * rs1169310G	-.030	.127	-.240	.810	3.240	-.279	.218
[AGE_NEW=4] * rs1169310G	-.046	.158	-.293	.769	3.077	-.357	.264
[AGE_NEW=5] * rs1169310G	.024	.131	.181	.856	3.424	-.234	.281
[AGE_NEW=1] * rs1169313C	-.149	.088	-1.707	.088	.352	-.321	.022
[AGE_NEW=2] * rs1169313C	.019	.088	.220	.826	3.303	-.152	.191
[AGE_NEW=3] * rs1169313C	-.095	.096	-.993	.321	1.283	-.283	.093
[AGE_NEW=4] * rs1169313C	-.034	.115	-.292	.770	3.081	-.258	.191
[AGE_NEW=5] * rs1169313C	-.027	.109	-.250	.802	3.210	-.242	.187
[AGE_NEW=1] * rs1169313T	-.086	.073	-1.186	.236	.943	-.229	.056
[AGE_NEW=2] * rs1169313T	-.135	.084	-1.607	.108	.432	-.300	.030
[AGE_NEW=3] * rs1169313T	-.057	.079	-.716	.474	1.896	-.211	.098
[AGE_NEW=4] * rs1169313T	-.023	.100	-.228	.820	3.279	-.219	.173
[AGE_NEW=5] * rs1169313T	-.042	.088	-.475	.635	2.539	-.214	.130

Dependent Variable: HTN

Parameter	B	Std. Error	t	Sig.	Corrected P-value	95% Confidence Interval	
						Lower Bound	Upper Bound
Intercept	.676	.082	8.226	.000		.515	.837
[AGE_NEW=1] * rs2393791C	-.296	.102	-2.911	.004	.014	-.496	-.097
[AGE_NEW=2] * rs2393791C	.227	.112	2.024	.043	.172	.007	.447
[AGE_NEW=3] * rs2393791C	.088	.097	.905	.365	1.461	-.102	.278
[AGE_NEW=4] * rs2393791C	.212	.115	1.844	.065	.261	-.013	.438
[AGE_NEW=5] * rs2393791C	-.012	.130	-.095	.924	3.696	-.266	.242
[AGE_NEW=1] * rs2393791T	-.183	.101	-1.812	.070	.280	-.381	.015
[AGE_NEW=2] * rs2393791T	.122	.104	1.177	.239	.956	-.081	.325
[AGE_NEW=3] * rs2393791T	-.006	.093	-.066	.947	3.789	-.189	.177
[AGE_NEW=4] * rs2393791T	.223	.128	1.741	.082	.327	-.028	.473
[AGE_NEW=5] * rs2393791T	-.107	.127	-.846	.398	1.590	-.356	.141
[AGE_NEW=1] * rs7310409T	-.002	.063	-.030	.976	3.905	-.126	.122
[AGE_NEW=2] * rs7310409T	.123	.062	1.973	.049	.194	.001	.244
[AGE_NEW=3] * rs7310409T	.110	.061	1.814	.070	.279	-.009	.229
[AGE_NEW=4] * rs7310409T	-.064	.061	-1.060	.289	1.157	-.183	.055
[AGE_NEW=5] * rs7310409T	.105	.066	1.599	.110	.439	-.024	.234
[AGE_NEW=1] * rs2259820C	-.193	.061	-3.151	.002	.007	-.314	-.073
[AGE_NEW=2] * rs2259820C	-.017	.067	-.256	.798	3.191	-.149	.114
[AGE_NEW=3] * rs2259820C	-.030	.068	-.446	.656	2.623	-.163	.102
[AGE_NEW=4] * rs2259820C	.005	.063	.081	.935	3.741	-.119	.130
[AGE_NEW=5] * rs2259820C	.225	.068	3.284	.001	.004	.091	.359
[AGE_NEW=1] * rs2259820T	-.109	.059	-1.837	.066	.265	-.226	.007
[AGE_NEW=2] * rs2259820T	.005	.064	.086	.932	3.727	-.119	.130
[AGE_NEW=3] * rs2259820T	.002	.065	.032	.974	3.897	-.125	.130
[AGE_NEW=4] * rs2259820T	-.029	.062	-.470	.639	2.554	-.151	.093
[AGE_NEW=5] * rs2259820T	.175	.066	2.657	.008	.032	.046	.304
[AGE_NEW=1] * rs2464196G	.096	.038	2.524	.012	.046	.021	.170
[AGE_NEW=2] * rs2464196G	.030	.039	.765	.444	1.778	-.047	.107
[AGE_NEW=3] * rs2464196G	.046	.039	1.159	.246	.986	-.032	.123
[AGE_NEW=4] * rs2464196G	.056	.040	1.422	.155	.620	-.021	.134
[AGE_NEW=5] * rs2464196G	-.091	.039	-2.345	.019	.076	-.167	-.015
[AGE_NEW=1] * rs2259816T	-.125	.029	-4.269	.000	.000	-.182	-.067
[AGE_NEW=2] * rs2259816T	-.048	.034	-1.393	.164	.655	-.116	.020
[AGE_NEW=3] * rs2259816T	-.016	.036	-.447	.655	2.621	-.087	.055

[AGE_NEW=4] * rs2259816T	.017	.033	.495	.621	2.483	-.049	.082
[AGE_NEW=5] * rs2259816T	.035	.032	1.097	.273	1.091	-.027	.097
[AGE_NEW=1] * rs1169310AA	.111	.135	.819	.413	1.651	-.154	.375
[AGE_NEW=2] * rs1169310AA	-.288	.128	-2.260	.024	.095	-.538	-.038
[AGE_NEW=3] * rs1169310AA	.077	.125	.617	.538	2.150	-.168	.322
[AGE_NEW=4] * rs1169310AA	.123	.143	.863	.388	1.553	-.157	.404
[AGE_NEW=5] * rs1169310AA	-.127	.125	-1.016	.310	1.239	-.373	.118
[AGE_NEW=1] * rs1169310G	.101	.120	.846	.398	1.590	-.133	.336
[AGE_NEW=2] * rs1169310G	-.300	.121	-2.487	.013	.052	-.536	-.063
[AGE_NEW=3] * rs1169310G	.036	.116	.308	.758	3.032	-.192	.264
[AGE_NEW=4] * rs1169310G	.111	.146	.765	.444	1.776	-.174	.397
[AGE_NEW=5] * rs1169310G	-.021	.121	-.171	.864	3.457	-.257	.216
[AGE_NEW=1] * rs1169313C	.180	.080	2.234	.025	.102	.022	.337
[AGE_NEW=2] * rs1169313C	.159	.080	1.972	.049	.194	.001	.316
[AGE_NEW=3] * rs1169313C	-.010	.088	-.111	.911	3.645	-.183	.163
[AGE_NEW=4] * rs1169313C	-.123	.105	-1.169	.242	.970	-.330	.083
[AGE_NEW=5] * rs1169313C	.139	.100	1.386	.166	.664	-.058	.336
[AGE_NEW=1] * rs1169313T	.059	.067	.885	.376	1.504	-.072	.191
[AGE_NEW=2] * rs1169313T	.115	.077	1.486	.137	.549	-.037	.267
[AGE_NEW=3] * rs1169313T	.010	.073	.135	.893	3.572	-.132	.152
[AGE_NEW=4] * rs1169313T	-.124	.092	-1.349	.177	.709	-.304	.056
[AGE_NEW=5] * rs1169313T	.122	.081	1.513	.130	.521	-.036	.280

Dependent Variable: OBS

Parameter	B	Std. Error	t	Sig.	Corrected P-value	95% Confidence Interval	
						Lower Bound	Upper Bound
Intercept	.496	.101	4.900	.000		.298	.695
[AGE_NEW=1] * rs2393791C	-.342	.125	-2.742	.006	.025	-.586	-.097
[AGE_NEW=2] * rs2393791C	.012	.139	.088	.930	3.718	-.260	.285
[AGE_NEW=3] * rs2393791C	-.130	.125	-1.041	.298	1.191	-.374	.115
[AGE_NEW=4] * rs2393791C	-.146	.141	-1.035	.301	1.203	-.422	.130
[AGE_NEW=5] * rs2393791C	.078	.160	.490	.624	2.498	-.235	.392
[AGE_NEW=1] * rs2393791T	-.242	.124	-1.955	.051	.202	-.484	.001
[AGE_NEW=2] * rs2393791T	-.107	.128	-.838	.402	1.608	-.357	.143
[AGE_NEW=3] * rs2393791T	-.201	.118	-1.702	.089	.355	-.431	.030

[AGE_NEW=4] * rs2393791T	-.050	.159	-.312	.755	3.021	-.361	.262
[AGE_NEW=5] * rs2393791T	-.006	.156	-.037	.970	3.882	-.311	.300
[AGE_NEW=1] * rs7310409T	-.112	.078	-1.447	.148	.592	-.264	.040
[AGE_NEW=2] * rs7310409T	.101	.080	1.272	.203	.813	-.055	.257
[AGE_NEW=3] * rs7310409T	.082	.076	1.084	.279	1.114	-.067	.231
[AGE_NEW=4] * rs7310409T	-.143	.078	-1.830	.067	.269	-.296	.010
[AGE_NEW=5] * rs7310409T	.037	.085	.436	.663	2.650	-.130	.204
[AGE_NEW=1] * rs2259820C	.131	.076	1.731	.083	.334	-.017	.279
[AGE_NEW=2] * rs2259820C	.149	.085	1.758	.079	.315	-.017	.316
[AGE_NEW=3] * rs2259820C	.150	.083	1.803	.071	.286	-.013	.314
[AGE_NEW=4] * rs2259820C	.039	.079	.488	.625	2.502	-.117	.194
[AGE_NEW=5] * rs2259820C	.087	.091	.958	.338	1.352	-.091	.265
[AGE_NEW=1] * rs2259820T	.067	.073	.916	.360	1.439	-.076	.211
[AGE_NEW=2] * rs2259820T	.235	.081	2.904	.004	.015	.076	.393
[AGE_NEW=3] * rs2259820T	.083	.080	1.041	.298	1.192	-.073	.239
[AGE_NEW=4] * rs2259820T	.152	.078	1.941	.052	.209	-.001	.305
[AGE_NEW=5] * rs2259820T	.056	.087	.642	.521	2.084	-.115	.226
[AGE_NEW=1] * rs2464196G	-.021	.047	-.447	.655	2.618	-.113	.071
[AGE_NEW=2] * rs2464196G	.002	.050	.043	.966	3.864	-.096	.100
[AGE_NEW=3] * rs2464196G	-.103	.049	-2.104	.035	.142	-.199	-.007
[AGE_NEW=4] * rs2464196G	.209	.052	4.009	.000	.000	.107	.311
[AGE_NEW=5] * rs2464196G	-.110	.048	-2.278	.023	.091	-.204	-.015
[AGE_NEW=1] * rs2259816T	.020	.036	.553	.580	2.321	-.051	.091
[AGE_NEW=2] * rs2259816T	-.059	.043	-1.383	.167	.667	-.144	.025
[AGE_NEW=3] * rs2259816T	.047	.045	1.047	.295	1.181	-.041	.136
[AGE_NEW=4] * rs2259816T	-.077	.042	-1.829	.067	.270	-.160	.006
[AGE_NEW=5] * rs2259816T	-.009	.040	-.231	.817	3.269	-.088	.069
[AGE_NEW=1] * rs1169310AA	.302	.170	1.781	.075	.300	-.030	.635
[AGE_NEW=2] * rs1169310AA	.097	.159	.608	.543	2.173	-.215	.408
[AGE_NEW=3] * rs1169310AA	-.094	.158	-.593	.553	2.214	-.403	.216
[AGE_NEW=4] * rs1169310AA	-.232	.175	-1.321	.187	.746	-.575	.112
[AGE_NEW=5] * rs1169310AA	-.234	.154	-1.513	.130	.521	-.536	.069
[AGE_NEW=1] * rs1169310G	.241	.147	1.637	.102	.407	-.048	.529
[AGE_NEW=2] * rs1169310G	.025	.149	.164	.869	3.478	-.268	.317
[AGE_NEW=3] * rs1169310G	-.086	.146	-.594	.553	2.211	-.372	.199
[AGE_NEW=4] * rs1169310G	-.137	.179	-.767	.443	1.772	-.487	.213
[AGE_NEW=5] * rs1169310G	-.209	.149	-1.405	.160	.640	-.501	.083

[AGE_NEW=1] * rs1169313C	-.196	.108	-1.817	.069	.277	-.407	.015
[AGE_NEW=2] * rs1169313C	-.313	.104	-3.000	.003	.011	-.517	-.108
[AGE_NEW=3] * rs1169313C	.054	.123	.438	.661	2.645	-.187	.294
[AGE_NEW=4] * rs1169313C	.192	.130	1.486	.137	.549	-.061	.446
[AGE_NEW=5] * rs1169313C	-.030	.124	-.243	.808	3.233	-.273	.213
[AGE_NEW=1] * rs1169313T	-.112	.084	-1.327	.184	.738	-.276	.053
[AGE_NEW=2] * rs1169313T	-.227	.100	-2.265	.024	.094	-.423	-.030
[AGE_NEW=3] * rs1169313T	.116	.099	1.168	.243	.971	-.079	.311
[AGE_NEW=4] * rs1169313T	.012	.112	.107	.915	3.660	-.208	.232
[AGE_NEW=5] * rs1169313T	.078	.099	.791	.429	1.717	-.115	.271

Dependent Variable: hChol

Parameter	B	Std. Error	t	Sig.	Corrected P-value	95% Confidence Interval	
						Lower Bound	Upper Bound
Intercept	.408	.099	4.128	.000		.214	.602
[AGE_NEW=1] * rs2393791C	-.244	.122	-2.005	.045	.180	-.483	-.005
[AGE_NEW=2] * rs2393791C	.402	.140	2.862	.004	.017	.127	.677
[AGE_NEW=3] * rs2393791C	.166	.117	1.418	.156	.624	-.063	.395
[AGE_NEW=4] * rs2393791C	.212	.138	1.543	.123	.492	-.057	.482
[AGE_NEW=5] * rs2393791C	-.007	.156	-.046	.963	3.854	-.313	.299
[AGE_NEW=1] * rs2393791T	-.294	.121	-2.429	.015	.061	-.531	-.057
[AGE_NEW=2] * rs2393791T	.426	.132	3.216	.001	.005	.166	.685
[AGE_NEW=3] * rs2393791T	.062	.112	.553	.580	2.322	-.158	.282
[AGE_NEW=4] * rs2393791T	.260	.154	1.685	.092	.368	-.043	.562
[AGE_NEW=5] * rs2393791T	.017	.152	.109	.913	3.652	-.282	.315
[AGE_NEW=1] * rs7310409T	.065	.076	.854	.393	1.573	-.084	.214
[AGE_NEW=2] * rs7310409T	-.041	.075	-.550	.582	2.329	-.188	.105
[AGE_NEW=3] * rs7310409T	.024	.077	.307	.759	3.036	-.128	.175
[AGE_NEW=4] * rs7310409T	-.120	.076	-1.592	.112	.446	-.269	.028
[AGE_NEW=5] * rs7310409T	-.093	.079	-1.179	.238	.954	-.247	.061
[AGE_NEW=1] * rs2259820C	-.020	.074	-.270	.787	3.148	-.166	.125
[AGE_NEW=2] * rs2259820C	-.075	.082	-.921	.357	1.429	-.236	.085
[AGE_NEW=3] * rs2259820C	-.168	.081	-2.065	.039	.156	-.327	-.009
[AGE_NEW=4] * rs2259820C	-.018	.076	-.243	.808	3.232	-.167	.130
[AGE_NEW=5] * rs2259820C	.022	.091	.236	.814	3.254	-.158	.201
[AGE_NEW=1] * rs2259820T	.033	.072	.465	.642	2.567	-.108	.175
[AGE_NEW=2] * rs2259820T	-.091	.078	-	.241	.963	-.243	.061

			1.173				
[AGE_NEW=3] * rs2259820T	-.127	.078	-1.623	.105	.418	-.279	.026
[AGE_NEW=4] * rs2259820T	-.074	.074	-.998	.318	1.273	-.220	.072
[AGE_NEW=5] * rs2259820T	.062	.087	.706	.480	1.921	-.110	.233
[AGE_NEW=1] * rs2464196G	-.038	.048	-.796	.426	1.703	-.132	.056
[AGE_NEW=2] * rs2464196G	-.005	.048	-.112	.911	3.642	-.099	.088
[AGE_NEW=3] * rs2464196G	-.046	.048	-.959	.338	1.350	-.139	.048
[AGE_NEW=4] * rs2464196G	.088	.049	1.804	.071	.285	-.008	.183
[AGE_NEW=5] * rs2464196G	-.016	.048	-.328	.743	2.970	-.109	.078
[AGE_NEW=1] * rs2259816T	-.049	.036	-1.358	.174	.698	-.120	.022
[AGE_NEW=2] * rs2259816T	.000	.042	-.007	.995	3.979	-.083	.082
[AGE_NEW=3] * rs2259816T	-.033	.045	-.733	.464	1.855	-.121	.055
[AGE_NEW=4] * rs2259816T	.045	.040	1.104	.270	1.078	-.035	.124
[AGE_NEW=5] * rs2259816T	-.044	.039	-1.134	.257	1.027	-.120	.032
[AGE_NEW=1] * rs1169310AA	.261	.165	1.575	.115	.461	-.064	.585
[AGE_NEW=2] * rs1169310AA	-.389	.156	-2.490	.013	.051	-.696	-.083
[AGE_NEW=3] * rs1169310AA	.157	.151	1.043	.297	1.187	-.138	.452
[AGE_NEW=4] * rs1169310AA	-.179	.172	-1.046	.296	1.183	-.516	.157
[AGE_NEW=5] * rs1169310AA	-.282	.151	-1.867	.062	.248	-.578	.014
[AGE_NEW=1] * rs1169310G	.104	.144	.720	.472	1.887	-.179	.387
[AGE_NEW=2] * rs1169310G	-.340	.147	-2.316	.021	.082	-.628	-.052
[AGE_NEW=3] * rs1169310G	.097	.139	.694	.488	1.950	-.176	.370
[AGE_NEW=4] * rs1169310G	-.299	.175	-1.711	.087	.348	-.643	.044
[AGE_NEW=5] * rs1169310G	-.107	.145	-.733	.464	1.855	-.392	.178
[AGE_NEW=1] * rs1169313C	-.198	.103	-1.921	.055	.219	-.401	.004
[AGE_NEW=2] * rs1169313C	.157	.100	1.573	.116	.463	-.039	.353
[AGE_NEW=3] * rs1169313C	-.119	.110	-1.079	.281	1.123	-.335	.097
[AGE_NEW=4] * rs1169313C	.008	.127	.067	.947	3.787	-.241	.257
[AGE_NEW=5] * rs1169313C	.269	.121	2.219	.026	.106	.031	.506
[AGE_NEW=1] * rs1169313T	-.014	.081	-.169	.866	3.463	-.173	.145
[AGE_NEW=2] * rs1169313T	.071	.097	.734	.463	1.852	-.119	.262
[AGE_NEW=3] * rs1169313T	.036	.088	.403	.687	2.748	-.138	.209
[AGE_NEW=4] * rs1169313T	.065	.110	.594	.552	2.209	-.150	.281
[AGE_NEW=5] * rs1169313T	.185	.097	1.915	.056	.222	-.004	.374

Dependent Variable: hTG

Parameter	B	Std. Error	t	Sig.	Corrected P-value	95% Confidence Interval	
						Lower Bound	Upper Bound
Intercept	.179	.091	1.959	.050		-7.131E-05	.358
[AGE_NEW=1] * rs2393791C	-.103	.113	-.914	.361	1.442	-.324	.118
[AGE_NEW=2] * rs2393791C	.513	.132	3.887	.000	.000	.254	.772
[AGE_NEW=3] * rs2393791C	-.013	.108	-.119	.905	3.620	-.225	.199
[AGE_NEW=4] * rs2393791C	.150	.127	1.183	.237	.947	-.099	.400
[AGE_NEW=5] * rs2393791C	.200	.144	1.384	.166	.665	-.083	.482
[AGE_NEW=1] * rs2393791T	-.045	.112	-.402	.687	2.750	-.264	.174
[AGE_NEW=2] * rs2393791T	.432	.124	3.484	.000	.002	.189	.675
[AGE_NEW=3] * rs2393791T	.047	.104	.452	.651	2.605	-.156	.250
[AGE_NEW=4] * rs2393791T	.134	.143	.937	.349	1.395	-.146	.413
[AGE_NEW=5] * rs2393791T	.231	.141	1.641	.101	.403	-.045	.507
[AGE_NEW=1] * rs7310409T	-.042	.070	-.604	.546	2.183	-.180	.095
[AGE_NEW=2] * rs7310409T	-.010	.073	-.133	.894	3.577	-.154	.134
[AGE_NEW=3] * rs7310409T	-.126	.071	-1.763	.078	.312	-.266	.014
[AGE_NEW=4] * rs7310409T	.028	.070	.407	.684	2.737	-.109	.165
[AGE_NEW=5] * rs7310409T	.018	.074	.248	.804	3.215	-.127	.164
[AGE_NEW=1] * rs2259820C	.001	.070	.009	.993	3.971	-.136	.138
[AGE_NEW=2] * rs2259820C	-.146	.078	-1.869	.062	.247	-.299	.007
[AGE_NEW=3] * rs2259820C	-.032	.075	-.425	.671	2.683	-.179	.115
[AGE_NEW=4] * rs2259820C	-.012	.070	-.173	.863	3.451	-.150	.125
[AGE_NEW=5] * rs2259820C	-.042	.085	-.496	.620	2.480	-.208	.124
[AGE_NEW=1] * rs2259820T	.024	.068	.361	.718	2.871	-.108	.157
[AGE_NEW=2] * rs2259820T	-.144	.074	-1.937	.053	.211	-.289	.002
[AGE_NEW=3] * rs2259820T	.032	.072	.445	.656	2.624	-.109	.173
[AGE_NEW=4] * rs2259820T	-.042	.069	-.616	.538	2.153	-.177	.092
[AGE_NEW=5] * rs2259820T	.004	.081	.054	.957	3.828	-.154	.162
[AGE_NEW=1] * rs2464196G	-.070	.045	-1.578	.115	.458	-.158	.017
[AGE_NEW=2] * rs2464196G	.004	.046	.080	.936	3.745	-.086	.093
[AGE_NEW=3] * rs2464196G	.066	.044	1.507	.132	.528	-.020	.153
[AGE_NEW=4] * rs2464196G	-.053	.046	-1.146	.252	1.007	-.142	.037
[AGE_NEW=5] * rs2464196G	.009	.044	.210	.833	3.334	-.078	.097
[AGE_NEW=1] * rs2259816T	-.016	.033	-.465	.642	2.568	-.081	.050
[AGE_NEW=2] * rs2259816T	-.011	.039	-.280	.780	3.118	-.088	.066
[AGE_NEW=3] * rs2259816T	-.064	.042	-1.514	.130	.521	-.147	.019
[AGE_NEW=4] * rs2259816T	-.049	.038	-1.300	.194	.775	-.124	.025
[AGE_NEW=5] * rs2259816T	-.039	.036	-	.285	1.139	-.110	.032

			1.070				
[AGE_NEW=1] * rs1169310AA	.266	.154	1.722	.085	.340	-.037	.568
[AGE_NEW=2] * rs1169310AA	-.296	.146	-2.020	.043	.174	-.583	-.009
[AGE_NEW=3] * rs1169310AA	.097	.139	.696	.486	1.945	-.176	.369
[AGE_NEW=4] * rs1169310AA	.058	.159	.365	.715	2.860	-.253	.369
[AGE_NEW=5] * rs1169310AA	-.065	.139	-.463	.643	2.573	-.338	.209
[AGE_NEW=1] * rs1169310G	.231	.133	1.730	.084	.335	-.031	.492
[AGE_NEW=2] * rs1169310G	-.282	.137	-2.055	.040	.160	-.550	-.013
[AGE_NEW=3] * rs1169310G	.155	.128	1.204	.229	.914	-.097	.407
[AGE_NEW=4] * rs1169310G	-.074	.162	-.455	.649	2.595	-.392	.244
[AGE_NEW=5] * rs1169310G	-.102	.134	-.759	.448	1.792	-.365	.161
[AGE_NEW=1] * rs1169313C	-.146	.097	-1.513	.130	.521	-.336	.043
[AGE_NEW=2] * rs1169313C	.066	.094	.705	.481	1.924	-.118	.250
[AGE_NEW=3] * rs1169313C	.062	.102	.614	.539	2.157	-.137	.262
[AGE_NEW=4] * rs1169313C	-.050	.118	-.422	.673	2.691	-.281	.182
[AGE_NEW=5] * rs1169313C	-.085	.112	-.757	.449	1.797	-.304	.134
[AGE_NEW=1] * rs1169313T	-.027	.075	-.356	.722	2.886	-.174	.121
[AGE_NEW=2] * rs1169313T	.192	.092	2.078	.038	.151	.011	.373
[AGE_NEW=3] * rs1169313T	.008	.082	.092	.927	3.706	-.152	.167
[AGE_NEW=4] * rs1169313T	.118	.102	1.161	.246	.983	-.081	.318
[AGE_NEW=5] * rs1169313T	-.066	.089	-.745	.457	1.826	-.241	.108

Dependent Variable: lHDL

Parameter	B	Std. Error	t	Sig.	Corrected P-value	95% Confidence Interval	
						Lower Bound	Upper Bound
Intercept	.489	.103	4.768	.000		.288	.690
[AGE_NEW=1] * rs2393791C	-.401	.127	-3.170	.002	.006	-.649	-.153
[AGE_NEW=2] * rs2393791C	.059	.148	.402	.688	2.752	-.231	.350
[AGE_NEW=3] * rs2393791C	-.035	.121	-.291	.771	3.084	-.274	.203
[AGE_NEW=4] * rs2393791C	.066	.143	.465	.642	2.567	-.213	.346
[AGE_NEW=5] * rs2393791C	-.067	.162	-.417	.677	2.708	-.385	.250
[AGE_NEW=1] * rs2393791T	-.414	.125	-3.307	.001	.004	-.660	-.169
[AGE_NEW=2] * rs2393791T	-.025	.139	-.180	.857	3.430	-.298	.248
[AGE_NEW=3] * rs2393791T	.227	.116	1.951	.051	.204	-.001	.455
[AGE_NEW=4] * rs2393791T	.091	.160	.569	.569	2.277	-.222	.405
[AGE_NEW=5] * rs2393791T	.081	.158	.514	.607	2.429	-.228	.391

[AGE_NEW=1] * rs7310409T	-.108	.079	-1.365	.172	.689	-.262	.047
[AGE_NEW=2] * rs7310409T	.031	.082	.375	.708	2.830	-.130	.192
[AGE_NEW=3] * rs7310409T	-.307	.080	-3.829	.000	.001	-.464	-.150
[AGE_NEW=4] * rs7310409T	-.111	.078	-1.415	.157	.628	-.265	.043
[AGE_NEW=5] * rs7310409T	-.006	.083	-.075	.941	3.762	-.170	.157
[AGE_NEW=1] * rs2259820C	-.222	.078	-2.824	.005	.019	-.375	-.068
[AGE_NEW=2] * rs2259820C	.009	.088	.106	.916	3.662	-.163	.181
[AGE_NEW=3] * rs2259820C	.073	.084	.870	.384	1.537	-.092	.239
[AGE_NEW=4] * rs2259820C	-.086	.079	-1.093	.274	1.097	-.240	.068
[AGE_NEW=5] * rs2259820C	-.140	.095	-1.477	.140	.558	-.327	.046
[AGE_NEW=1] * rs2259820T	-.275	.076	-3.627	.000	.001	-.424	-.127
[AGE_NEW=2] * rs2259820T	-.058	.083	-.700	.484	1.935	-.222	.105
[AGE_NEW=3] * rs2259820T	.147	.081	1.814	.070	.279	-.012	.305
[AGE_NEW=4] * rs2259820T	-.103	.077	-1.331	.183	.733	-.254	.049
[AGE_NEW=5] * rs2259820T	-.173	.090	-1.909	.056	.225	-.350	.005
[AGE_NEW=1] * rs2464196G	-.009	.050	-.173	.863	3.451	-.107	.089
[AGE_NEW=2] * rs2464196G	-.075	.051	-1.472	.141	.565	-.175	.025
[AGE_NEW=3] * rs2464196G	.185	.049	3.732	.000	.001	.088	.282
[AGE_NEW=4] * rs2464196G	.023	.051	.452	.651	2.606	-.078	.124
[AGE_NEW=5] * rs2464196G	.006	.050	.121	.903	3.614	-.092	.104
[AGE_NEW=1] * rs2259816T	.071	.037	1.896	.058	.232	-.002	.144
[AGE_NEW=2] * rs2259816T	.089	.044	2.002	.045	.181	.002	.176
[AGE_NEW=3] * rs2259816T	-.097	.048	-2.028	.043	.170	-.190	-.003
[AGE_NEW=4] * rs2259816T	.014	.043	.319	.750	3.000	-.070	.097
[AGE_NEW=5] * rs2259816T	-.004	.041	-.088	.930	3.721	-.083	.076
[AGE_NEW=1] * rs1169310AA	.360	.173	2.081	.037	.150	.021	.700
[AGE_NEW=2] * rs1169310AA	.066	.164	.404	.686	2.744	-.256	.388
[AGE_NEW=3] * rs1169310AA	.021	.156	.134	.893	3.573	-.285	.326
[AGE_NEW=4] * rs1169310AA	-.161	.178	-.906	.365	1.460	-.511	.188
[AGE_NEW=5] * rs1169310AA	.171	.157	1.094	.274	1.095	-.136	.478
[AGE_NEW=1] * rs1169310G	.306	.150	2.047	.041	.163	.013	.600
[AGE_NEW=2] * rs1169310G	.120	.154	.778	.436	1.746	-.182	.421
[AGE_NEW=3] * rs1169310G	-.029	.144	-.202	.840	3.360	-.312	.254
[AGE_NEW=4] * rs1169310G	.020	.182	.110	.913	3.651	-.337	.377
[AGE_NEW=5] * rs1169310G	-.077	.151	-.514	.607	2.430	-.373	.218

[AGE_NEW=1] * rs1169313C	.150	.108	1.388	.165	.660	-.062	.363
[AGE_NEW=2] * rs1169313C	-.156	.105	-1.479	.139	.557	-.363	.051
[AGE_NEW=3] * rs1169313C	-.068	.114	-.595	.552	2.208	-.292	.156
[AGE_NEW=4] * rs1169313C	.130	.133	.983	.326	1.303	-.130	.390
[AGE_NEW=5] * rs1169313C	.036	.125	.284	.777	3.107	-.210	.281
[AGE_NEW=1] * rs1169313T	.303	.084	3.588	.000	.001	.138	.469
[AGE_NEW=2] * rs1169313T	-.101	.104	-.973	.330	1.322	-.304	.102
[AGE_NEW=3] * rs1169313T	-.145	.092	-1.582	.114	.455	-.324	.035
[AGE_NEW=4] * rs1169313T	.035	.114	.302	.762	3.049	-.189	.258
[AGE_NEW=5] * rs1169313T	.120	.100	1.203	.229	.916	-.076	.316

Dependent Variable: hLDL

Parameter	B	Std. Error	t	Sig.	Corrected P-value	95% Confidence Interval	
						Lower Bound	Upper Bound
Intercept	.262	.073	3.584	.000		.119	.406
[AGE_NEW=1] * rs2393791C	-.044	.090	-.489	.625	2.499	-.221	.133
[AGE_NEW=2] * rs2393791C	-.110	.106	-1.041	.298	1.191	-.317	.097
[AGE_NEW=3] * rs2393791C	-.041	.087	-.471	.638	2.552	-.211	.129
[AGE_NEW=4] * rs2393791C	-.004	.102	-.039	.969	3.877	-.203	.196
[AGE_NEW=5] * rs2393791C	-.346	.115	-2.996	.003	.011	-.572	-.120
[AGE_NEW=1] * rs2393791T	-.144	.089	-1.609	.108	.431	-.319	.031
[AGE_NEW=2] * rs2393791T	.104	.099	1.049	.294	1.177	-.090	.299
[AGE_NEW=3] * rs2393791T	-.116	.083	-1.396	.163	.651	-.278	.047
[AGE_NEW=4] * rs2393791T	-.020	.114	-.179	.858	3.432	-.244	.203
[AGE_NEW=5] * rs2393791T	-.264	.113	-2.340	.019	.077	-.484	-.043
[AGE_NEW=1] * rs7310409T	.061	.056	1.083	.279	1.115	-.049	.171
[AGE_NEW=2] * rs7310409T	-.120	.059	-2.046	.041	.163	-.235	-.005
[AGE_NEW=3] * rs7310409T	.087	.057	1.519	.129	.516	-.025	.199
[AGE_NEW=4] * rs7310409T	.043	.056	.769	.442	1.767	-.067	.153
[AGE_NEW=5] * rs7310409T	-.092	.060	-1.539	.124	.495	-.208	.025
[AGE_NEW=1] * rs2259820C	.001	.056	.011	.991	3.965	-.109	.110
[AGE_NEW=2] * rs2259820C	-.009	.063	-.141	.888	3.552	-.132	.114
[AGE_NEW=3] * rs2259820C	-.087	.060	-1.448	.148	.591	-.205	.031
[AGE_NEW=4] * rs2259820C	.046	.056	.811	.417	1.669	-.065	.156
[AGE_NEW=5] * rs2259820C	-.019	.068	-.286	.775	3.098	-.152	.113
[AGE_NEW=1] * rs2259820T	-.002	.054	-.034	.973	3.893	-.108	.104

[AGE_NEW=2] * rs2259820T	-.005	.059	-.080	.936	3.744	-.121	.112
[AGE_NEW=3] * rs2259820T	-.021	.058	-.370	.711	2.845	-.135	.092
[AGE_NEW=4] * rs2259820T	.049	.055	.894	.371	1.485	-.059	.157
[AGE_NEW=5] * rs2259820T	.054	.065	.837	.403	1.610	-.072	.181
[AGE_NEW=1] * rs2464196G	.004	.036	.101	.920	3.679	-.066	.073
[AGE_NEW=2] * rs2464196G	-.010	.037	-.270	.787	3.150	-.082	.062
[AGE_NEW=3] * rs2464196G	.002	.036	.054	.957	3.826	-.068	.072
[AGE_NEW=4] * rs2464196G	-.087	.037	-2.370	.018	.071	-.159	-.015
[AGE_NEW=5] * rs2464196G	-.016	.036	-.455	.649	2.596	-.086	.054
[AGE_NEW=1] * rs2259816T	.000	.027	-.013	.990	3.959	-.053	.052
[AGE_NEW=2] * rs2259816T	-.038	.032	-1.196	.232	.927	-.101	.024
[AGE_NEW=3] * rs2259816T	-.030	.034	-.865	.387	1.549	-.097	.037
[AGE_NEW=4] * rs2259816T	-.021	.030	-.699	.484	1.937	-.080	.038
[AGE_NEW=5] * rs2259816T	-.019	.029	-.638	.524	2.095	-.076	.039
[AGE_NEW=1] * rs1169310AA	-.101	.124	-.817	.414	1.655	-.343	.141
[AGE_NEW=2] * rs1169310AA	-.055	.118	-.465	.642	2.567	-.286	.176
[AGE_NEW=3] * rs1169310AA	-.082	.111	-.739	.460	1.839	-.300	.136
[AGE_NEW=4] * rs1169310AA	-.137	.127	-1.084	.278	1.113	-.386	.111
[AGE_NEW=5] * rs1169310AA	-.019	.112	-.174	.862	3.447	-.238	.199
[AGE_NEW=1] * rs1169310G	-.101	.107	-.944	.345	1.382	-.310	.109
[AGE_NEW=2] * rs1169310G	-.068	.110	-.620	.535	2.141	-.283	.147
[AGE_NEW=3] * rs1169310G	-.089	.103	-.862	.389	1.554	-.290	.113
[AGE_NEW=4] * rs1169310G	-.225	.129	-1.741	.082	.327	-.479	.028
[AGE_NEW=5] * rs1169310G	.038	.108	.350	.726	2.905	-.173	.249
[AGE_NEW=1] * rs1169313C	.042	.077	.540	.589	2.358	-.110	.193
[AGE_NEW=2] * rs1169313C	.096	.076	1.267	.205	.820	-.053	.245
[AGE_NEW=3] * rs1169313C	.075	.081	.926	.355	1.419	-.084	.235
[AGE_NEW=4] * rs1169313C	.047	.094	.498	.618	2.473	-.137	.231
[AGE_NEW=5] * rs1169313C	.217	.089	2.425	.015	.061	.042	.392
[AGE_NEW=1] * rs1169313T	.044	.060	.733	.464	1.854	-.074	.162
[AGE_NEW=2] * rs1169313T	.054	.074	.730	.466	1.862	-.092	.200
[AGE_NEW=3] * rs1169313T	.041	.065	.629	.529	2.118	-.087	.169
[AGE_NEW=4] * rs1169313T	.140	.081	1.723	.085	.340	-.019	.299
[AGE_NEW=5] * rs1169313T	.172	.071	2.405	.016	.065	.032	.311

HNF1a Suppl data 4

Binary logistical analysis

The data shows the summary of the binary logistic regression analysis for the HNF1a gene variants with the disease traits. A, R and D symbols next to the variant stand for allele, recessive and dominant mode, respectively; MI, myocardial infarction; DM, type 2 diabetes mellitus, HTN, hypertension, hChol, hypercholesterolemia; hTG, hypertriglyceridemia; lHDL, low high density lipoprotein; FH, family history of coronary artery disease; hLDL, high low density lipoprotein-cholesterol, BMI, body-mass index; OBS, obesity

HNFA vs Myocardial infarction

rs2393791A MI

Variables in the Equation

		B	S.E.	Wald	df	Sig.	Exp(B)	95% C.I.for EXP(B)	
								Lower	Upper
Step 1[a]	rs2393791A(1)	.092	.056	2.717	1	.099	1.097	.983	1.224
	DM(1)	-.700	.059	138.788	1	.000	.496	.442	.558
	HTN(1)	-.270	.067	16.029	1	.000	.764	.669	.871
	hChol(1)	-.634	.066	91.681	1	.000	.531	.466	.604
	hTG(1)	-.204	.066	9.422	1	.002	.815	.716	.929
	lHDL(1)	-.412	.058	50.957	1	.000	.662	.592	.742
	Sex(1)	1.053	.060	310.915	1	.000	2.866	2.550	3.222
	FH(1)	.272	.066	16.768	1	.000	1.313	1.153	1.496
	hLDL(1)	.373	.084	19.929	1	.000	1.452	1.233	1.710
	BMI	-.004	.007	.377	1	.539	.996	.982	1.010
	Age	.056	.002	615.245	1	.000	1.057	1.053	1.062
	OBS(1)	-.005	.088	.004	1	.951	.995	.837	1.182
	Constant	-2.262	.309	53.489	1	.000	.104		

a. Variable(s) entered on step 1: rs2393791A, DM, HTN, hChol, hTG, lHDL, Sex, FH, hLDL, BMI, Age, OBS.

rs7310409A vs MI

Variables in the Equation

		B	S.E.	Wald	df	Sig.	Exp(B)	95% C.I.for EXP(B)	
								Lower	Upper
Step 1[a]	rs2393791R(1)	.085	.104	.668	1	.414	1.088	.888	1.333
	Sex(1)	1.054	.084	155.611	1	.000	2.868	2.431	3.385
	DM(1)	-.701	.084	69.546	1	.000	.496	.421	.585
	HTN(1)	-.269	.095	7.979	1	.005	.764	.634	.921
	OBS(1)	-.006	.124	.002	1	.964	.994	.779	1.269
	FH(1)	.272	.094	8.372	1	.004	1.313	1.092	1.578
	hChol(1)	-.634	.094	45.940	1	.000	.530	.442	.637
	hTG(1)	-.202	.094	4.639	1	.031	.817	.679	.982
	lHDL(1)	-.413	.082	25.557	1	.000	.662	.564	.777
	hLDL(1)	.371	.118	9.892	1	.002	1.450	1.150	1.828
	Age	.056	.003	307.128	1	.000	1.057	1.051	1.064
	BMI	-.004	.010	.190	1	.663	.996	.976	1.016
	Constant	-2.274	.443	26.377	1	.000	.103		

a. Variable(s) entered on step 1: rs2393791R, Sex, DM, HTN, OBS, FH, hChol, hTG, lHDL, hLDL, Age, BMI.

Variables in the Equation

		B	S.E.	Wald	df	Sig.	Exp(B)	95% C.I.for EXP(B)	
								Lower	Upper
Step 1[a]	rs2393791D	-.139	.081	2.962	1	.085	.871	.743	1.019
	Sex(1)	1.052	.084	155.168	1	.000	2.865	2.427	3.381
	DM(1)	-.699	.084	69.048	1	.000	.497	.422	.586
	HTN(1)	-.272	.095	8.130	1	.004	.762	.632	.919
	OBS(1)	-.005	.124	.002	1	.968	.995	.780	1.270
	FH(1)	.273	.094	8.403	1	.004	1.313	1.092	1.579
	hChol(1)	-.632	.094	45.645	1	.000	.531	.442	.638
	hTG(1)	-.208	.094	4.888	1	.027	.812	.675	.977
	lHDL(1)	-.410	.082	25.282	1	.000	.663	.565	.778
	hLDL(1)	.376	.118	10.127	1	.001	1.456	1.155	1.836
	Age	.056	.003	307.971	1	.000	1.057	1.051	1.064
	BMI	-.004	.010	.184	1	.668	.996	.976	1.016
	Constant	-1.987	.453	19.289	1	.000	.137		

a. Variable(s) entered on step 1: rs2393791D, Sex, DM, HTN, OBS, FH, hChol, hTG, lHDL, hLDL, Age, BMI.

rs7310409A vs MI

Variables in the Equation

		B	S.E.	Wald	df	Sig.	Exp(B)	95% C.I.for EXP(B)	
								Lower	Upper
Step 1[a]	rs7310409A	-.063	.056	1.276	1	.259	.939	.841	1.048
	DM(1)	-.709	.059	143.254	1	.000	.492	.438	.553
	HTN(1)	-.285	.067	18.131	1	.000	.752	.659	.857
	hChol(1)	-.640	.066	93.707	1	.000	.528	.463	.600
	hTG(1)	-.199	.066	9.027	1	.003	.819	.720	.933
	lHDL(1)	-.415	.057	52.128	1	.000	.660	.590	.739
	Sex(1)	1.057	.060	315.741	1	.000	2.879	2.562	3.235
	FH(1)	.267	.066	16.237	1	.000	1.307	1.147	1.488
	hLDL(1)	.368	.083	19.552	1	.000	1.445	1.228	1.702
	BMI	-.005	.007	.569	1	.451	.995	.981	1.009
	Age	.055	.002	607.529	1	.000	1.057	1.052	1.061
	OBS(1)	-.023	.088	.066	1	.797	.978	.823	1.161
	Constant	-2.035	.315	41.620	1	.000	.131		

a. Variable(s) entered on step 1: rs7310409A, DM, HTN, hChol, hTG, lHDL, Sex, FH, hLDL, BMI, Age, OBS.

Variables in the Equation

		B	S.E.	Wald	df	Sig.	Exp(B)	95% C.I.for EXP(B)	
								Lower	Upper
Step 1[a]	rs7310409R	-.097	.104	.872	1	.350	.907	.740	1.113
	Sex(1)	1.059	.084	158.137	1	.000	2.883	2.444	3.400
	DM(1)	-.709	.084	71.791	1	.000	.492	.418	.580
	HTN(1)	-.286	.095	9.102	1	.003	.751	.624	.905
	OBS(1)	-.024	.124	.037	1	.848	.977	.766	1.245
	FH(1)	.267	.094	8.087	1	.004	1.306	1.086	1.570
	hChol(1)	-.640	.093	46.894	1	.000	.527	.439	.633
	hTG(1)	-.199	.094	4.501	1	.034	.820	.682	.985
	lHDL(1)	-.415	.081	26.089	1	.000	.660	.563	.774
	hLDL(1)	.368	.118	9.747	1	.002	1.445	1.147	1.820
	Age	.055	.003	303.426	1	.000	1.057	1.050	1.063
	BMI	-.006	.010	.292	1	.589	.994	.975	1.015
	Constant	-2.006	.450	19.840	1	.000	.135		

a. Variable(s) entered on step 1: rs7310409R, Sex, DM, HTN, OBS, FH, hChol, hTG, lHDL, hLDL, Age, BMI.

Variables in the Equation

		B	S.E.	Wald	df	Sig.	Exp(B)	95% C.I.for EXP(B)	
								Lower	Upper
Step 1[a]	rs7310409D	-.072	.080	.803	1	.370	.931	.796	1.089
	Sex(1)	1.057	.084	157.723	1	.000	2.878	2.440	3.394
	DM(1)	-.708	.084	71.417	1	.000	.493	.418	.581
	HTN(1)	-.286	.095	9.111	1	.003	.751	.624	.905
	OBS(1)	-.022	.124	.030	1	.862	.979	.768	1.248
	FH(1)	.268	.094	8.138	1	.004	1.307	1.087	1.571
	hChol(1)	-.639	.093	46.764	1	.000	.528	.439	.634
	hTG(1)	-.201	.094	4.574	1	.032	.818	.681	.983
	lHDL(1)	-.414	.081	25.935	1	.000	.661	.564	.775
	hLDL(1)	.369	.118	9.833	1	.002	1.447	1.149	1.823
	Age	.055	.003	303.828	1	.000	1.057	1.050	1.063
	BMI	-.005	.010	.277	1	.599	.995	.975	1.015
	Constant	-2.011	.450	19.971	1	.000	.134		

a. Variable(s) entered on step 1: rs7310409D, Sex, DM, HTN, OBS, FH, hChol, hTG, lHDL, hLDL, Age, BMI.

rs2259820A vs MI

Variables in the Equation

		B	S.E.	Wald	df	Sig.	Exp(B)	95% C.I.for EXP(B)	
								Lower	Upper
Step 1[a]	rs2259820A	.074	.056	1.761	1	.185	1.077	.965	1.201
	DM(1)	-.726	.060	146.242	1	.000	.484	.430	.544
	HTN(1)	-.263	.068	14.975	1	.000	.768	.672	.878
	hChol(1)	-.626	.067	86.863	1	.000	.535	.469	.610
	hTG(1)	-.195	.067	8.373	1	.004	.823	.721	.939
	lHDL(1)	-.413	.058	50.101	1	.000	.662	.590	.742
	Sex(1)	1.072	.060	316.289	1	.000	2.922	2.596	3.288
	FH(1)	.266	.067	15.664	1	.000	1.304	1.144	1.488
	hLDL(1)	.352	.085	17.285	1	.000	1.422	1.205	1.679
	BMI	.000	.007	.000	1	.985	1.000	.986	1.014
	Age	.056	.002	606.304	1	.000	1.058	1.053	1.063
	OBS(1)	.022	.089	.059	1	.808	1.022	.858	1.217
	Constant	-2.477	.325	58.083	1	.000	.084		

a. Variable(s) entered on step 1: rs2259820A, DM, HTN, hChol, hTG, lHDL, Sex, FH, hLDL, BMI, Age, OBS.

Variables in the Equation

		B	S.E.	Wald	df	Sig.	Exp(B)	95% C.I.for EXP(B)	
								Lower	Upper
Step 1[a]	rs2259820R	.178	.087	4.196	1	.041	1.194	1.008	1.415
	Sex(1)	1.072	.085	157.842	1	.000	2.920	2.471	3.452
	DM(1)	-.724	.085	72.674	1	.000	.485	.411	.573
	HTN(1)	-.268	.096	7.722	1	.005	.765	.633	.924
	OBS(1)	.026	.126	.042	1	.838	1.026	.802	1.313
	FH(1)	.267	.095	7.879	1	.005	1.306	1.084	1.573
	hChol(1)	-.624	.095	43.171	1	.000	.536	.445	.645
	hTG(1)	-.201	.095	4.466	1	.035	.818	.678	.986
	lHDL(1)	-.412	.082	24.930	1	.000	.662	.564	.779
	hLDL(1)	.359	.120	8.948	1	.003	1.431	1.132	1.810
	Age	.056	.003	303.155	1	.000	1.058	1.051	1.065
	BMI	.000	.010	.000	1	.992	1.000	.980	1.021
	Constant	-2.610	.459	32.318	1	.000	.074		

a. Variable(s) entered on step 1: rs2259820R, Sex, DM, HTN, OBS, FH, hChol, hTG, lHDL, hLDL, Age, BMI.

Variables in the Equation

		B	S.E.	Wald	df	Sig.	Exp(B)	95% C.I.for EXP(B)	
								Lower	Upper
Step 1[a]	rs2259820D	.000	.093	.000	1	.996	1.000	.834	1.198
	Sex(1)	1.072	.085	158.079	1	.000	2.921	2.471	3.452
	DM(1)	-.728	.085	73.455	1	.000	.483	.409	.570
	HTN(1)	-.261	.096	7.335	1	.007	.771	.638	.930
	OBS(1)	.021	.126	.027	1	.869	1.021	.798	1.307
	FH(1)	.267	.095	7.890	1	.005	1.306	1.084	1.573
	hChol(1)	-.626	.095	43.490	1	.000	.535	.444	.644
	hTG(1)	-.192	.095	4.071	1	.044	.825	.685	.995
	lHDL(1)	-.413	.082	25.100	1	.000	.662	.563	.778
	hLDL(1)	.349	.120	8.485	1	.004	1.417	1.121	1.792
	Age	.056	.003	303.192	1	.000	1.058	1.051	1.065
	BMI	.000	.010	.000	1	.984	1.000	.980	1.020
	Constant	-2.360	.471	25.072	1	.000	.094		

a. Variable(s) entered on step 1: rs2259820D, Sex, DM, HTN, OBS, FH, hChol, hTG, lHDL, hLDL, Age, BMI.

rs7310409A vs MI

Variables in the Equation

		B	S.E.	Wald	df	Sig.	Exp(B)	95% C.I.for EXP(B)	
								Lower	Upper
Step 1[a]	rs7310409A	-.063	.056	1.276	1	.259	.939	.841	1.048
	DM(1)	-.709	.059	143.254	1	.000	.492	.438	.553
	HTN(1)	-.285	.067	18.131	1	.000	.752	.659	.857
	hChol(1)	-.640	.066	93.707	1	.000	.528	.463	.600
	hTG(1)	-.199	.066	9.027	1	.003	.819	.720	.933
	lHDL(1)	-.415	.057	52.128	1	.000	.660	.590	.739
	Sex(1)	1.057	.060	315.741	1	.000	2.879	2.562	3.235
	FH(1)	.267	.066	16.237	1	.000	1.307	1.147	1.488
	hLDL(1)	.368	.083	19.552	1	.000	1.445	1.228	1.702
	BMI	-.005	.007	.569	1	.451	.995	.981	1.009
	Age	.055	.002	607.529	1	.000	1.057	1.052	1.061
	OBS(1)	-.023	.088	.066	1	.797	.978	.823	1.161
	Constant	-2.035	.315	41.620	1	.000	.131		

a. Variable(s) entered on step 1: rs7310409A, DM, HTN, hChol, hTG, lHDL, Sex, FH, hLDL, BMI, Age, OBS.

Variables in the Equation

		B	S.E.	Wald	df	Sig.	Exp(B)	95% C.I.for EXP(B)	
								Lower	Upper
Step 1[a]	rs7310409R	-.097	.104	.872	1	.350	.907	.740	1.113
	Sex(1)	1.059	.084	158.137	1	.000	2.883	2.444	3.400
	DM(1)	-.709	.084	71.791	1	.000	.492	.418	.580
	HTN(1)	-.286	.095	9.102	1	.003	.751	.624	.905
	OBS(1)	-.024	.124	.037	1	.848	.977	.766	1.245
	FH(1)	.267	.094	8.087	1	.004	1.306	1.086	1.570
	hChol(1)	-.640	.093	46.894	1	.000	.527	.439	.633
	hTG(1)	-.199	.094	4.501	1	.034	.820	.682	.985
	lHDL(1)	-.415	.081	26.089	1	.000	.660	.563	.774
	hLDL(1)	.368	.118	9.747	1	.002	1.445	1.147	1.820
	Age	.055	.003	303.426	1	.000	1.057	1.050	1.063
	BMI	-.006	.010	.292	1	.589	.994	.975	1.015
	Constant	-2.006	.450	19.840	1	.000	.135		

a. Variable(s) entered on step 1: rs7310409R, Sex, DM, HTN, OBS, FH, hChol, hTG, lHDL, hLDL, Age, BMI.

Variables in the Equation

		B	S.E.	Wald	df	Sig.	Exp(B)	95% C.I.for EXP(B)	
								Lower	Upper
Step 1[a]	rs7310409D	-.072	.080	.803	1	.370	.931	.796	1.089
	Sex(1)	1.057	.084	157.723	1	.000	2.878	2.440	3.394
	DM(1)	-.708	.084	71.417	1	.000	.493	.418	.581
	HTN(1)	-.286	.095	9.111	1	.003	.751	.624	.905
	OBS(1)	-.022	.124	.030	1	.862	.979	.768	1.248
	FH(1)	.268	.094	8.138	1	.004	1.307	1.087	1.571
	hChol(1)	-.639	.093	46.764	1	.000	.528	.439	.634
	hTG(1)	-.201	.094	4.574	1	.032	.818	.681	.983
	lHDL(1)	-.414	.081	25.935	1	.000	.661	.564	.775
	hLDL(1)	.369	.118	9.833	1	.002	1.447	1.149	1.823
	Age	.055	.003	303.828	1	.000	1.057	1.050	1.063
	BMI	-.005	.010	.277	1	.599	.995	.975	1.015
	Constant	-2.011	.450	19.971	1	.000	.134		

a. Variable(s) entered on step 1: rs7310409D, Sex, DM, HTN, OBS, FH, hChol, hTG, lHDL, hLDL, Age, BMI.

rs2464196A vs MI

Variables in the Equation

		B	S.E.	Wald	df	Sig.	Exp(B)	95% C.I.for EXP(B)	
								Lower	Upper
Step 1[a]	rs2464196A(1)	.076	.055	1.901	1	.168	1.079	.969	1.202
	Age	.057	.002	644.637	1	.000	1.058	1.054	1.063
	BMI	-.007	.007	.818	1	.366	.993	.980	1.008
	Sex(1)	1.048	.060	309.727	1	.000	2.852	2.538	3.205
	DM(1)	-.753	.059	164.293	1	.000	.471	.420	.528
	HTN(1)	-.275	.067	16.670	1	.000	.759	.665	.867
	OBS(1)	-.018	.088	.042	1	.838	.982	.827	1.167
	hChol(1)	-.626	.066	89.637	1	.000	.535	.470	.609
	hTG(1)	-.200	.066	9.023	1	.003	.819	.719	.933
	lHDL(1)	-.406	.058	49.622	1	.000	.666	.595	.746
	hLDL(1)	.397	.083	22.658	1	.000	1.487	1.263	1.751
	Constant	-2.029	.305	44.379	1	.000	.131		

a. Variable(s) entered on step 1: rs2464196A, Age, BMI, Sex, DM, HTN, OBS, hChol, hTG, lHDL, hLDL.

Variables in the Equation

		B	S.E.	Wald	df	Sig.	Exp(B)	95% C.I.for EXP(B)	
								Lower	Upper
Step 1[a]	rs2464196R	-.004	.092	.002	1	.963	.996	.832	1.192
	Sex(1)	1.061	.084	158.049	1	.000	2.891	2.450	3.411
	DM(1)	-.720	.084	73.275	1	.000	.487	.413	.574
	HTN(1)	-.271	.095	8.118	1	.004	.763	.633	.919
	OBS(1)	-.017	.124	.020	1	.888	.983	.770	1.254
	FH(1)	.275	.094	8.523	1	.004	1.317	1.095	1.585
	hChol(1)	-.638	.094	46.144	1	.000	.529	.440	.635
	hTG(1)	-.208	.094	4.888	1	.027	.812	.675	.977
	lHDL(1)	-.402	.082	24.269	1	.000	.669	.570	.785
	hLDL(1)	.387	.118	10.707	1	.001	1.472	1.168	1.856
	Age	.055	.003	304.585	1	.000	1.057	1.050	1.064
	BMI	-.005	.010	.225	1	.636	.995	.975	1.015
	Constant	-2.183	.449	23.633	1	.000	.113		

a. Variable(s) entered on step 1: rs2464196R, Sex, DM, HTN, OBS, FH, hChol, hTG, lHDL, hLDL, Age, BMI.

rs2464196D vs MI

Variables in the Equation

		B	S.E.	Wald	df	Sig.	Exp(B)	95% C.I.for EXP(B)	
								Lower	Upper
Step 1[a]	rs2464196D	-.171	.086	4.010	1	.045	.842	.712	.996
	Sex(1)	1.062	.084	158.067	1	.000	2.893	2.452	3.414
	DM(1)	-.716	.084	72.574	1	.000	.489	.414	.576
	HTN(1)	-.279	.095	8.549	1	.003	.757	.628	.912
	OBS(1)	-.014	.124	.012	1	.911	.986	.773	1.258
	FH(1)	.275	.094	8.511	1	.004	1.317	1.095	1.584
	hChol(1)	-.636	.094	45.920	1	.000	.529	.440	.636
	hTG(1)	-.216	.094	5.229	1	.022	.806	.670	.970
	lHDL(1)	-.400	.082	24.035	1	.000	.670	.571	.786
	hLDL(1)	.395	.118	11.128	1	.001	1.484	1.177	1.872
	Age	.056	.003	304.409	1	.000	1.057	1.051	1.064
	BMI	-.005	.010	.200	1	.655	.995	.976	1.016
	Constant	-1.912	.456	17.603	1	.000	.148		

a. Variable(s) entered on step 1: rs2464196D, Sex, DM, HTN, OBS, FH, hChol, hTG, lHDL, hLDL, Age, BMI.

rs2259816A vs MI

Variables in the Equation

		B	S.E.	Wald	df	Sig.	Exp(B)	95% C.I.for EXP(B)	
								Lower	Upper
Step 1[a]	rs2259816A	.123	.057	4.665	1	.031	1.130	1.011	1.263
	Age	.056	.002	605.309	1	.000	1.058	1.053	1.063
	BMI	-.005	.007	.432	1	.511	.995	.981	1.010
	Sex(1)	1.045	.061	293.214	1	.000	2.844	2.523	3.205
	DM(1)	-.711	.060	139.555	1	.000	.491	.437	.553
	HTN(1)	-.303	.069	19.516	1	.000	.739	.646	.845
	OBS(1)	-.007	.090	.007	1	.934	.993	.832	1.184
	hChol(1)	-.640	.068	88.864	1	.000	.527	.462	.602
	hTG(1)	-.187	.068	7.514	1	.006	.830	.726	.948
	lHDL(1)	-.419	.059	50.539	1	.000	.658	.586	.738
	hLDL(1)	.383	.086	19.796	1	.000	1.467	1.239	1.736
	Constant	-2.186	.325	45.280	1	.000	.112		

a. Variable(s) entered on step 1: rs2259816A, Age, BMI, Sex, DM, HTN, OBS, hChol, hTG, lHDL, hLDL.

Variables in the Equation

		B	S.E.	Wald	df	Sig.	Exp(B)	95% C.I.for EXP(B)	
								Lower	Upper
Step 1[a]	rs2259816R	.160	.084	3.611	1	.057	1.174	.995	1.385
	Sex(1)	1.060	.087	149.689	1	.000	2.885	2.435	3.419
	DM(1)	-.674	.086	61.329	1	.000	.510	.431	.603
	HTN(1)	-.303	.097	9.774	1	.002	.739	.611	.893
	OBS(1)	-.002	.128	.000	1	.990	.998	.778	1.282
	FH(1)	.289	.096	9.037	1	.003	1.336	1.106	1.613
	hChol(1)	-.650	.096	45.480	1	.000	.522	.432	.631
	hTG(1)	-.199	.097	4.255	1	.039	.819	.678	.990
	lHDL(1)	-.413	.084	24.473	1	.000	.662	.562	.779
	hLDL(1)	.376	.122	9.459	1	.002	1.456	1.146	1.850
	Age	.055	.003	285.350	1	.000	1.057	1.050	1.064
	BMI	-.003	.010	.063	1	.802	.997	.977	1.018
	Constant	-2.440	.464	27.684	1	.000	.087		

a. Variable(s) entered on step 1: rs2259816R, Sex, DM, HTN, OBS, FH, hChol, hTG, lHDL, hLDL, Age, BMI.

Variables in the Equation

		B	S.E.	Wald	df	Sig.	Exp(B)	95% C.I.for EXP(B)	
								Lower	Upper
Step 1[a]	rs2259816D	.146	.100	2.131	1	.144	1.157	.951	1.407
	Sex(1)	1.062	.087	150.304	1	.000	2.891	2.440	3.426
	DM(1)	-.675	.086	61.615	1	.000	.509	.430	.603
	HTN(1)	-.302	.097	9.696	1	.002	.740	.612	.894
	OBS(1)	-.007	.128	.003	1	.957	.993	.773	1.275
	FH(1)	.292	.096	9.187	1	.002	1.339	1.109	1.616
	hChol(1)	-.652	.096	45.839	1	.000	.521	.431	.629
	hTG(1)	-.194	.097	4.039	1	.044	.824	.682	.995
	lHDL(1)	-.414	.083	24.589	1	.000	.661	.561	.779
	hLDL(1)	.372	.122	9.262	1	.002	1.451	1.142	1.843
	Age	.055	.003	284.499	1	.000	1.057	1.050	1.063
	BMI	-.003	.010	.077	1	.781	.997	.977	1.018
	Constant	-2.470	.482	26.214	1	.000	.085		

a. Variable(s) entered on step 1: rs2259816D, Sex, DM, HTN, OBS, FH, hChol, hTG, lHDL, hLDL, Age, BMI.

rs1169310A vs MI

Variables in the Equation

		B	S.E.	Wald	df	Sig.	Exp(B)	95% C.I.for EXP(B)	
								Lower	Upper
Step 1[a]	rs1169310A	-.097	.056	3.064	1	.080	.907	.814	1.012
	Age	.057	.002	645.097	1	.000	1.058	1.054	1.063
	BMI	-.007	.007	1.012	1	.315	.993	.979	1.007
	Sex(1)	1.054	.059	314.338	1	.000	2.870	2.555	3.225
	DM(1)	-.738	.059	158.377	1	.000	.478	.426	.536
	HTN(1)	-.286	.067	18.167	1	.000	.751	.658	.857
	OBS(1)	-.027	.088	.096	1	.757	.973	.820	1.156
	hChol(1)	-.629	.066	90.618	1	.000	.533	.468	.607
	hTG(1)	-.198	.066	8.960	1	.003	.820	.720	.934
	lHDL(1)	-.413	.057	51.561	1	.000	.662	.591	.741
	hLDL(1)	.388	.083	21.665	1	.000	1.475	1.252	1.737
	Constant	-1.808	.311	33.848	1	.000	.164		

a. Variable(s) entered on step 1: rs1169310A, Age, BMI, Sex, DM, HTN, OBS, hChol, hTG, lHDL, hLDL.

Variables in the Equation

		B	S.E.	Wald	df	Sig.	Exp(B)	95% C.I.for EXP(B)	
								Lower	Upper
Step 1[a]	rs1169310R	-.089	.099	.809	1	.368	.915	.753	1.111
	Sex(1)	1.068	.084	160.274	1	.000	2.908	2.465	3.431
	DM(1)	-.705	.084	70.654	1	.000	.494	.419	.582
	HTN(1)	-.285	.095	9.025	1	.003	.752	.624	.906
	OBS(1)	-.029	.124	.053	1	.818	.972	.762	1.239
	FH(1)	.259	.094	7.554	1	.006	1.295	1.077	1.558
	hChol(1)	-.641	.094	46.663	1	.000	.527	.438	.633
	hTG(1)	-.206	.094	4.818	1	.028	.814	.677	.978
	lHDL(1)	-.409	.081	25.320	1	.000	.664	.566	.779
	hLDL(1)	.380	.118	10.305	1	.001	1.462	1.159	1.844
	Age	.055	.003	305.248	1	.000	1.057	1.050	1.064
	BMI	-.006	.010	.320	1	.571	.994	.975	1.014
	Constant	-2.023	.448	20.369	1	.000	.132		

a. Variable(s) entered on step 1: rs1169310R, Sex, DM, HTN, OBS, FH, hChol, hTG, lHDL, hLDL, Age, BMI.

Variables in the Equation

		B	S.E.	Wald	df	Sig.	Exp(B)	95% C.I.for EXP(B)	
								Lower	Upper
Step 1[a]	rs1169310D	-.145	.082	3.175	1	.075	.865	.737	1.015
	Sex(1)	1.068	.084	160.198	1	.000	2.909	2.465	3.432
	DM(1)	-.703	.084	70.235	1	.000	.495	.420	.584
	HTN(1)	-.288	.095	9.173	1	.002	.750	.623	.904
	OBS(1)	-.024	.124	.038	1	.846	.976	.766	1.245
	FH(1)	.258	.094	7.515	1	.006	1.294	1.076	1.557
	hChol(1)	-.638	.094	46.296	1	.000	.528	.440	.635
	hTG(1)	-.213	.094	5.112	1	.024	.808	.672	.972
	lHDL(1)	-.408	.081	25.076	1	.000	.665	.567	.780
	hLDL(1)	.385	.118	10.597	1	.001	1.470	1.166	1.854
	Age	.055	.003	305.541	1	.000	1.057	1.050	1.064
	BMI	-.005	.010	.278	1	.598	.995	.975	1.015
	Constant	-1.909	.450	17.999	1	.000	.148		

a. Variable(s) entered on step 1: rs1169310D, Sex, DM, HTN, OBS, FH, hChol, hTG, lHDL, hLDL, Age, BMI.

rs1169313A vs MI

Variables in the Equation

		B	S.E.	Wald	df	Sig.	Exp(B)	95% C.I.for EXP(B)	
								Lower	Upper
Step 1[a]	rs1169313A	-.091	.056	2.611	1	.106	.913	.818	1.019
	Age	.058	.002	646.241	1	.000	1.059	1.055	1.064
	BMI	-.006	.007	.676	1	.411	.994	.980	1.008
	Sex(1)	1.059	.060	312.935	1	.000	2.884	2.565	3.244
	DM(1)	-.738	.059	155.174	1	.000	.478	.426	.537
	HTN(1)	-.267	.068	15.406	1	.000	.765	.670	.875
	OBS(1)	-.002	.088	.001	1	.981	.998	.839	1.187
	hChol(1)	-.631	.067	89.500	1	.000	.532	.467	.606
	hTG(1)	-.178	.067	7.063	1	.008	.837	.734	.954
	lHDL(1)	-.440	.058	57.650	1	.000	.644	.575	.721
	hLDL(1)	.374	.084	19.912	1	.000	1.454	1.233	1.713
	Constant	-1.916	.315	37.048	1	.000	.147		

a. Variable(s) entered on step 1: rs1169313A, Age, BMI, Sex, DM, HTN, OBS, hChol, hTG, lHDL, hLDL.

Variables in the Equation

		B	S.E.	Wald	df	Sig.	Exp(B)	95% C.I.for EXP(B)	
								Lower	Upper
Step 1[a]	rs1169313R	-.052	.100	.273	1	.601	.949	.780	1.155
	Sex(1)	1.071	.085	159.259	1	.000	2.919	2.472	3.448
	DM(1)	-.708	.085	69.803	1	.000	.493	.417	.582
	HTN(1)	-.266	.096	7.653	1	.006	.766	.635	.925
	OBS(1)	-.003	.125	.001	1	.982	.997	.780	1.275
	FH(1)	.245	.095	6.666	1	.010	1.278	1.061	1.539
	hChol(1)	-.642	.095	45.976	1	.000	.526	.437	.634
	hTG(1)	-.183	.095	3.746	1	.053	.832	.691	1.002
	lHDL(1)	-.438	.082	28.437	1	.000	.645	.549	.758
	hLDL(1)	.366	.119	9.466	1	.002	1.442	1.142	1.820
	Age	.056	.003	306.944	1	.000	1.058	1.051	1.065
	BMI	-.005	.010	.203	1	.652	.995	.975	1.016
	Constant	-2.155	.454	22.499	1	.000	.116		

a. Variable(s) entered on step 1: rs1169313R, Sex, DM, HTN, OBS, FH, hChol, hTG, lHDL, hLDL, Age, BMI.

Variables in the Equation

		B	S.E.	Wald	df	Sig.	Exp(B)	95% C.I.for EXP(B)	
								Lower	Upper
Step 1[a]	rs1169313D	-.158	.082	3.684	1	.055	.854	.726	1.003
	Sex(1)	1.072	.085	159.286	1	.000	2.921	2.473	3.450
	DM(1)	-.705	.085	69.285	1	.000	.494	.418	.583
	HTN(1)	-.269	.096	7.806	1	.005	.764	.633	.923
	OBS(1)	.001	.125	.000	1	.995	1.001	.783	1.279
	FH(1)	.245	.095	6.646	1	.010	1.278	1.060	1.539
	hChol(1)	-.640	.095	45.661	1	.000	.528	.438	.635
	hTG(1)	-.192	.095	4.102	1	.043	.825	.685	.994
	lHDL(1)	-.436	.082	28.148	1	.000	.647	.551	.760
	hLDL(1)	.373	.119	9.831	1	.002	1.452	1.150	1.833
	Age	.056	.003	307.256	1	.000	1.058	1.051	1.065
	BMI	-.004	.010	.168	1	.682	.996	.976	1.016
	Constant	-1.977	.455	18.853	1	.000	.138		

a. Variable(s) entered on step 1: rs1169313D, Sex, DM, HTN, OBS, FH, hChol, hTG, lHDL, hLDL, Age, BMI.

HNF1a vs Diabetes mellitus

rs2393791A

Variables in the Equation

		B	S.E.	Wald	df	Sig.	Exp(B)	95% C.I.for EXP(B)	
								Lower	Upper
Step 1[a]	rs2393791A	-.067	.051	1.717	1	.190	.935	.845	1.034
	MI	.778	.058	178.548	1	.000	2.177	1.942	2.440
	Age	.036	.002	301.474	1	.000	1.037	1.032	1.041
	BMI	.043	.007	36.779	1	.000	1.043	1.029	1.058
	Sex(1)	-.084	.058	2.107	1	.147	.919	.821	1.030
	HTN(1)	-1.010	.064	251.830	1	.000	.364	.322	.413
	OBS(1)	.021	.082	.068	1	.795	1.021	.870	1.199
	hChol(1)	-.264	.058	20.648	1	.000	.768	.685	.861
	hTG(1)	-.562	.060	86.512	1	.000	.570	.506	.642
	lHDL(1)	-.290	.053	30.237	1	.000	.749	.675	.830
	hLDL(1)	.129	.077	2.829	1	.093	1.138	.979	1.323
	Constant	-2.527	.297	72.379	1	.000	.080		

a. Variable(s) entered on step 1: rs2393791A, MI, Age, BMI, Sex, HTN, OBS, hChol, hTG, lHDL, hLDL.

Variables in the Equation

		B	S.E.	Wald	df	Sig.	Exp(B)	95% C.I.for EXP(B)	
								Lower	Upper
Step 1[a]	rs2393791R(1)	.047	.096	.238	1	.626	1.048	.868	1.265
	Sex(1)	-.052	.083	.402	1	.526	.949	.807	1.116
	HTN(1)	-.985	.091	118.307	1	.000	.374	.313	.446
	OBS(1)	.016	.117	.019	1	.890	1.016	.808	1.278
	FH(1)	.761	.091	69.311	1	.000	2.140	1.789	2.560
	hChol(1)	-.287	.083	11.868	1	.001	.750	.637	.884
	hTG(1)	-.587	.087	45.990	1	.000	.556	.469	.659
	lHDL(1)	-.278	.075	13.658	1	.000	.757	.654	.878
	hLDL(1)	.105	.110	.909	1	.340	1.111	.895	1.378
	Age	.032	.003	118.546	1	.000	1.033	1.027	1.039
	BMI	.047	.010	21.704	1	.000	1.048	1.027	1.069
	MI(1)	-.733	.083	77.527	1	.000	.480	.408	.566
	Constant	-2.388	.429	30.949	1	.000	.092		

a. Variable(s) entered on step 1: rs2393791R, Sex, HTN, OBS, FH, hChol, hTG, lHDL, hLDL, Age, BMI, MI.

Variables in the Equation

		B	S.E.	Wald	df	Sig.	Exp(B)	95% C.I.for EXP(B)	
								Lower	Upper
Step 1[a]	rs2393791D	-.111	.074	2.226	1	.136	.895	.773	1.035
	MI(1)	-.730	.083	76.806	1	.000	.482	.409	.567
	Sex(1)	-.053	.083	.412	1	.521	.948	.807	1.115
	HTN(1)	-.987	.091	118.798	1	.000	.373	.312	.445
	OBS(1)	.018	.117	.024	1	.878	1.018	.810	1.280
	FH(1)	.762	.091	69.520	1	.000	2.143	1.791	2.563
	hChol(1)	-.285	.083	11.661	1	.001	.752	.639	.886
	hTG(1)	-.591	.087	46.491	1	.000	.554	.467	.656
	lHDL(1)	-.277	.075	13.520	1	.000	.758	.654	.879
	hLDL(1)	.108	.110	.962	1	.327	1.114	.898	1.383
	Age	.032	.003	119.001	1	.000	1.033	1.027	1.039
	BMI	.047	.010	21.850	1	.000	1.048	1.028	1.069
	Constant	-2.181	.437	24.886	1	.000	.113		

a. Variable(s) entered on step 1: rs2393791D, MI, Sex, HTN, OBS, FH, hChol, hTG, lHDL, hLDL, Age, BMI.

rs7310409A vs DM

Variables in the Equation

		B	S.E.	Wald	df	Sig.	Exp(B)	95% C.I.for EXP(B)	
								Lower	Upper
Step 1[a]	MI	.786	.058	183.665	1	.000	2.194	1.959	2.458
	Age	.036	.002	300.862	1	.000	1.036	1.032	1.041
	BMI	.042	.007	36.444	1	.000	1.043	1.029	1.058
	Sex(1)	-.087	.058	2.301	1	.129	.916	.818	1.026
	HTN(1)	-1.006	.063	251.460	1	.000	.366	.323	.414
	OBS(1)	.033	.081	.165	1	.684	1.034	.881	1.212
	hChol(1)	-.266	.058	21.022	1	.000	.767	.685	.859
	hTG(1)	-.575	.060	91.097	1	.000	.563	.500	.633
	lHDL(1)	-.281	.052	28.732	1	.000	.755	.681	.837
	hLDL(1)	.128	.077	2.776	1	.096	1.136	.978	1.320
	rs7310409A	-.052	.051	1.011	1	.315	.950	.859	1.050
	Constant	-2.538	.296	73.768	1	.000	.079		

a. Variable(s) entered on step 1: MI, Age, BMI, Sex, HTN, OBS, hChol, hTG, lHDL, hLDL, rs7310409A.

Variables in the Equation

		B	S.E.	Wald	df	Sig.	Exp(B)	95% C.I.for EXP(B)	
								Lower	Upper
Step 1[a]	rs7310409R	-.002	.097	.000	1	.984	.998	.826	1.207
	MI(1)	-.741	.083	79.809	1	.000	.477	.405	.561
	Sex(1)	-.057	.082	.480	1	.488	.945	.804	1.110
	HTN(1)	-.980	.090	117.831	1	.000	.375	.315	.448
	OBS(1)	.027	.116	.054	1	.816	1.027	.818	1.290
	FH(1)	.773	.091	71.807	1	.000	2.167	1.812	2.592
	hChol(1)	-.290	.083	12.150	1	.000	.748	.636	.881
	hTG(1)	-.601	.086	48.373	1	.000	.548	.463	.650
	lHDL(1)	-.270	.075	13.023	1	.000	.763	.659	.884
	hLDL(1)	.104	.110	.904	1	.342	1.110	.895	1.376
	Age	.032	.003	118.105	1	.000	1.033	1.027	1.038
	BMI	.046	.010	21.448	1	.000	1.047	1.027	1.068
	Constant	-2.333	.436	28.623	1	.000	.097		

a. Variable(s) entered on step 1: rs7310409R, MI, Sex, HTN, OBS, FH, hChol, hTG, lHDL, hLDL, Age, BMI.

Variables in the Equation

		B	S.E.	Wald	df	Sig.	Exp(B)	95% C.I.for EXP(B)	
								Lower	Upper
Step 1[a]	rs7310409D	-.103	.074	1.924	1	.165	.902	.780	1.043
	MI(1)	-.740	.083	79.436	1	.000	.477	.406	.562
	Sex(1)	-.057	.082	.474	1	.491	.945	.804	1.110
	HTN(1)	-.984	.090	118.727	1	.000	.374	.313	.446
	OBS(1)	.030	.116	.069	1	.793	1.031	.821	1.295
	FH(1)	.774	.091	71.953	1	.000	2.169	1.814	2.594
	hChol(1)	-.287	.083	11.915	1	.001	.750	.638	.883
	hTG(1)	-.604	.086	48.908	1	.000	.547	.461	.647
	lHDL(1)	-.268	.075	12.747	1	.000	.765	.661	.886
	hLDL(1)	.108	.110	.959	1	.327	1.113	.898	1.381
	Age	.032	.003	117.938	1	.000	1.032	1.027	1.038
	BMI	.047	.010	21.688	1	.000	1.048	1.027	1.068
	Constant	-2.181	.435	25.177	1	.000	.113		

a. Variable(s) entered on step 1: rs7310409D, MI, Sex, HTN, OBS, FH, hChol, hTG, lHDL, hLDL, Age, BMI.

rs2259820A vs DM

Variables in the Equation

		B	S.E.	Wald	df	Sig.	Exp(B)	95% C.I.for EXP(B)	
								Lower	Upper
Step 1[a]	rs2259820A	.129	.051	6.395	1	.011	1.138	1.029	1.257
	MI	.801	.059	185.436	1	.000	2.228	1.985	2.500
	Age	.035	.002	278.926	1	.000	1.036	1.031	1.040
	BMI	.042	.007	34.988	1	.000	1.043	1.029	1.058
	Sex(1)	-.091	.058	2.441	1	.118	.913	.814	1.023
	HTN(1)	-1.025	.064	253.867	1	.000	.359	.316	.407
	OBS(1)	.029	.083	.124	1	.725	1.029	.876	1.210
	hChol(1)	-.274	.059	21.675	1	.000	.760	.678	.853
	hTG(1)	-.586	.061	91.573	1	.000	.557	.494	.628
	lHDL(1)	-.278	.053	27.418	1	.000	.757	.682	.840
	hLDL(1)	.130	.078	2.816	1	.093	1.139	.978	1.327
	Constant	-2.753	.303	82.280	1	.000	.064		

a. Variable(s) entered on step 1: rs2259820A, MI, Age, BMI, Sex, HTN, OBS, hChol, hTG, lHDL, hLDL.

Variables in the Equation

		B	S.E.	Wald	df	Sig.	Exp(B)	95% C.I.for EXP(B)	
								Lower	Upper
Step 1[a]	rs2259820R	.137	.080	2.965	1	.085	1.147	.981	1.341
	MI(1)	-.754	.084	80.292	1	.000	.470	.399	.555
	Sex(1)	-.063	.083	.564	1	.453	.939	.798	1.106
	HTN(1)	-1.000	.092	119.280	1	.000	.368	.307	.440
	OBS(1)	.024	.118	.042	1	.838	1.024	.813	1.291
	FH(1)	.756	.092	67.287	1	.000	2.130	1.778	2.552
	hChol(1)	-.297	.084	12.407	1	.000	.743	.630	.876
	hTG(1)	-.612	.088	48.659	1	.000	.542	.456	.644
	lHDL(1)	-.268	.076	12.497	1	.000	.765	.659	.887
	hLDL(1)	.110	.111	.967	1	.326	1.116	.897	1.388
	Age	.031	.003	109.696	1	.000	1.032	1.026	1.038
	BMI	.046	.010	20.438	1	.000	1.047	1.026	1.068
	Constant	-2.430	.443	30.044	1	.000	.088		

a. Variable(s) entered on step 1: rs2259820R, MI, Sex, HTN, OBS, FH, hChol, hTG, lHDL, hLDL, Age, BMI.

Variables in the Equation

		B	S.E.	Wald	df	Sig.	Exp(B)	95% C.I.for EXP(B)	
								Lower	Upper
Step 1[a]	rs2259820D	.178	.085	4.385	1	.036	1.195	1.011	1.413
	MI(1)	-.759	.084	81.413	1	.000	.468	.397	.552
	Sex(1)	-.062	.083	.549	1	.459	.940	.799	1.107
	HTN(1)	-1.000	.092	119.359	1	.000	.368	.307	.440
	OBS(1)	.021	.118	.032	1	.858	1.021	.810	1.287
	FH(1)	.750	.092	66.184	1	.000	2.118	1.767	2.537
	hChol(1)	-.299	.084	12.515	1	.000	.742	.629	.875
	hTG(1)	-.610	.088	48.273	1	.000	.543	.458	.645
	lHDL(1)	-.269	.076	12.596	1	.000	.764	.658	.886
	hLDL(1)	.109	.111	.955	1	.328	1.115	.896	1.387
	Age	.031	.003	108.925	1	.000	1.032	1.026	1.038
	BMI	.046	.010	20.248	1	.000	1.047	1.026	1.068
	Constant	-2.546	.454	31.458	1	.000	.078		

a. Variable(s) entered on step 1: rs2259820D, MI, Sex, HTN, OBS, FH, hChol, hTG, lHDL, hLDL, Age, BMI.

rs2464196A(1)

Variables in the Equation

		B	S.E.	Wald	df	Sig.	Exp(B)	95% C.I.for EXP(B)	
								Lower	Upper
Step 1[a]	rs2464196A(1)	.114	.051	5.063	1	.024	1.121	1.015	1.237
	MI	.794	.058	185.720	1	.000	2.212	1.973	2.480
	Age	.035	.002	292.567	1	.000	1.036	1.032	1.040
	BMI	.043	.007	36.825	1	.000	1.044	1.029	1.058
	Sex(1)	-.084	.058	2.102	1	.147	.919	.821	1.030
	HTN(1)	-1.012	.064	252.507	1	.000	.364	.321	.412
	OBS(1)	.028	.082	.116	1	.733	1.028	.876	1.207
	hChol(1)	-.285	.058	23.799	1	.000	.752	.671	.843
	hTG(1)	-.573	.061	89.148	1	.000	.564	.501	.635
	lHDL(1)	-.292	.053	30.581	1	.000	.747	.674	.828
	hLDL(1)	.139	.077	3.227	1	.072	1.149	.987	1.336
	Constant	-2.655	.291	83.539	1	.000	.070		

a. Variable(s) entered on step 1: rs2464196A, MI, Age, BMI, Sex, HTN, OBS, hChol, hTG, lHDL, hLDL.

Variables in the Equation

		B	S.E.	Wald	df	Sig.	Exp(B)	95% C.I.for EXP(B)	
								Lower	Upper
Step 1[a]	rs2464196R	-.142	.085	2.798	1	.094	.868	.735	1.025
	MI(1)	-.750	.083	80.946	1	.000	.472	.401	.556
	Sex(1)	-.051	.083	.380	1	.538	.950	.808	1.117
	HTN(1)	-.987	.091	118.688	1	.000	.373	.312	.445
	OBS(1)	.024	.117	.043	1	.835	1.025	.815	1.288
	FH(1)	.766	.092	69.537	1	.000	2.151	1.796	2.575
	hChol(1)	-.309	.084	13.586	1	.000	.734	.623	.865
	hTG(1)	-.602	.087	47.830	1	.000	.548	.462	.650
	lHDL(1)	-.280	.075	13.814	1	.000	.756	.652	.876
	hLDL(1)	.119	.111	1.157	1	.282	1.126	.907	1.399
	Age	.032	.003	114.286	1	.000	1.032	1.026	1.038
	BMI	.047	.010	21.720	1	.000	1.048	1.028	1.069
	Constant	-2.135	.436	24.018	1	.000	.118		

a. Variable(s) entered on step 1: rs2464196R, MI, Sex, HTN, OBS, FH, hChol, hTG, lHDL, hLDL, Age, BMI.

Variables in the Equation

		B	S.E.	Wald	df	Sig.	Exp(B)	95% C.I.for EXP(B)	
								Lower	Upper
Step 1[a]	rs2464196D	-.136	.079	2.932	1	.087	.873	.748	1.020
	MI(1)	-.745	.083	79.879	1	.000	.475	.403	.559
	Sex(1)	-.051	.083	.378	1	.538	.950	.808	1.118
	HTN(1)	-.990	.091	119.137	1	.000	.372	.311	.444
	OBS(1)	.027	.117	.053	1	.819	1.027	.817	1.292
	FH(1)	.770	.092	70.404	1	.000	2.161	1.805	2.587
	hChol(1)	-.308	.084	13.490	1	.000	.735	.624	.866
	hTG(1)	-.604	.087	48.193	1	.000	.547	.461	.648
	lHDL(1)	-.279	.075	13.701	1	.000	.757	.653	.877
	hLDL(1)	.120	.111	1.172	1	.279	1.127	.907	1.400
	Age	.032	.003	114.707	1	.000	1.032	1.026	1.038
	BMI	.047	.010	21.931	1	.000	1.048	1.028	1.069
	Constant	-2.096	.441	22.595	1	.000	.123		

a. Variable(s) entered on step 1: rs2464196D, MI, Sex, HTN, OBS, FH, hChol, hTG, lHDL, hLDL, Age, BMI.

Variables in the Equation

		B	S.E.	Wald	df	Sig.	Exp(B)	95% C.I.for EXP(B)	
								Lower	Upper
Step 1[a]	rs2259816R	.115	.077	2.225	1	.136	1.122	.964	1.306
	MI(1)	-.706	.085	68.457	1	.000	.494	.418	.584
	Sex(1)	-.037	.084	.196	1	.658	.963	.816	1.137
	HTN(1)	-.995	.092	117.220	1	.000	.370	.309	.443
	OBS(1)	.005	.119	.002	1	.968	1.005	.795	1.269
	FH(1)	.746	.093	63.763	1	.000	2.108	1.755	2.531
	hChol(1)	-.282	.085	10.982	1	.001	.754	.639	.891
	hTG(1)	-.596	.088	45.281	1	.000	.551	.463	.656
	lHDL(1)	-.265	.077	11.968	1	.001	.767	.660	.891
	hLDL(1)	.154	.113	1.870	1	.171	1.167	.935	1.456
	Age	.032	.003	109.393	1	.000	1.032	1.026	1.038
	BMI	.047	.010	20.892	1	.000	1.048	1.027	1.069
	Constant	-2.527	.448	31.855	1	.000	.080		

a. Variable(s) entered on step 1: rs2259816R, MI, Sex, HTN, OBS, FH, hChol, hTG, lHDL, hLDL, Age, BMI.

Variables in the Equation

		B	S.E.	Wald	df	Sig.	Exp(B)	95% C.I.for EXP(B)	
								Lower	Upper
Step 1[a]	rs2259816D	.069	.093	.557	1	.455	1.072	.894	1.285
	MI(1)	-.708	.085	68.888	1	.000	.493	.417	.582
	Sex(1)	-.036	.084	.187	1	.665	.964	.817	1.138
	HTN(1)	-.993	.092	116.833	1	.000	.370	.309	.443
	OBS(1)	.001	.119	.000	1	.992	1.001	.793	1.265
	FH(1)	.747	.093	63.977	1	.000	2.110	1.757	2.533
	hChol(1)	-.284	.085	11.156	1	.001	.753	.637	.889
	hTG(1)	-.592	.088	44.780	1	.000	.553	.465	.658
	lHDL(1)	-.266	.077	12.094	1	.001	.766	.659	.890
	hLDL(1)	.152	.113	1.816	1	.178	1.164	.933	1.452
	Age	.032	.003	108.757	1	.000	1.032	1.026	1.038
	BMI	.047	.010	20.639	1	.000	1.048	1.027	1.069
	Constant	-2.481	.464	28.631	1	.000	.084		

a. Variable(s) entered on step 1: rs2259816D, MI, Sex, HTN, OBS, FH, hChol, hTG, lHDL, hLDL, Age, BMI.

rs2259816A

Variables in the Equation

		B	S.E.	Wald	df	Sig.	Exp(B)	95% C.I.for EXP(B)	
								Lower	Upper
Step 1[a]	rs2259816A	.082	.052	2.463	1	.117	1.085	.980	1.201
	MI	.755	.060	160.491	1	.000	2.128	1.893	2.392
	Age	.035	.002	277.990	1	.000	1.036	1.032	1.040
	BMI	.042	.007	34.288	1	.000	1.043	1.028	1.058
	Sex(1)	-.069	.059	1.365	1	.243	.933	.831	1.048
	HTN(1)	-1.018	.065	248.396	1	.000	.361	.318	.410
	OBS(1)	.000	.083	.000	1	.998	1.000	.849	1.177
	hChol(1)	-.260	.059	19.285	1	.000	.771	.686	.866
	hTG(1)	-.571	.062	85.461	1	.000	.565	.501	.638
	lHDL(1)	-.278	.054	26.854	1	.000	.757	.682	.841
	hLDL(1)	.180	.079	5.199	1	.023	1.197	1.026	1.396
	Constant	-2.729	.307	78.978	1	.000	.065		

a. Variable(s) entered on step 1: rs2259816A, MI, Age, BMI, Sex, HTN, OBS, hChol, hTG, lHDL, hLDL.

Variables in the Equation

		B	S.E.	Wald	df	Sig.	Exp(B)	95% C.I.for EXP(B)	
								Lower	Upper
Step 1[a]	rs2259816R	.115	.077	2.225	1	.136	1.122	.964	1.306
	MI(1)	-.706	.085	68.457	1	.000	.494	.418	.584
	Sex(1)	-.037	.084	.196	1	.658	.963	.816	1.137
	HTN(1)	-.995	.092	117.220	1	.000	.370	.309	.443
	OBS(1)	.005	.119	.002	1	.968	1.005	.795	1.269
	FH(1)	.746	.093	63.763	1	.000	2.108	1.755	2.531
	hChol(1)	-.282	.085	10.982	1	.001	.754	.639	.891
	hTG(1)	-.596	.088	45.281	1	.000	.551	.463	.656
	lHDL(1)	-.265	.077	11.968	1	.001	.767	.660	.891
	hLDL(1)	.154	.113	1.870	1	.171	1.167	.935	1.456
	Age	.032	.003	109.393	1	.000	1.032	1.026	1.038
	BMI	.047	.010	20.892	1	.000	1.048	1.027	1.069
	Constant	-2.527	.448	31.855	1	.000	.080		

a. Variable(s) entered on step 1: rs2259816R, MI, Sex, HTN, OBS, FH, hChol, hTG, lHDL, hLDL, Age, BMI.

Variables in the Equation

		B	S.E.	Wald	df	Sig.	Exp(B)	95% C.I.for EXP(B)	
								Lower	Upper
Step 1[a]	rs2259816D	.069	.093	.557	1	.455	1.072	.894	1.285
	MI(1)	-.708	.085	68.888	1	.000	.493	.417	.582
	Sex(1)	-.036	.084	.187	1	.665	.964	.817	1.138
	HTN(1)	-.993	.092	116.833	1	.000	.370	.309	.443
	OBS(1)	.001	.119	.000	1	.992	1.001	.793	1.265
	FH(1)	.747	.093	63.977	1	.000	2.110	1.757	2.533
	hChol(1)	-.284	.085	11.156	1	.001	.753	.637	.889
	hTG(1)	-.592	.088	44.780	1	.000	.553	.465	.658
	lHDL(1)	-.266	.077	12.094	1	.001	.766	.659	.890
	hLDL(1)	.152	.113	1.816	1	.178	1.164	.933	1.452
	Age	.032	.003	108.757	1	.000	1.032	1.026	1.038
	BMI	.047	.010	20.639	1	.000	1.048	1.027	1.069
	Constant	-2.481	.464	28.631	1	.000	.084		

a. Variable(s) entered on step 1: rs2259816D, MI, Sex, HTN, OBS, FH, hChol, hTG, lHDL, hLDL, Age, BMI.

rs1169310A

Variables in the Equation

		B	S.E.	Wald	df	Sig.	Exp(B)	95% C.I.for EXP(B)	
								Lower	Upper
Step 1[a]	rs1169310A	-.104	.051	4.174	1	.041	.901	.816	.996
	MI	.780	.058	180.449	1	.000	2.182	1.947	2.445
	Age	.035	.002	296.113	1	.000	1.036	1.032	1.040
	BMI	.043	.007	37.822	1	.000	1.044	1.030	1.058
	Sex(1)	-.075	.058	1.684	1	.194	.928	.828	1.039
	HTN(1)	-1.008	.064	251.743	1	.000	.365	.322	.413
	OBS(1)	.038	.081	.217	1	.642	1.039	.886	1.218
	hChol(1)	-.271	.058	21.723	1	.000	.763	.681	.855
	hTG(1)	-.584	.060	93.539	1	.000	.558	.496	.628
	lHDL(1)	-.289	.052	30.215	1	.000	.749	.676	.831
	hLDL(1)	.142	.077	3.391	1	.066	1.152	.991	1.339
	Constant	-2.477	.295	70.276	1	.000	.084		

a. Variable(s) entered on step 1: rs1169310A, MI, Age, BMI, Sex, HTN, OBS, hChol, hTG, lHDL, hLDL.

Variables in the Equation

		B	S.E.	Wald	df	Sig.	Exp(B)	95% C.I.for EXP(B)	
								Lower	Upper
Step 1[a]	rs1169310R	-.118	.092	1.643	1	.200	.889	.742	1.064
	MI(1)	-.736	.083	78.495	1	.000	.479	.407	.564
	Sex(1)	-.042	.082	.262	1	.609	.959	.816	1.127
	HTN(1)	-.984	.090	118.511	1	.000	.374	.313	.446
	OBS(1)	.031	.116	.073	1	.787	1.032	.821	1.296
	FH(1)	.780	.092	72.566	1	.000	2.181	1.823	2.609
	hChol(1)	-.296	.083	12.578	1	.000	.744	.632	.876
	hTG(1)	-.611	.087	49.845	1	.000	.543	.458	.643
	lHDL(1)	-.278	.075	13.777	1	.000	.757	.654	.877
	hLDL(1)	.118	.110	1.152	1	.283	1.126	.907	1.397
	Age	.032	.003	114.919	1	.000	1.032	1.026	1.038
	BMI	.047	.010	22.165	1	.000	1.048	1.028	1.069
	Constant	-2.211	.435	25.881	1	.000	.110		

a. Variable(s) entered on step 1: rs1169310R, MI, Sex, HTN, OBS, FH, hChol, hTG, lHDL, hLDL, Age, BMI.

Variables in the Equation

		B	S.E.	Wald	df	Sig.	Exp(B)	95% C.I.for EXP(B)	
								Lower	Upper
Step 1[a]	rs1169310D	-.135	.075	3.212	1	.073	.874	.754	1.013
	MI(1)	-.734	.083	77.949	1	.000	.480	.408	.565
	Sex(1)	-.043	.082	.266	1	.606	.958	.815	1.127
	HTN(1)	-.987	.090	119.010	1	.000	.373	.312	.445
	OBS(1)	.036	.116	.095	1	.758	1.036	.825	1.302
	FH(1)	.781	.092	72.781	1	.000	2.184	1.825	2.613
	hChol(1)	-.293	.083	12.358	1	.000	.746	.633	.878
	hTG(1)	-.615	.087	50.395	1	.000	.541	.456	.641
	lHDL(1)	-.277	.075	13.591	1	.000	.758	.655	.878
	hLDL(1)	.121	.110	1.203	1	.273	1.129	.909	1.401
	Age	.032	.003	115.520	1	.000	1.032	1.026	1.038
	BMI	.047	.010	22.530	1	.000	1.049	1.028	1.069
	Constant	-2.151	.435	24.418	1	.000	.116		

a. Variable(s) entered on step 1: rs1169310D, MI, Sex, HTN, OBS, FH, hChol, hTG, lHDL, hLDL, Age, BMI.

Variables in the Equation

		B	S.E.	Wald	df	Sig.	Exp(B)	95% C.I.for EXP(B)	
								Lower	Upper
Step 1[a]	rs1169313A	-.094	.051	3.375	1	.066	.910	.823	1.006
	MI	.782	.059	177.347	1	.000	2.185	1.947	2.451
	Age	.035	.002	288.783	1	.000	1.036	1.032	1.040
	BMI	.043	.007	36.351	1	.000	1.044	1.029	1.058
	Sex(1)	-.095	.058	2.674	1	.102	.909	.811	1.019
	HTN(1)	-1.042	.064	262.722	1	.000	.353	.311	.400
	OBS(1)	.042	.082	.263	1	.608	1.043	.888	1.225
	hChol(1)	-.269	.059	21.046	1	.000	.764	.681	.857
	hTG(1)	-.574	.061	88.681	1	.000	.563	.500	.635
	lHDL(1)	-.291	.053	30.145	1	.000	.748	.674	.829
	hLDL(1)	.109	.077	1.973	1	.160	1.115	.958	1.297
	Constant	-2.442	.299	66.764	1	.000	.087		

a. Variable(s) entered on step 1: rs1169313A, MI, Age, BMI, Sex, HTN, OBS, hChol, hTG, lHDL, hLDL.

Variables in the Equation

		B	S.E.	Wald	df	Sig.	Exp(B)	95% C.I.for EXP(B)	
								Lower	Upper
Step 1[a]	rs1169313R	-.125	.093	1.811	1	.178	.883	.736	1.059
	MI(1)	-.740	.084	77.833	1	.000	.477	.405	.562
	Sex(1)	-.063	.083	.584	1	.445	.939	.798	1.104
	HTN(1)	-1.020	.091	124.286	1	.000	.361	.301	.431
	OBS(1)	.035	.118	.088	1	.766	1.036	.822	1.304
	FH(1)	.763	.092	68.374	1	.000	2.144	1.790	2.569
	hChol(1)	-.292	.084	12.103	1	.001	.746	.633	.880
	hTG(1)	-.598	.087	46.878	1	.000	.550	.463	.652
	lHDL(1)	-.281	.076	13.849	1	.000	.755	.651	.875
	hLDL(1)	.087	.111	.611	1	.434	1.090	.878	1.355
	Age	.032	.003	112.851	1	.000	1.032	1.026	1.038
	BMI	.047	.010	21.146	1	.000	1.048	1.027	1.069
	Constant	-2.140	.440	23.667	1	.000	.118		

a. Variable(s) entered on step 1: rs1169313R, MI, Sex, HTN, OBS, FH, hChol, hTG, lHDL, hLDL, Age, BMI.

Variables in the Equation

		B	S.E.	Wald	df	Sig.	Exp(B)	95% C.I.for EXP(B)	
								Lower	Upper
Step 1[a]	rs1169313D	-.115	.076	2.295	1	.130	.891	.768	1.034
	MI(1)	-.738	.084	77.239	1	.000	.478	.406	.564
	Sex(1)	-.064	.083	.590	1	.442	.938	.797	1.104
	HTN(1)	-1.020	.092	124.373	1	.000	.360	.301	.431
	OBS(1)	.039	.118	.109	1	.742	1.040	.826	1.309
	FH(1)	.764	.092	68.629	1	.000	2.148	1.792	2.573
	hChol(1)	-.290	.084	11.908	1	.001	.748	.634	.882
	hTG(1)	-.602	.087	47.395	1	.000	.548	.461	.650
	lHDL(1)	-.280	.076	13.683	1	.000	.756	.652	.877
	hLDL(1)	.089	.111	.641	1	.423	1.093	.879	1.358
	Age	.032	.003	113.574	1	.000	1.032	1.026	1.038
	BMI	.047	.010	21.464	1	.000	1.048	1.027	1.069
	Constant	-2.119	.440	23.207	1	.000	.120		

a. Variable(s) entered on step 1: rs1169313D, MI, Sex, HTN, OBS, FH, hChol, hTG, lHDL, hLDL, Age, BMI.

HNF1a vs Hypertension

Variables in the Equation

		B	S.E.	Wald	df	Sig.	Exp(B)	95% C.I.for EXP(B)	
								Lower	Upper
Step 1[a]	MI(1)	-.232	.069	11.352	1	.001	.793	.693	.908
	Age	.040	.002	309.913	1	.000	1.040	1.036	1.045
	BMI	.034	.008	18.584	1	.000	1.035	1.019	1.051
	Sex(1)	-.223	.067	11.228	1	.001	.800	.702	.912
	DM(1)	-.988	.064	236.952	1	.000	.372	.328	.422
	hChol(1)	-.412	.073	31.772	1	.000	.662	.574	.764
	hTG	-.079	.071	1.221	1	.269	.924	.804	1.063
	lHDL(1)	-.040	.062	.425	1	.515	.961	.852	1.084
	hLDL(1)	.159	.091	3.007	1	.083	1.172	.980	1.402
	OBS(1)	.002	.095	.000	1	.986	1.002	.831	1.207
	rs2393791A	.127	.059	4.580	1	.032	1.136	1.011	1.276
	Constant	-1.100	.335	10.781	1	.001	.333		

a. Variable(s) entered on step 1: MI, Age, BMI, Sex, DM, hChol, hTG, lHDL, hLDL, OBS, rs2393791A.

Variables in the Equation

		B	S.E.	Wald	df	Sig.	Exp(B)	95% C.I.for EXP(B)	
								Lower	Upper
Step 1[a]	rs2393791R(1)	-.164	.111	2.195	1	.138	.848	.683	1.055
	MI(1)	-.231	.097	5.622	1	.018	.794	.656	.961
	Sex(1)	-.219	.094	5.414	1	.020	.803	.668	.966
	DM	.973	.092	113.022	1	.000	2.646	2.212	3.166
	OBS(1)	.002	.134	.000	1	.986	1.002	.770	1.305
	FH(1)	.101	.097	1.079	1	.299	1.106	.914	1.338
	hChol(1)	-.414	.103	16.048	1	.000	.661	.540	.809
	hTG(1)	.073	.101	.529	1	.467	1.076	.883	1.311
	lHDL(1)	-.039	.087	.197	1	.657	.962	.811	1.141
	hLDL(1)	.156	.129	1.458	1	.227	1.169	.907	1.507
	Age	.039	.003	150.835	1	.000	1.040	1.034	1.047
	BMI	.035	.011	9.638	1	.002	1.035	1.013	1.058
	Constant	-1.926	.469	16.872	1	.000	.146		

a. Variable(s) entered on step 1: rs2393791R, MI, Sex, DM, OBS, FH, hChol, hTG, lHDL, hLDL, Age, BMI.

Variables in the Equation

		B	S.E.	Wald	df	Sig.	Exp(B)	95% C.I.for EXP(B)	
								Lower	Upper
Step 1[a]	rs2393791D	.160	.084	3.618	1	.057	1.174	.995	1.384
	MI(1)	-.233	.097	5.708	1	.017	.792	.655	.959
	Sex(1)	-.216	.094	5.242	1	.022	.806	.670	.969
	DM	.977	.092	113.738	1	.000	2.657	2.220	3.179
	OBS(1)	.001	.134	.000	1	.993	1.001	.769	1.303
	FH(1)	.100	.097	1.057	1	.304	1.105	.913	1.337
	hChol(1)	-.417	.103	16.245	1	.000	.659	.538	.807
	hTG(1)	.078	.101	.598	1	.439	1.081	.887	1.317
	lHDL(1)	-.038	.087	.194	1	.660	.962	.811	1.141
	hLDL(1)	.152	.130	1.370	1	.242	1.164	.903	1.500
	Age	.039	.003	150.230	1	.000	1.040	1.034	1.047
	BMI	.035	.011	9.654	1	.002	1.035	1.013	1.058
	Constant	-2.317	.476	23.672	1	.000	.099		

a. Variable(s) entered on step 1: rs2393791D, MI, Sex, DM, OBS, FH, hChol, hTG, lHDL, hLDL, Age, BMI.

Variables in the Equation

		B	S.E.	Wald	df	Sig.	Exp(B)	95% C.I.for EXP(B)	
								Lower	Upper
Step 1[a]	rs7310409A	.148	.059	6.163	1	.013	1.159	1.032	1.302
	MI(1)	-.248	.069	13.136	1	.000	.780	.682	.892
	Age	.040	.002	313.815	1	.000	1.041	1.036	1.045
	BMI	.034	.008	18.488	1	.000	1.034	1.019	1.051
	Sex(1)	-.224	.066	11.398	1	.001	.799	.702	.910
	DM(1)	-.984	.064	236.637	1	.000	.374	.330	.424
	hChol(1)	-.415	.073	32.323	1	.000	.661	.573	.762
	hTG	-.068	.071	.911	1	.340	.935	.813	1.074
	lHDL(1)	-.031	.061	.260	1	.610	.969	.860	1.093
	hLDL(1)	.161	.091	3.099	1	.078	1.174	.982	1.404
	OBS(1)	-.001	.095	.000	1	.995	.999	.830	1.203
	Constant	-1.136	.334	11.565	1	.001	.321		

a. Variable(s) entered on step 1: rs7310409A, MI, Age, BMI, Sex, DM, hChol, hTG, lHDL, hLDL, OBS.

Variables in the Equation

		B	S.E.	Wald	df	Sig.	Exp(B)	95% C.I.for EXP(B)	
								Lower	Upper
Step 1[a]	rs7310409R	.176	.112	2.460	1	.117	1.193	.957	1.487
	MI(1)	-.248	.097	6.566	1	.010	.780	.645	.943
	Sex(1)	-.221	.094	5.516	1	.019	.802	.667	.964
	DM	.968	.091	112.556	1	.000	2.633	2.202	3.148
	OBS(1)	.002	.134	.000	1	.988	1.002	.771	1.303
	FH(1)	.102	.097	1.114	1	.291	1.108	.916	1.339
	hChol(1)	-.417	.103	16.311	1	.000	.659	.538	.807
	hTG(1)	.062	.100	.380	1	.537	1.064	.874	1.295
	lHDL(1)	-.029	.087	.112	1	.738	.971	.820	1.151
	hLDL(1)	.159	.129	1.514	1	.218	1.172	.910	1.509
	Age	.039	.003	152.329	1	.000	1.040	1.034	1.047
	BMI	.035	.011	9.674	1	.002	1.035	1.013	1.058
	Constant	-2.263	.474	22.773	1	.000	.104		

a. Variable(s) entered on step 1: rs7310409R, MI, Sex, DM, OBS, FH, hChol, hTG, lHDL, hLDL, Age, BMI.

Variables in the Equation

		B	S.E.	Wald	df	Sig.	Exp(B)	95% C.I.for EXP(B)	
								Lower	Upper
Step 1[a]	rs7310409D	.196	.084	5.454	1	.020	1.216	1.032	1.433
	MI(1)	-.247	.097	6.525	1	.011	.781	.646	.944
	Sex(1)	-.217	.094	5.323	1	.021	.805	.670	.968
	DM	.974	.091	113.652	1	.000	2.648	2.214	3.167
	OBS(1)	-.004	.134	.001	1	.977	.996	.766	1.295
	FH(1)	.101	.097	1.079	1	.299	1.106	.915	1.337
	hChol(1)	-.421	.103	16.622	1	.000	.656	.536	.804
	hTG(1)	.068	.100	.451	1	.502	1.070	.879	1.303
	lHDL(1)	-.031	.087	.128	1	.721	.969	.818	1.149
	hLDL(1)	.153	.129	1.408	1	.235	1.166	.905	1.502
	Age	.040	.003	152.412	1	.000	1.040	1.034	1.047
	BMI	.034	.011	9.491	1	.002	1.035	1.013	1.058
	Constant	-2.361	.474	24.800	1	.000	.094		

a. Variable(s) entered on step 1: rs7310409D, MI, Sex, DM, OBS, FH, hChol, hTG, lHDL, hLDL, Age, BMI.

Variables in the Equation

		B	S.E.	Wald	df	Sig.	Exp(B)	95% C.I.for EXP(B)	
								Lower	Upper
Step 1[a]	rs2259820R	-.204	.089	5.184	1	.023	.816	.685	.972
	MI(1)	-.232	.098	5.557	1	.018	.793	.654	.962
	Sex(1)	-.206	.095	4.716	1	.030	.814	.675	.980
	DM	.992	.093	114.683	1	.000	2.697	2.249	3.233
	OBS(1)	.002	.136	.000	1	.990	1.002	.767	1.308
	FH(1)	.110	.098	1.264	1	.261	1.116	.921	1.352
	hChol(1)	-.405	.105	14.906	1	.000	.667	.543	.819
	hTG(1)	.068	.102	.442	1	.506	1.070	.876	1.307
	lHDL(1)	-.037	.088	.178	1	.673	.964	.811	1.145
	hLDL(1)	.134	.131	1.038	1	.308	1.143	.884	1.479
	Age	.040	.003	148.382	1	.000	1.040	1.034	1.047
	BMI	.035	.011	9.417	1	.002	1.035	1.013	1.059
	Constant	-1.819	.483	14.198	1	.000	.162		

a. Variable(s) entered on step 1: rs2259820R, MI, Sex, DM, OBS, FH, hChol, hTG, lHDL, hLDL, Age, BMI.

rs2259820A

Variables in the Equation

		B	S.E.	Wald	df	Sig.	Exp(B)	95% C.I.for EXP(B)	
								Lower	Upper
Step 1[a]	rs2259820A	-.149	.059	6.464	1	.011	.861	.768	.966
	MI(1)	-.228	.070	10.721	1	.001	.796	.695	.913
	Age	.040	.002	306.723	1	.000	1.041	1.036	1.045
	BMI	.034	.008	18.303	1	.000	1.035	1.019	1.051
	Sex(1)	-.213	.067	10.039	1	.002	.808	.709	.922
	DM(1)	-1.005	.065	240.056	1	.000	.366	.322	.416
	hChol(1)	-.399	.074	29.155	1	.000	.671	.580	.775
	hTG	-.068	.072	.882	1	.348	.935	.812	1.076
	lHDL(1)	-.039	.062	.388	1	.533	.962	.852	1.087
	hLDL(1)	.143	.093	2.387	1	.122	1.154	.962	1.384
	OBS(1)	.006	.096	.004	1	.949	1.006	.834	1.215
	Constant	-.721	.346	4.353	1	.037	.486		

a. Variable(s) entered on step 1: rs2259820A, MI, Age, BMI, Sex, DM, hChol, hTG, lHDL, hLDL, OBS.

Variables in the Equation

		B	S.E.	Wald	df	Sig.	Exp(B)	95% C.I.for EXP(B)	
								Lower	Upper
Step 1[a]	rs2259820R	-.204	.089	5.184	1	.023	.816	.685	.972
	MI(1)	-.232	.098	5.557	1	.018	.793	.654	.962
	Sex(1)	-.206	.095	4.716	1	.030	.814	.675	.980
	DM	.992	.093	114.683	1	.000	2.697	2.249	3.233
	OBS(1)	.002	.136	.000	1	.990	1.002	.767	1.308
	FH(1)	.110	.098	1.264	1	.261	1.116	.921	1.352
	hChol(1)	-.405	.105	14.906	1	.000	.667	.543	.819
	hTG(1)	.068	.102	.442	1	.506	1.070	.876	1.307
	lHDL(1)	-.037	.088	.178	1	.673	.964	.811	1.145
	hLDL(1)	.134	.131	1.038	1	.308	1.143	.884	1.479
	Age	.040	.003	148.382	1	.000	1.040	1.034	1.047
	BMI	.035	.011	9.417	1	.002	1.035	1.013	1.059
	Constant	-1.819	.483	14.198	1	.000	.162		

a. Variable(s) entered on step 1: rs2259820R, MI, Sex, DM, OBS, FH, hChol, hTG, lHDL, hLDL, Age, BMI.

Variables in the Equation

		B	S.E.	Wald	df	Sig.	Exp(B)	95% C.I.for EXP(B)	
								Lower	Upper
Step 1[a]	rs2259820D	-.177	.099	3.232	1	.072	.837	.690	1.016
	MI(1)	-.224	.098	5.184	1	.023	.799	.659	.969
	Sex(1)	-.207	.095	4.761	1	.029	.813	.675	.979
	DM	.991	.093	114.670	1	.000	2.695	2.248	3.231
	OBS(1)	.009	.136	.004	1	.950	1.009	.773	1.316
	FH(1)	.116	.098	1.395	1	.238	1.123	.927	1.360
	hChol(1)	-.404	.105	14.876	1	.000	.668	.544	.820
	hTG(1)	.062	.102	.366	1	.545	1.064	.871	1.299
	lHDL(1)	-.036	.088	.173	1	.678	.964	.812	1.145
	hLDL(1)	.141	.131	1.151	1	.283	1.151	.890	1.489
	Age	.040	.003	148.822	1	.000	1.040	1.034	1.047
	BMI	.035	.011	9.562	1	.002	1.036	1.013	1.059
	Constant	-1.796	.497	13.028	1	.000	.166		

a. Variable(s) entered on step 1: rs2259820D, MI, Sex, DM, OBS, FH, hChol, hTG, lHDL, hLDL, Age, BMI.

rs2464196A vs HTN

Variables in the Equation

		B	S.E.	Wald	df	Sig.	Exp(B)	95% C.I.for EXP(B)	
								Lower	Upper
Step 1[a]	rs2464196A(1)	-.142	.058	6.008	1	.014	.867	.774	.972
	MI(1)	-.236	.069	11.733	1	.001	.790	.690	.904
	Age	.039	.002	305.466	1	.000	1.040	1.036	1.045
	BMI	.035	.008	19.492	1	.000	1.035	1.020	1.052
	Sex(1)	-.213	.066	10.253	1	.001	.808	.710	.921
	DM(1)	-.992	.064	238.793	1	.000	.371	.327	.421
	hChol(1)	-.414	.073	31.917	1	.000	.661	.573	.763
	hTG	-.078	.071	1.194	1	.274	.925	.805	1.064
	lHDL(1)	-.026	.061	.184	1	.668	.974	.864	1.099
	hLDL(1)	.148	.092	2.617	1	.106	1.160	.969	1.388
	OBS(1)	-.004	.095	.002	1	.964	.996	.827	1.199
	Constant	-.858	.329	6.795	1	.009	.424		

a. Variable(s) entered on step 1: rs2464196A, MI, Age, BMI, Sex, DM, hChol, hTG, lHDL, hLDL, OBS.

Variables in the Equation

		B	S.E.	Wald	df	Sig.	Exp(B)	95% C.I.for EXP(B)	
								Lower	Upper
Step 1[a]	rs2464196R	.134	.097	1.898	1	.168	1.144	.945	1.384
	MI(1)	-.232	.097	5.679	1	.017	.793	.655	.960
	Sex(1)	-.207	.094	4.842	1	.028	.813	.676	.978
	DM	.978	.092	113.944	1	.000	2.658	2.221	3.180
	OBS(1)	-.001	.134	.000	1	.993	.999	.768	1.299
	FH(1)	.106	.097	1.178	1	.278	1.111	.918	1.345
	hChol(1)	-.417	.104	16.239	1	.000	.659	.538	.807
	hTG(1)	.071	.101	.502	1	.478	1.074	.882	1.309
	lHDL(1)	-.024	.087	.075	1	.784	.977	.824	1.158
	hLDL(1)	.147	.130	1.276	1	.259	1.158	.898	1.493
	Age	.039	.003	148.271	1	.000	1.040	1.033	1.046
	BMI	.036	.011	10.218	1	.001	1.036	1.014	1.059
	Constant	-2.252	.473	22.656	1	.000	.105		

a. Variable(s) entered on step 1: rs2464196R, MI, Sex, DM, OBS, FH, hChol, hTG, lHDL, hLDL, Age, BMI.

Variables in the Equation

		B	S.E.	Wald	df	Sig.	Exp(B)	95% C.I.for EXP(B)	
								Lower	Upper
Step 1[a]	rs2464196D	.223	.088	6.386	1	.012	1.250	1.051	1.487
	MI(1)	-.241	.097	6.110	1	.013	.786	.649	.951
	Sex(1)	-.207	.094	4.835	1	.028	.813	.676	.978
	DM	.980	.092	114.350	1	.000	2.666	2.227	3.190
	OBS(1)	-.008	.134	.004	1	.950	.992	.762	1.291
	FH(1)	.102	.097	1.099	1	.294	1.107	.915	1.340
	hChol(1)	-.418	.104	16.219	1	.000	.659	.537	.807
	hTG(1)	.077	.101	.590	1	.443	1.081	.887	1.317
	lHDL(1)	-.025	.087	.081	1	.776	.976	.823	1.157
	hLDL(1)	.139	.130	1.152	1	.283	1.150	.891	1.483
	Age	.039	.003	147.962	1	.000	1.040	1.033	1.046
	BMI	.035	.011	9.968	1	.002	1.036	1.014	1.059
	Constant	-2.440	.480	25.860	1	.000	.087		

a. Variable(s) entered on step 1: rs2464196D, MI, Sex, DM, OBS, FH, hChol, hTG, lHDL, hLDL, Age, BMI.

rs2259816A

Variables in the Equation

		B	S.E.	Wald	df	Sig.	Exp(B)	95% C.I.for EXP(B)	
								Lower	Upper
Step 1[a]	rs2259816A	-.083	.060	1.926	1	.165	.920	.818	1.035
	MI(1)	-.268	.070	14.664	1	.000	.765	.666	.877
	Age	.041	.002	309.059	1	.000	1.041	1.037	1.046
	BMI	.040	.008	24.450	1	.000	1.041	1.024	1.058
	Sex(1)	-.237	.068	12.056	1	.001	.789	.690	.902
	DM(1)	-.996	.065	234.179	1	.000	.370	.325	.420
	hChol(1)	-.413	.075	30.711	1	.000	.661	.571	.766
	hTG	-.077	.073	1.121	1	.290	.926	.803	1.068
	lHDL(1)	-.041	.063	.426	1	.514	.960	.849	1.085
	hLDL(1)	.170	.094	3.285	1	.070	1.185	.986	1.424
	OBS(1)	.026	.097	.071	1	.790	1.026	.848	1.241
	Constant	-1.040	.350	8.845	1	.003	.353		

a. Variable(s) entered on step 1: rs2259816A, MI, Age, BMI, Sex, DM, hChol, hTG, lHDL, hLDL, OBS.

Variables in the Equation

		B	S.E.	Wald	df	Sig.	Exp(B)	95% C.I.for EXP(B)	
								Lower	Upper
Step 1[a]	rs2259816R	-.124	.088	1.994	1	.158	.884	.744	1.049
	MI(1)	-.269	.099	7.353	1	.007	.764	.629	.928
	Sex(1)	-.230	.097	5.669	1	.017	.794	.657	.960
	DM	.984	.093	112.277	1	.000	2.675	2.230	3.209
	OBS(1)	.024	.137	.031	1	.860	1.025	.783	1.341
	FH(1)	.106	.099	1.141	1	.286	1.112	.915	1.350
	hChol(1)	-.418	.106	15.684	1	.000	.658	.535	.810
	hTG(1)	.076	.103	.547	1	.459	1.079	.882	1.320
	lHDL(1)	-.039	.089	.190	1	.663	.962	.809	1.145
	hLDL(1)	.163	.133	1.511	1	.219	1.177	.908	1.526
	Age	.040	.003	149.500	1	.000	1.041	1.034	1.048
	BMI	.041	.012	12.601	1	.000	1.042	1.018	1.065
	Constant	-2.148	.488	19.369	1	.000	.117		

a. Variable(s) entered on step 1: rs2259816R, MI, Sex, DM, OBS, FH, hChol, hTG, lHDL, hLDL, Age, BMI.

Variables in the Equation

		B	S.E.	Wald	df	Sig.	Exp(B)	95% C.I.for EXP(B)	
								Lower	Upper
Step 1[a]	rs2259816D	-.084	.106	.621	1	.431	.920	.747	1.133
	MI(1)	-.267	.099	7.245	1	.007	.766	.631	.930
	Sex(1)	-.232	.097	5.759	1	.016	.793	.656	.958
	DM	.982	.093	111.938	1	.000	2.669	2.225	3.201
	OBS(1)	.029	.137	.045	1	.831	1.030	.787	1.348
	FH(1)	.104	.099	1.103	1	.294	1.110	.914	1.348
	hChol(1)	-.417	.106	15.572	1	.000	.659	.536	.811
	hTG(1)	.072	.103	.490	1	.484	1.075	.879	1.314
	lHDL(1)	-.038	.089	.189	1	.664	.962	.809	1.145
	hLDL(1)	.166	.133	1.564	1	.211	1.180	.910	1.530
	Age	.040	.003	149.899	1	.000	1.041	1.034	1.048
	BMI	.041	.012	12.754	1	.000	1.042	1.019	1.066
	Constant	-2.174	.511	18.098	1	.000	.114		

a. Variable(s) entered on step 1: rs2259816D, MI, Sex, DM, OBS, FH, hChol, hTG, lHDL, hLDL, Age, BMI.

rs1169310A

Variables in the Equation

		B	S.E.	Wald	df	Sig.	Exp(B)	95% C.I.for EXP(B)	
								Lower	Upper
Step 1[a]	rs1169310A	.094	.059	2.571	1	.109	1.099	.979	1.233
	MI(1)	-.250	.069	13.253	1	.000	.779	.681	.891
	Age	.039	.002	308.398	1	.000	1.040	1.036	1.045
	BMI	.034	.008	19.077	1	.000	1.035	1.019	1.051
	Sex(1)	-.228	.066	11.803	1	.001	.796	.699	.907
	DM(1)	-.986	.064	237.453	1	.000	.373	.329	.423
	hChol(1)	-.416	.073	32.259	1	.000	.660	.572	.762
	hTG	-.077	.071	1.165	1	.280	.926	.806	1.065
	lHDL(1)	-.030	.061	.246	1	.620	.970	.860	1.094
	hLDL(1)	.166	.092	3.298	1	.069	1.181	.987	1.413
	OBS(1)	-.001	.095	.000	1	.995	.999	.830	1.203
	Constant	-1.055	.333	10.026	1	.002	.348		

a. Variable(s) entered on step 1: rs1169310A, MI, Age, BMI, Sex, DM, hChol, hTG, lHDL, hLDL, OBS.

Variables in the Equation

		B	S.E.	Wald	df	Sig.	Exp(B)	95% C.I.for EXP(B)	
								Lower	Upper
Step 1[a]	rs1169310R	.085	.106	.647	1	.421	1.089	.885	1.339
	MI(1)	-.248	.097	6.555	1	.010	.780	.645	.943
	Sex(1)	-.224	.094	5.657	1	.017	.799	.665	.961
	DM	.974	.091	113.606	1	.000	2.647	2.213	3.166
	OBS(1)	.002	.134	.000	1	.990	1.002	.770	1.303
	FH(1)	.090	.097	.863	1	.353	1.094	.905	1.324
	hChol(1)	-.418	.104	16.326	1	.000	.658	.537	.806
	hTG(1)	.071	.101	.503	1	.478	1.074	.882	1.308
	lHDL(1)	-.028	.087	.108	1	.743	.972	.820	1.152
	hLDL(1)	.164	.130	1.613	1	.204	1.179	.914	1.519
	Age	.039	.003	149.866	1	.000	1.040	1.033	1.046
	BMI	.035	.011	9.957	1	.002	1.036	1.013	1.059
	Constant	-2.155	.471	20.906	1	.000	.116		

a. Variable(s) entered on step 1: rs1169310R, MI, Sex, DM, OBS, FH, hChol, hTG, lHDL, hLDL, Age, BMI.

Variables in the Equation

		B	S.E.	Wald	df	Sig.	Exp(B)	95% C.I.for EXP(B)	
								Lower	Upper
Step 1[a]	rs1169310D	.145	.085	2.882	1	.090	1.156	.978	1.365
	MI(1)	-.251	.097	6.693	1	.010	.778	.643	.941
	Sex(1)	-.223	.094	5.610	1	.018	.800	.665	.962
	DM	.976	.091	114.046	1	.000	2.655	2.219	3.176
	OBS(1)	-.004	.134	.001	1	.975	.996	.766	1.295
	FH(1)	.090	.097	.866	1	.352	1.095	.905	1.324
	hChol(1)	-.420	.104	16.456	1	.000	.657	.536	.805
	hTG(1)	.076	.101	.578	1	.447	1.079	.886	1.315
	lHDL(1)	-.029	.087	.114	1	.735	.971	.819	1.151
	hLDL(1)	.159	.130	1.512	1	.219	1.173	.910	1.512
	Age	.039	.003	149.871	1	.000	1.040	1.033	1.046
	BMI	.035	.011	9.698	1	.002	1.035	1.013	1.058
	Constant	-2.274	.474	23.017	1	.000	.103		

a. Variable(s) entered on step 1: rs1169310D, MI, Sex, DM, OBS, FH, hChol, hTG, lHDL, hLDL, Age, BMI.

Variables in the Equation

		B	S.E.	Wald	df	Sig.	Exp(B)	95% C.I.for EXP(B)	
								Lower	Upper
Step 1[a]	rs1169313A	.108	.059	3.316	1	.069	1.114	.992	1.252
	MI(1)	-.228	.070	10.723	1	.001	.796	.695	.913
	Age	.039	.002	299.506	1	.000	1.040	1.036	1.045
	BMI	.036	.008	19.851	1	.000	1.036	1.020	1.053
	Sex(1)	-.217	.067	10.460	1	.001	.805	.706	.918
	DM(1)	-1.020	.065	247.780	1	.000	.360	.317	.409
	hChol(1)	-.414	.074	31.256	1	.000	.661	.571	.764
	hTG	-.078	.072	1.171	1	.279	.925	.804	1.065
	lHDL(1)	-.036	.062	.341	1	.559	.964	.854	1.089
	hLDL(1)	.183	.092	3.943	1	.047	1.201	1.002	1.438
	OBS(1)	.006	.096	.004	1	.948	1.006	.834	1.214
	Constant	-1.109	.338	10.802	1	.001	.330		

a. Variable(s) entered on step 1: rs1169313A, MI, Age, BMI, Sex, DM, hChol, hTG, lHDL, hLDL, OBS.

Variables in the Equation

		B	S.E.	Wald	df	Sig.	Exp(B)	95% C.I.for EXP(B)	
								Lower	Upper
Step 1[a]	rs1169313R	.144	.108	1.792	1	.181	1.155	.935	1.427
	MI(1)	-.227	.098	5.330	1	.021	.797	.657	.966
	Sex(1)	-.213	.095	5.023	1	.025	.808	.671	.974
	DM	1.009	.093	119.056	1	.000	2.744	2.289	3.289
	OBS(1)	.009	.136	.004	1	.949	1.009	.773	1.316
	FH(1)	.086	.098	.763	1	.383	1.090	.899	1.321
	hChol(1)	-.417	.105	15.779	1	.000	.659	.537	.810
	hTG(1)	.073	.102	.519	1	.471	1.076	.881	1.314
	lHDL(1)	-.035	.088	.156	1	.693	.966	.814	1.147
	hLDL(1)	.181	.130	1.920	1	.166	1.198	.928	1.546
	Age	.039	.003	145.819	1	.000	1.040	1.033	1.046
	BMI	.036	.011	10.263	1	.001	1.037	1.014	1.060
	Constant	-2.289	.478	22.949	1	.000	.101		

a. Variable(s) entered on step 1: rs1169313R, MI, Sex, DM, OBS, FH, hChol, hTG, lHDL, hLDL, Age, BMI.

Variables in the Equation

		B	S.E.	Wald	df	Sig.	Exp(B)	95% C.I.for EXP(B)	
								Lower	Upper
Step 1[a]	rs1169313D	.135	.086	2.459	1	.117	1.144	.967	1.355
	MI(1)	-.229	.098	5.446	1	.020	.795	.656	.964
	Sex(1)	-.212	.095	4.988	1	.026	.809	.672	.974
	DM	1.010	.093	119.225	1	.000	2.747	2.291	3.293
	OBS(1)	.004	.136	.001	1	.978	1.004	.769	1.310
	FH(1)	.085	.098	.749	1	.387	1.089	.898	1.320
	hChol(1)	-.419	.105	15.906	1	.000	.658	.536	.808
	hTG(1)	.078	.102	.583	1	.445	1.081	.885	1.320
	lHDL(1)	-.035	.088	.159	1	.690	.966	.813	1.147
	hLDL(1)	.177	.130	1.851	1	.174	1.194	.925	1.542
	Age	.039	.003	145.629	1	.000	1.040	1.033	1.046
	BMI	.036	.011	10.116	1	.001	1.037	1.014	1.060
	Constant	-2.327	.480	23.524	1	.000	.098		

a. Variable(s) entered on step 1: rs1169313D, MI, Sex, DM, OBS, FH, hChol, hTG, lHDL, hLDL, Age, BMI.

HNF1a vs Obesity

Variables in the Equation

		B	S.E.	Wald	df	Sig.	Exp(B)	95% C.I.for EXP(B)	
								Lower	Upper
Step 1[a]	rs2393791A	-.022	.163	.018	1	.892	.978	.710	1.347
	MI(1)	-.161	.209	.596	1	.440	.851	.565	1.282
	Age	-.004	.007	.328	1	.567	.996	.982	1.010
	BMI	3.347	.145	529.529	1	.000	28.403	21.359	37.771
	Sex(1)	.025	.178	.019	1	.890	1.025	.723	1.454
	HTN(1)	.002	.207	.000	1	.994	1.002	.667	1.504
	DM(1)	.252	.178	2.006	1	.157	1.287	.908	1.824
	hChol(1)	.030	.187	.026	1	.872	1.031	.715	1.486
	hTG	.185	.188	.968	1	.325	1.203	.832	1.740
	lHDL(1)	.126	.166	.574	1	.449	1.134	.819	1.569
	hLDL(1)	-.071	.244	.084	1	.772	.932	.578	1.503
	Constant	-100.199	4.402	518.091	1	.000	.000		

a. Variable(s) entered on step 1: rs2393791A, MI, Age, BMI, Sex, HTN, DM, hChol, hTG, lHDL, hLDL.

Variables in the Equation

		B	S.E.	Wald	df	Sig.	Exp(B)	95% C.I.for EXP(B)	
								Lower	Upper
Step 1[a]	rs2393791R(1)	-.010	.313	.001	1	.974	.990	.536	1.828
	Sex(1)	.030	.253	.014	1	.907	1.030	.628	1.691
	MI(1)	-.158	.296	.284	1	.594	.854	.478	1.525
	HTN(1)	.030	.298	.010	1	.919	1.031	.575	1.847
	DM	-.282	.256	1.217	1	.270	.754	.457	1.245
	FH(1)	.207	.292	.503	1	.478	1.230	.694	2.181
	hChol(1)	.026	.264	.009	1	.923	1.026	.611	1.723
	hTG(1)	-.193	.267	.521	1	.470	.825	.489	1.392
	lHDL(1)	.128	.235	.296	1	.586	1.136	.717	1.800
	hLDL(1)	-.079	.345	.053	1	.818	.924	.470	1.817
	Age	-.005	.010	.255	1	.613	.995	.976	1.015
	BMI	3.346	.205	265.085	1	.000	28.384	18.974	42.462
	Constant	-99.848	6.195	259.739	1	.000	.000		

a. Variable(s) entered on step 1: rs2393791R, Sex, MI, HTN, DM, FH, hChol, hTG, lHDL, hLDL, Age, BMI.

Variables in the Equation

		B	S.E.	Wald	df	Sig.	Exp(B)	95% C.I.for EXP(B)	
								Lower	Upper
Step 1[a]	rs2393791D	-.064	.229	.079	1	.778	.938	.599	1.468
	Sex(1)	.031	.252	.015	1	.901	1.032	.629	1.692
	MI(1)	-.158	.296	.285	1	.594	.854	.478	1.524
	HTN(1)	.035	.297	.014	1	.907	1.035	.579	1.852
	DM	-.286	.256	1.246	1	.264	.752	.455	1.241
	FH(1)	.214	.293	.534	1	.465	1.239	.698	2.199
	hChol(1)	.030	.264	.013	1	.908	1.031	.615	1.729
	hTG(1)	-.198	.268	.549	1	.459	.820	.485	1.386
	lHDL(1)	.129	.235	.304	1	.581	1.138	.719	1.803
	hLDL(1)	-.079	.345	.053	1	.818	.924	.470	1.817
	Age	-.005	.010	.229	1	.632	.995	.976	1.015
	BMI	3.346	.206	265.025	1	.000	28.397	18.980	42.485
	Constant	-99.787	6.194	259.500	1	.000	.000		

a. Variable(s) entered on step 1: rs2393791D, Sex, MI, HTN, DM, FH, hChol, hTG, lHDL, hLDL, Age, BMI.

rs7310409A

Variables in the Equation

		B	S.E.	Wald	df	Sig.	Exp(B)	95% C.I.for EXP(B)	
								Lower	Upper
Step 1[a]	rs7310409A	.066	.163	.166	1	.684	1.069	.776	1.471
	MI(1)	-.172	.208	.686	1	.408	.842	.560	1.266
	Age	-.005	.007	.489	1	.485	.995	.982	1.009
	BMI	3.368	.146	530.938	1	.000	29.014	21.787	38.639
	Sex(1)	.005	.178	.001	1	.976	1.005	.710	1.424
	HTN(1)	.016	.207	.006	1	.938	1.016	.678	1.524
	DM(1)	.286	.177	2.592	1	.107	1.331	.940	1.884
	hChol(1)	.013	.186	.005	1	.946	1.013	.703	1.458
	hTG	.174	.188	.854	1	.355	1.190	.823	1.719
	lHDL(1)	.106	.165	.410	1	.522	1.112	.804	1.537
	hLDL(1)	-.068	.243	.079	1	.779	.934	.580	1.504
	Constant	-100.876	4.429	518.661	1	.000	.000		

a. Variable(s) entered on step 1: rs7310409A, MI, Age, BMI, Sex, HTN, DM, hChol, hTG, lHDL, hLDL.

Variables in the Equation

		B	S.E.	Wald	df	Sig.	Exp(B)	95% C.I.for EXP(B)	
								Lower	Upper
Step 1[a]	rs7310409R	.197	.313	.395	1	.529	1.218	.659	2.249
	Sex(1)	.015	.252	.003	1	.953	1.015	.620	1.662
	MI(1)	-.164	.295	.308	1	.579	.849	.476	1.513
	HTN(1)	.055	.296	.035	1	.852	1.057	.591	1.889
	DM	-.320	.255	1.564	1	.211	.727	.440	1.199
	FH(1)	.168	.290	.337	1	.562	1.183	.671	2.086
	hChol(1)	.000	.263	.000	1	.999	1.000	.597	1.675
	hTG(1)	-.177	.267	.440	1	.507	.838	.497	1.413
	lHDL(1)	.106	.234	.205	1	.651	1.112	.703	1.758
	hLDL(1)	-.074	.344	.046	1	.830	.929	.473	1.824
	Age	-.006	.010	.312	1	.576	.994	.975	1.014
	BMI	3.368	.207	265.422	1	.000	29.035	19.361	43.543
	Constant	-100.651	6.248	259.524	1	.000	.000		

a. Variable(s) entered on step 1: rs7310409R, Sex, MI, HTN, DM, FH, hChol, hTG, lHDL, hLDL, Age, BMI.

Variables in the Equation

		B	S.E.	Wald	df	Sig.	Exp(B)	95% C.I.for EXP(B)	
								Lower	Upper
Step 1[a]	rs7310409D	.014	.229	.004	1	.953	1.014	.647	1.588
	Sex(1)	.008	.251	.001	1	.975	1.008	.616	1.650
	MI(1)	-.171	.295	.336	1	.562	.843	.473	1.502
	HTN(1)	.037	.296	.016	1	.899	1.038	.582	1.853
	DM	-.310	.255	1.476	1	.224	.734	.445	1.209
	FH(1)	.169	.290	.339	1	.561	1.184	.670	2.092
	hChol(1)	.015	.263	.003	1	.956	1.015	.606	1.700
	hTG(1)	-.183	.267	.473	1	.492	.832	.493	1.404
	lHDL(1)	.109	.234	.216	1	.642	1.115	.705	1.764
	hLDL(1)	-.075	.344	.047	1	.829	.928	.473	1.822
	Age	-.006	.010	.310	1	.578	.994	.975	1.014
	BMI	3.366	.206	265.737	1	.000	28.959	19.321	43.404
	Constant	-100.367	6.229	259.590	1	.000	.000		

a. Variable(s) entered on step 1: rs7310409D, Sex, MI, HTN, DM, FH, hChol, hTG, lHDL, hLDL, Age, BMI.

rs2259820A

Variables in the Equation

		B	S.E.	Wald	df	Sig.	Exp(B)	95% C.I.for EXP(B)	
								Lower	Upper
Step 1[a]	rs2259820A	-.013	.167	.006	1	.938	.987	.712	1.369
	MI(1)	-.097	.220	.195	1	.659	.907	.589	1.397
	Age	-.002	.007	.098	1	.754	.998	.983	1.012
	BMI	3.689	.169	479.289	1	.000	40.018	28.762	55.681
	Sex(1)	-.011	.188	.003	1	.953	.989	.684	1.430
	HTN(1)	.067	.218	.093	1	.760	1.069	.698	1.637
	DM(1)	.253	.189	1.793	1	.181	1.288	.889	1.864
	hChol(1)	.030	.198	.023	1	.879	1.031	.700	1.518
	hTG	.213	.199	1.145	1	.285	1.237	.838	1.826
	lHDL(1)	.166	.175	.901	1	.343	1.180	.838	1.662
	hLDL(1)	-.066	.257	.067	1	.796	.936	.565	1.549
	Constant	-110.614	5.101	470.219	1	.000	.000		

a. Variable(s) entered on step 1: rs2259820A, MI, Age, BMI, Sex, HTN, DM, hChol, hTG, lHDL, hLDL.

Variables in the Equation

		B	S.E.	Wald	df	Sig.	Exp(B)	95% C.I.for EXP(B)	
								Lower	Upper
Step 1[a]	rs2259820R	.064	.251	.065	1	.799	1.066	.651	1.745
	Sex(1)	-.005	.266	.000	1	.986	.995	.590	1.677
	MI(1)	-.091	.312	.084	1	.772	.913	.495	1.684
	HTN(1)	.092	.312	.086	1	.769	1.096	.595	2.019
	DM	-.274	.272	1.020	1	.313	.760	.447	1.294
	FH(1)	.140	.309	.205	1	.650	1.150	.628	2.108
	hChol(1)	.030	.279	.012	1	.913	1.031	.596	1.783
	hTG(1)	-.225	.282	.634	1	.426	.799	.459	1.389
	lHDL(1)	.170	.247	.474	1	.491	1.186	.730	1.925
	hLDL(1)	-.066	.364	.033	1	.856	.936	.459	1.909
	Age	-.003	.011	.061	1	.805	.997	.977	1.018
	BMI	3.687	.238	239.802	1	.000	39.907	25.027	63.633
	Constant	-110.257	7.184	235.521	1	.000	.000		

a. Variable(s) entered on step 1: rs2259820R, Sex, MI, HTN, DM, FH, hChol, hTG, lHDL, hLDL, Age, BMI.

Variables in the Equation

		B	S.E.	Wald	df	Sig.	Exp(B)	95% C.I.for EXP(B)	
								Lower	Upper
Step 1[a]	rs2259820D	-.108	.281	.147	1	.701	.898	.518	1.556
	Sex(1)	-.005	.266	.000	1	.985	.995	.591	1.676
	MI(1)	-.095	.311	.094	1	.760	.909	.494	1.674
	HTN(1)	.089	.311	.082	1	.774	1.093	.594	2.012
	DM	-.278	.272	1.049	1	.306	.757	.445	1.289
	FH(1)	.134	.308	.190	1	.663	1.144	.625	2.094
	hChol(1)	.026	.279	.008	1	.927	1.026	.593	1.774
	hTG(1)	-.217	.282	.592	1	.442	.805	.463	1.399
	lHDL(1)	.161	.248	.425	1	.514	1.175	.723	1.909
	hLDL(1)	-.076	.364	.043	1	.835	.927	.454	1.892
	Age	-.003	.010	.085	1	.771	.997	.977	1.018
	BMI	3.690	.238	239.656	1	.000	40.043	25.098	63.888
	Constant	-110.040	7.179	234.961	1	.000	.000		

a. Variable(s) entered on step 1: rs2259820D, Sex, MI, HTN, DM, FH, hChol, hTG, lHDL, hLDL, Age, BMI.

Variables in the Equation

		B	S.E.	Wald	df	Sig.	Exp(B)	95% C.I.for EXP(B)	
								Lower	Upper
Step 1[a]	rs2464196A(1)	-.050	.159	.099	1	.753	.951	.697	1.299
	MI(1)	-.136	.209	.425	1	.515	.873	.580	1.314
	Age	-.005	.007	.455	1	.500	.995	.982	1.009
	BMI	3.347	.146	528.989	1	.000	28.431	21.375	37.817
	Sex(1)	.005	.178	.001	1	.980	1.005	.708	1.425
	HTN(1)	.005	.207	.000	1	.982	1.005	.670	1.506
	DM(1)	.227	.179	1.608	1	.205	1.255	.883	1.784
	hChol(1)	.078	.187	.174	1	.677	1.081	.749	1.560
	hTG	.175	.188	.873	1	.350	1.192	.825	1.722
	lHDL(1)	.130	.166	.611	1	.434	1.138	.823	1.575
	hLDL(1)	-.089	.244	.132	1	.717	.915	.567	1.477
	Constant	-100.176	4.397	519.086	1	.000	.000		

a. Variable(s) entered on step 1: rs2464196A, MI, Age, BMI, Sex, HTN, DM, hChol, hTG, lHDL, hLDL.

Variables in the Equation

		B	S.E.	Wald	df	Sig.	Exp(B)	95% C.I.for EXP(B)	
								Lower	Upper
Step 1[a]	Sex(1)	.015	.252	.004	1	.953	1.015	.619	1.665
	MI(1)	-.134	.295	.207	1	.649	.874	.490	1.560
	HTN(1)	.037	.296	.016	1	.900	1.038	.581	1.854
	DM	-.259	.258	1.011	1	.315	.772	.466	1.279
	FH(1)	.184	.295	.389	1	.533	1.202	.674	2.143
	hChol(1)	.073	.265	.076	1	.783	1.076	.640	1.807
	hTG(1)	-.183	.267	.471	1	.492	.833	.494	1.404
	lHDL(1)	.126	.235	.286	1	.592	1.134	.716	1.797
	hLDL(1)	-.102	.346	.087	1	.769	.903	.458	1.779
	Age	-.006	.010	.325	1	.569	.994	.975	1.014
	BMI	3.349	.206	264.456	1	.000	28.483	19.023	42.648
	rs2464196R	.131	.269	.236	1	.627	1.140	.673	1.932
	Constant	-100.081	6.222	258.717	1	.000	.000		

a. Variable(s) entered on step 1: Sex, MI, HTN, DM, FH, hChol, hTG, lHDL, hLDL, Age, BMI, rs2464196R.

Variables in the Equation

		B	S.E.	Wald	df	Sig.	Exp(B)	95% C.I.for EXP(B)	
								Lower	Upper
Step 1[a]	rs2464196D	.001	.241	.000	1	.997	1.001	.625	1.604
	Sex(1)	.011	.253	.002	1	.965	1.011	.616	1.659
	MI(1)	-.133	.296	.201	1	.654	.876	.491	1.564
	HTN(1)	.032	.296	.012	1	.914	1.033	.578	1.845
	DM	-.253	.257	.962	1	.327	.777	.469	1.287
	FH(1)	.185	.296	.391	1	.532	1.203	.674	2.148
	hChol(1)	.079	.265	.089	1	.766	1.082	.644	1.817
	hTG(1)	-.187	.267	.490	1	.484	.830	.492	1.400
	lHDL(1)	.134	.235	.327	1	.567	1.144	.722	1.811
	hLDL(1)	-.095	.346	.076	1	.783	.909	.462	1.791
	Age	-.006	.010	.301	1	.584	.995	.975	1.014
	BMI	3.345	.206	264.927	1	.000	28.363	18.959	42.431
	Constant	-99.818	6.203	258.911	1	.000	.000		

a. Variable(s) entered on step 1: rs2464196D, Sex, MI, HTN, DM, FH, hChol, hTG, lHDL, hLDL, Age, BMI.

Variables in the Equation

		B	S.E.	Wald	df	Sig.	Exp(B)	95% C.I.for EXP(B)	
								Lower	Upper
Step 1[a]	rs2259816A	-.012	.164	.005	1	.942	.988	.717	1.362
	MI(1)	-.172	.211	.663	1	.415	.842	.557	1.274
	Age	-.005	.007	.494	1	.482	.995	.981	1.009
	BMI	3.266	.144	514.639	1	.000	26.219	19.772	34.768
	Sex(1)	-.010	.180	.003	1	.956	.990	.695	1.410
	HTN(1)	.042	.207	.042	1	.838	1.043	.695	1.566
	DM(1)	.157	.181	.754	1	.385	1.170	.821	1.669
	hChol(1)	.120	.191	.396	1	.529	1.127	.776	1.638
	hTG	.195	.191	1.040	1	.308	1.215	.836	1.767
	lHDL(1)	.142	.167	.718	1	.397	1.152	.830	1.600
	hLDL(1)	-.160	.249	.414	1	.520	.852	.522	1.389
	Constant	-97.665	4.367	500.088	1	.000	.000		

a. Variable(s) entered on step 1: rs2259816A, MI, Age, BMI, Sex, HTN, DM, hChol, hTG, lHDL, hLDL.

Variables in the Equation

		B	S.E.	Wald	df	Sig.	Exp(B)	95% C.I.for EXP(B)	
								Lower	Upper
Step 1[a]	rs2259816R	.057	.234	.059	1	.808	1.058	.669	1.675
	Sex(1)	.000	.256	.000	1	1.000	1.000	.605	1.652
	MI(1)	-.169	.299	.321	1	.571	.844	.470	1.517
	HTN(1)	.086	.298	.084	1	.772	1.090	.608	1.955
	DM	-.192	.260	.544	1	.461	.826	.496	1.374
	FH(1)	.246	.306	.645	1	.422	1.279	.702	2.330
	hChol(1)	.119	.269	.195	1	.659	1.126	.664	1.909
	hTG(1)	-.213	.272	.611	1	.434	.808	.474	1.378
	lHDL(1)	.144	.237	.369	1	.544	1.155	.726	1.838
	hLDL(1)	-.167	.354	.222	1	.637	.846	.423	1.693
	Age	-.006	.010	.352	1	.553	.994	.974	1.014
	BMI	3.265	.203	257.759	1	.000	26.174	17.570	38.991
	Constant	-97.476	6.146	251.562	1	.000	.000		

a. Variable(s) entered on step 1: rs2259816R, Sex, MI, HTN, DM, FH, hChol, hTG, lHDL, hLDL, Age, BMI.

Variables in the Equation

		B	S.E.	Wald	df	Sig.	Exp(B)	95% C.I.for EXP(B)	
								Lower	Upper
Step 1[a]	rs2259816D	-.122	.305	.160	1	.690	.885	.487	1.609
	Sex(1)	-.002	.256	.000	1	.994	.998	.605	1.647
	MI(1)	-.173	.299	.334	1	.563	.841	.468	1.512
	HTN(1)	.089	.298	.089	1	.765	1.093	.610	1.960
	DM	-.195	.260	.562	1	.453	.823	.494	1.370
	FH(1)	.242	.306	.628	1	.428	1.274	.700	2.318
	hChol(1)	.108	.270	.159	1	.690	1.114	.656	1.890
	hTG(1)	-.204	.271	.564	1	.453	.816	.479	1.388
	lHDL(1)	.137	.237	.336	1	.562	1.147	.721	1.825
	hLDL(1)	-.172	.354	.236	1	.627	.842	.421	1.685
	Age	-.007	.010	.409	1	.523	.993	.974	1.014
	BMI	3.264	.203	257.587	1	.000	26.164	17.562	38.979
	Constant	-97.122	6.147	249.628	1	.000	.000		

a. Variable(s) entered on step 1: rs2259816D, Sex, MI, HTN, DM, FH, hChol, hTG, lHDL, hLDL, Age, BMI.

rs1169310A

Variables in the Equation

		B	S.E.	Wald	df	Sig.	Exp(B)	95% C.I.for EXP(B)	
								Lower	Upper
Step 1[a]	rs1169310A	.052	.161	.105	1	.746	1.054	.768	1.446
	MI(1)	-.151	.209	.520	1	.471	.860	.571	1.295
	Age	-.005	.007	.485	1	.486	.995	.982	1.009
	BMI	3.365	.146	530.519	1	.000	28.937	21.731	38.531
	Sex(1)	-.010	.178	.003	1	.957	.990	.699	1.404
	HTN(1)	.007	.207	.001	1	.973	1.007	.672	1.510
	DM(1)	.265	.178	2.213	1	.137	1.303	.919	1.847
	hChol(1)	.027	.186	.020	1	.886	1.027	.714	1.478
	hTG	.170	.188	.818	1	.366	1.185	.820	1.712
	lHDL(1)	.114	.165	.472	1	.492	1.120	.810	1.549
	hLDL(1)	-.069	.243	.081	1	.777	.933	.579	1.504
	Constant	-100.768	4.424	518.806	1	.000	.000		

a. Variable(s) entered on step 1: rs1169310A, MI, Age, BMI, Sex, HTN, DM, hChol, hTG, lHDL, hLDL.

Variables in the Equation

		B	S.E.	Wald	df	Sig.	Exp(B)	95% C.I.for EXP(B)	
								Lower	Upper
Step 1[a]	rs1169310R	.156	.297	.277	1	.599	1.169	.653	2.094
	Sex(1)	-.001	.252	.000	1	.998	.999	.610	1.637
	MI(1)	-.148	.295	.253	1	.615	.862	.483	1.538
	HTN(1)	.037	.296	.016	1	.901	1.038	.581	1.853
	DM	-.292	.256	1.300	1	.254	.747	.452	1.234
	FH(1)	.143	.292	.242	1	.623	1.154	.652	2.044
	hChol(1)	.017	.263	.004	1	.949	1.017	.607	1.703
	hTG(1)	-.177	.266	.441	1	.507	.838	.497	1.412
	lHDL(1)	.112	.234	.229	1	.632	1.118	.707	1.769
	hLDL(1)	-.074	.345	.046	1	.830	.929	.473	1.824
	Age	-.006	.010	.320	1	.571	.994	.975	1.014
	BMI	3.366	.207	265.242	1	.000	28.948	19.307	43.403
	Constant	-100.507	6.238	259.596	1	.000	.000		

a. Variable(s) entered on step 1: rs1169310R, Sex, MI, HTN, DM, FH, hChol, hTG, lHDL, hLDL, Age, BMI.

Variables in the Equation

		B	S.E.	Wald	df	Sig.	Exp(B)	95% C.I.for EXP(B)	
								Lower	Upper
Step 1[a]	rs1169310D	.012	.231	.003	1	.959	1.012	.644	1.591
	Sex(1)	-.006	.252	.001	1	.982	.994	.606	1.631
	MI(1)	-.150	.295	.257	1	.612	.861	.482	1.536
	HTN(1)	.025	.296	.007	1	.933	1.025	.575	1.830
	DM	-.287	.256	1.250	1	.264	.751	.454	1.241
	FH(1)	.137	.292	.221	1	.638	1.147	.647	2.033
	hChol(1)	.025	.262	.009	1	.923	1.026	.613	1.716
	hTG(1)	-.177	.267	.442	1	.506	.838	.497	1.412
	lHDL(1)	.116	.234	.244	1	.621	1.123	.710	1.776
	hLDL(1)	-.072	.345	.044	1	.834	.930	.473	1.830
	Age	-.005	.010	.293	1	.588	.995	.975	1.014
	BMI	3.364	.206	265.447	1	.000	28.897	19.281	43.311
	Constant	-100.303	6.224	259.714	1	.000	.000		

a. Variable(s) entered on step 1: rs1169310D, Sex, MI, HTN, DM, FH, hChol, hTG, lHDL, hLDL, Age, BMI.

Variables in the Equation

		B	S.E.	Wald	df	Sig.	Exp(B)	95% C.I.for EXP(B)	
								Lower	Upper
Step 1[a]	rs1169313A	.066	.161	.168	1	.682	1.068	.779	1.465
	MI(1)	-.172	.208	.687	1	.407	.842	.560	1.265
	Age	-.005	.007	.479	1	.489	.995	.982	1.009
	BMI	3.346	.146	525.784	1	.000	28.382	21.323	37.778
	Sex(1)	.006	.178	.001	1	.972	1.006	.710	1.425
	HTN(1)	.018	.206	.008	1	.930	1.018	.679	1.526
	DM(1)	.257	.178	2.081	1	.149	1.293	.912	1.833
	hChol(1)	.027	.186	.020	1	.886	1.027	.713	1.478
	hTG	.171	.187	.833	1	.361	1.187	.822	1.714
	lHDL(1)	.129	.166	.605	1	.437	1.137	.822	1.574
	hLDL(1)	-.061	.243	.063	1	.802	.941	.584	1.516
	Constant	-100.234	4.417	514.885	1	.000	.000		

a. Variable(s) entered on step 1: rs1169313A, MI, Age, BMI, Sex, HTN, DM, hChol, hTG, lHDL, hLDL.

Variables in the Equation

		B	S.E.	Wald	df	Sig.	Exp(B)	95% C.I.for EXP(B)	
								Lower	Upper
Step 1[a]	rs1169313R	.184	.296	.387	1	.534	1.202	.673	2.148
	Sex(1)	.018	.251	.005	1	.944	1.018	.622	1.666
	MI(1)	-.167	.294	.321	1	.571	.846	.475	1.507
	HTN(1)	.054	.295	.033	1	.856	1.055	.591	1.882
	DM	-.286	.256	1.253	1	.263	.751	.455	1.240
	FH(1)	.171	.289	.348	1	.555	1.186	.673	2.090
	hChol(1)	.016	.263	.004	1	.951	1.016	.607	1.702
	hTG(1)	-.179	.266	.451	1	.502	.836	.497	1.409
	lHDL(1)	.128	.234	.296	1	.586	1.136	.718	1.799
	hLDL(1)	-.067	.345	.038	1	.846	.935	.476	1.838
	Age	-.006	.010	.330	1	.566	.994	.975	1.014
	BMI	3.346	.206	262.975	1	.000	28.385	18.944	42.531
	Constant	-100.000	6.226	258.003	1	.000	.000		

a. Variable(s) entered on step 1: rs1169313R, Sex, MI, HTN, DM, FH, hChol, hTG, lHDL, hLDL, Age, BMI.

Variables in the Equation

		B	S.E.	Wald	df	Sig.	Exp(B)	95% C.I.for EXP(B)	
								Lower	Upper
Step 1[a]	rs1169313D	.019	.232	.007	1	.934	1.019	.647	1.606
	Sex(1)	.010	.252	.002	1	.968	1.010	.616	1.656
	MI(1)	-.170	.295	.333	1	.564	.844	.474	1.503
	HTN(1)	.038	.295	.017	1	.896	1.039	.583	1.853
	DM	-.281	.256	1.208	1	.272	.755	.457	1.247
	FH(1)	.163	.290	.317	1	.573	1.177	.667	2.078
	hChol(1)	.026	.263	.010	1	.921	1.026	.613	1.717
	hTG(1)	-.180	.266	.455	1	.500	.836	.496	1.408
	lHDL(1)	.131	.235	.314	1	.575	1.140	.720	1.806
	hLDL(1)	-.066	.345	.037	1	.848	.936	.476	1.840
	Age	-.006	.010	.302	1	.583	.995	.975	1.014
	BMI	3.345	.206	263.133	1	.000	28.349	18.925	42.466
	Constant	-99.786	6.216	257.740	1	.000	.000		

a. Variable(s) entered on step 1: rs1169313D, Sex, MI, HTN, DM, FH, hChol, hTG, lHDL, hLDL, Age, BMI.

HNF1a vs Hypercholesterolemia

rs2393791D vs hChol

Variables in the Equation

		B	S.E.	Wald	df	Sig.	Exp(B)	95% C.I.for EXP(B)	
								Lower	Upper
Step 1[a]	rs2393791A	-.034	.053	.409	1	.523	.966	.870	1.073
	MI	.677	.066	105.743	1	.000	1.968	1.729	2.239
	Age	.013	.002	35.987	1	.000	1.013	1.009	1.017
	BMI	.019	.007	7.207	1	.007	1.019	1.005	1.033
	Sex(1)	.014	.060	.053	1	.817	1.014	.901	1.141
	HTN(1)	-.455	.072	39.609	1	.000	.634	.551	.731
	DM(1)	-.272	.058	21.756	1	.000	.762	.680	.854
	OBS(1)	-.138	.083	2.754	1	.097	.871	.741	1.025
	hTG(1)	-.882	.059	224.199	1	.000	.414	.369	.465
	lHDL(1)	.241	.055	19.182	1	.000	1.273	1.143	1.418
	hLDL(1)	-2.394	.084	813.379	1	.000	.091	.077	.108
	Constant	.573	.308	3.454	1	.063	1.774		

a. Variable(s) entered on step 1: rs2393791A, MI, Age, BMI, Sex, HTN, DM, OBS, hTG, lHDL, hLDL.

Variables in the Equation

		B	S.E.	Wald	df	Sig.	Exp(B)	95% C.I.for EXP(B)	
								Lower	Upper
Step 1[a]	rs2393791R(1)	-.040	.099	.166	1	.684	.960	.791	1.166
	Sex(1)	.005	.085	.003	1	.955	1.005	.850	1.188
	MI(1)	-.692	.093	54.955	1	.000	.501	.417	.601
	HTN(1)	-.459	.102	20.159	1	.000	.632	.517	.772
	DM	.303	.083	13.159	1	.000	1.354	1.149	1.594
	OBS(1)	-.138	.117	1.384	1	.239	.871	.692	1.096
	FH(1)	-.219	.098	4.960	1	.026	.803	.663	.974
	hTG(1)	-.874	.083	109.988	1	.000	.417	.354	.491
	lHDL(1)	.237	.078	9.268	1	.002	1.268	1.088	1.477
	hLDL(1)	-2.392	.119	404.396	1	.000	.091	.072	.115
	Age	.014	.003	20.344	1	.000	1.014	1.008	1.020
	BMI	.018	.010	3.131	1	.077	1.018	.998	1.038
	Constant	1.105	.438	6.359	1	.012	3.019		

a. Variable(s) entered on step 1: rs2393791R, Sex, MI, HTN, DM, OBS, FH, hTG, lHDL, hLDL, Age, BMI.

Variables in the Equation

		B	S.E.	Wald	df	Sig.	Exp(B)	95% C.I.for EXP(B)	
								Lower	Upper
Step 1[a]	rs2393791D	-.094	.076	1.525	1	.217	.910	.784	1.057
	Sex(1)	.006	.085	.005	1	.942	1.006	.851	1.189
	MI(1)	-.688	.093	54.352	1	.000	.503	.419	.603
	HTN(1)	-.463	.102	20.487	1	.000	.629	.515	.769
	DM	.299	.083	12.861	1	.000	1.349	1.145	1.589
	OBS(1)	-.136	.117	1.337	1	.248	.873	.693	1.099
	FH(1)	-.219	.098	4.965	1	.026	.803	.663	.974
	hTG(1)	-.879	.083	110.918	1	.000	.415	.353	.489
	lHDL(1)	.238	.078	9.349	1	.002	1.269	1.089	1.479
	hLDL(1)	-2.387	.119	402.788	1	.000	.092	.073	.116
	Age	.014	.003	20.315	1	.000	1.014	1.008	1.020
	BMI	.018	.010	3.192	1	.074	1.018	.998	1.038
	Constant	1.217	.447	7.425	1	.006	3.376		

a. Variable(s) entered on step 1: rs2393791D, Sex, MI, HTN, DM, OBS, FH, hTG, lHDL, hLDL, Age, BMI.

Variables in the Equation

		B	S.E.	Wald	df	Sig.	Exp(B)	95% C.I.for EXP(B)	
								Lower	Upper
Step 1[a]	rs7310409A	-.057	.053	1.126	1	.289	.945	.851	1.049
	MI	.682	.066	107.574	1	.000	1.977	1.738	2.249
	Age	.013	.002	35.577	1	.000	1.013	1.009	1.017
	BMI	.019	.007	7.312	1	.007	1.019	1.005	1.033
	Sex(1)	.010	.060	.028	1	.867	1.010	.898	1.136
	HTN(1)	-.458	.072	40.224	1	.000	.632	.549	.729
	DM(1)	-.273	.058	22.061	1	.000	.761	.679	.853
	OBS(1)	-.139	.083	2.829	1	.093	.870	.740	1.023
	hTG(1)	-.881	.059	224.586	1	.000	.414	.369	.465
	lHDL(1)	.239	.055	18.958	1	.000	1.270	1.141	1.415
	hLDL(1)	-2.411	.084	826.888	1	.000	.090	.076	.106
	Constant	.620	.307	4.059	1	.044	1.858		

a. Variable(s) entered on step 1: rs7310409A, MI, Age, BMI, Sex, HTN, DM, OBS, hTG, lHDL, hLDL.

Variables in the Equation

		B	S.E.	Wald	df	Sig.	Exp(B)	95% C.I.for EXP(B)	
								Lower	Upper
Step 1[a]	rs7310409R	.008	.100	.007	1	.933	1.008	.830	1.226
	Sex(1)	.001	.085	.000	1	.990	1.001	.847	1.183
	MI(1)	-.697	.093	55.891	1	.000	.498	.415	.598
	HTN(1)	-.463	.102	20.473	1	.000	.630	.515	.769
	DM	.305	.083	13.419	1	.000	1.357	1.152	1.597
	OBS(1)	-.139	.117	1.420	1	.233	.870	.691	1.094
	FH(1)	-.226	.098	5.288	1	.021	.798	.658	.967
	hTG(1)	-.873	.083	110.144	1	.000	.418	.355	.492
	lHDL(1)	.235	.078	9.128	1	.003	1.265	1.086	1.473
	hLDL(1)	-2.408	.119	411.243	1	.000	.090	.071	.114
	Age	.014	.003	20.150	1	.000	1.014	1.008	1.020
	BMI	.018	.010	3.175	1	.075	1.018	.998	1.038
	Constant	1.086	.445	5.967	1	.015	2.963		

a. Variable(s) entered on step 1: rs7310409R, Sex, MI, HTN, DM, OBS, FH, hTG, lHDL, hLDL, Age, BMI.

Variables in the Equation

		B	S.E.	Wald	df	Sig.	Exp(B)	95% C.I.for EXP(B)	
								Lower	Upper
Step 1[a]	rs7310409D	-.120	.076	2.513	1	.113	.887	.765	1.029
	Sex(1)	.002	.085	.001	1	.977	1.002	.848	1.184
	MI(1)	-.694	.093	55.491	1	.000	.500	.416	.600
	HTN(1)	-.468	.102	20.903	1	.000	.626	.513	.765
	DM	.301	.083	13.091	1	.000	1.352	1.148	1.592
	OBS(1)	-.136	.117	1.351	1	.245	.873	.694	1.098
	FH(1)	-.225	.098	5.265	1	.022	.798	.658	.968
	hTG(1)	-.878	.083	111.101	1	.000	.416	.353	.489
	lHDL(1)	.237	.078	9.302	1	.002	1.268	1.088	1.476
	hLDL(1)	-2.404	.119	409.601	1	.000	.090	.072	.114
	Age	.014	.003	20.116	1	.000	1.014	1.008	1.020
	BMI	.018	.010	3.271	1	.071	1.018	.998	1.038
	Constant	1.279	.445	8.254	1	.004	3.593		

a. Variable(s) entered on step 1: rs7310409D, Sex, MI, HTN, DM, OBS, FH, hTG, lHDL, hLDL, Age, BMI.

rs2259820A

Variables in the Equation

		B	S.E.	Wald	df	Sig.	Exp(B)	95% C.I.for EXP(B)	
								Lower	Upper
Step 1[a]	rs2259820A	.026	.053	.245	1	.620	1.027	.925	1.139
	MI	.664	.067	98.943	1	.000	1.942	1.704	2.213
	Age	.013	.002	36.505	1	.000	1.013	1.009	1.018
	BMI	.016	.007	4.923	1	.027	1.016	1.002	1.031
	Sex(1)	.036	.061	.345	1	.557	1.036	.920	1.168
	HTN(1)	-.442	.073	36.352	1	.000	.643	.557	.742
	DM(1)	-.281	.059	22.684	1	.000	.755	.673	.848
	OBS(1)	-.152	.084	3.255	1	.071	.859	.729	1.013
	hTG(1)	-.888	.060	220.679	1	.000	.412	.366	.463
	lHDL(1)	.246	.056	19.446	1	.000	1.279	1.146	1.426
	hLDL(1)	-2.428	.085	812.987	1	.000	.088	.075	.104
	Constant	.587	.317	3.430	1	.064	1.798		

a. Variable(s) entered on step 1: rs2259820A, MI, Age, BMI, Sex, HTN, DM, OBS, hTG, lHDL, hLDL.

Variables in the Equation

		B	S.E.	Wald	df	Sig.	Exp(B)	95% C.I.for EXP(B)	
								Lower	Upper
Step 1[a]	rs2259820R	.056	.082	.469	1	.493	1.058	.901	1.241
	Sex(1)	.028	.086	.107	1	.744	1.029	.869	1.218
	MI(1)	-.676	.095	51.102	1	.000	.509	.422	.612
	HTN(1)	-.450	.104	18.833	1	.000	.638	.520	.781
	DM	.312	.085	13.655	1	.000	1.367	1.158	1.613
	OBS(1)	-.150	.119	1.580	1	.209	.861	.682	1.087
	FH(1)	-.236	.099	5.675	1	.017	.789	.650	.959
	hTG(1)	-.883	.085	108.930	1	.000	.413	.350	.488
	lHDL(1)	.243	.079	9.503	1	.002	1.275	1.093	1.488
	hLDL(1)	-2.422	.121	402.605	1	.000	.089	.070	.112
	Age	.014	.003	20.745	1	.000	1.015	1.008	1.021
	BMI	.015	.010	2.129	1	.145	1.015	.995	1.036
	Constant	1.079	.454	5.635	1	.018	2.941		

a. Variable(s) entered on step 1: rs2259820R, Sex, MI, HTN, DM, OBS, FH, hTG, lHDL, hLDL, Age, BMI.

Variables in the Equation

		B	S.E.	Wald	df	Sig.	Exp(B)	95% C.I.for EXP(B)	
								Lower	Upper
Step 1[a]	rs2259820D	.013	.088	.023	1	.879	1.013	.853	1.204
	Sex(1)	.028	.086	.106	1	.745	1.028	.869	1.218
	MI(1)	-.678	.095	51.453	1	.000	.507	.422	.611
	HTN(1)	-.448	.104	18.707	1	.000	.639	.521	.783
	DM	.314	.085	13.778	1	.000	1.369	1.160	1.615
	OBS(1)	-.151	.119	1.611	1	.204	.860	.681	1.086
	FH(1)	-.237	.099	5.702	1	.017	.789	.649	.958
	hTG(1)	-.881	.085	108.575	1	.000	.414	.351	.489
	lHDL(1)	.243	.079	9.483	1	.002	1.275	1.092	1.488
	hLDL(1)	-2.425	.121	403.816	1	.000	.089	.070	.112
	Age	.014	.003	20.648	1	.000	1.014	1.008	1.021
	BMI	.015	.010	2.097	1	.148	1.015	.995	1.035
	Constant	1.136	.465	5.956	1	.015	3.113		

a. Variable(s) entered on step 1: rs2259820D, Sex, MI, HTN, DM, OBS, FH, hTG, lHDL, hLDL, Age, BMI.

rs2464196A

Variables in the Equation

		B	S.E.	Wald	df	Sig.	Exp(B)	95% C.I.for EXP(B)	
								Lower	Upper
Step 1[a]	rs2464196A(1)	.022	.053	.172	1	.678	1.022	.922	1.133
	MI	.681	.066	106.337	1	.000	1.976	1.736	2.248
	Age	.013	.002	36.646	1	.000	1.013	1.009	1.018
	BMI	.020	.007	8.022	1	.005	1.020	1.006	1.034
	Sex(1)	.006	.060	.010	1	.919	1.006	.894	1.132
	HTN(1)	-.458	.073	39.825	1	.000	.633	.549	.729
	DM(1)	-.294	.058	25.230	1	.000	.745	.665	.836
	OBS(1)	-.111	.083	1.792	1	.181	.895	.760	1.053
	hTG(1)	-.872	.059	217.501	1	.000	.418	.372	.469
	lHDL(1)	.243	.055	19.293	1	.000	1.275	1.144	1.420
	hLDL(1)	-2.411	.084	816.728	1	.000	.090	.076	.106
	Constant	.471	.302	2.428	1	.119	1.602		

a. Variable(s) entered on step 1: rs2464196A, MI, Age, BMI, Sex, HTN, DM, OBS, hTG, lHDL, hLDL.

Variables in the Equation

		B	S.E.	Wald	df	Sig.	Exp(B)	95% C.I.for EXP(B)	
								Lower	Upper
Step 1[a]	rs2464196R	-.012	.087	.018	1	.894	.988	.833	1.173
	Sex(1)	-.003	.085	.001	1	.976	.997	.844	1.179
	MI(1)	-.695	.094	55.197	1	.000	.499	.415	.599
	HTN(1)	-.464	.103	20.408	1	.000	.629	.514	.769
	DM	.325	.084	15.016	1	.000	1.384	1.174	1.631
	OBS(1)	-.112	.118	.899	1	.343	.894	.710	1.126
	FH(1)	-.224	.099	5.118	1	.024	.800	.659	.971
	hTG(1)	-.865	.084	106.812	1	.000	.421	.357	.496
	lHDL(1)	.239	.078	9.347	1	.002	1.270	1.090	1.480
	hLDL(1)	-2.408	.120	405.842	1	.000	.090	.071	.114
	Age	.014	.003	20.597	1	.000	1.014	1.008	1.021
	BMI	.019	.010	3.523	1	.061	1.019	.999	1.039
	Constant	1.030	.445	5.354	1	.021	2.800		

a. Variable(s) entered on step 1: rs2464196R, Sex, MI, HTN, DM, OBS, FH, hTG, lHDL, hLDL, Age, BMI.

Variables in the Equation

		B	S.E.	Wald	df	Sig.	Exp(B)	95% C.I.for EXP(B)	
								Lower	Upper
Step 1[a]	rs2464196D	-.046	.081	.319	1	.572	.955	.815	1.120
	Sex(1)	-.002	.085	.001	1	.980	.998	.844	1.180
	MI(1)	-.694	.094	54.882	1	.000	.500	.416	.600
	HTN(1)	-.465	.103	20.537	1	.000	.628	.513	.768
	DM	.324	.084	14.907	1	.000	1.382	1.173	1.629
	OBS(1)	-.110	.118	.882	1	.348	.895	.711	1.128
	FH(1)	-.223	.099	5.091	1	.024	.800	.659	.971
	hTG(1)	-.866	.084	107.045	1	.000	.420	.357	.495
	lHDL(1)	.239	.078	9.365	1	.002	1.270	1.090	1.480
	hLDL(1)	-2.406	.120	404.989	1	.000	.090	.071	.114
	Age	.014	.003	20.666	1	.000	1.014	1.008	1.021
	BMI	.019	.010	3.558	1	.059	1.019	.999	1.039
	Constant	1.087	.450	5.825	1	.016	2.964		

a. Variable(s) entered on step 1: rs2464196D, Sex, MI, HTN, DM, OBS, FH, hTG, lHDL, hLDL, Age, BMI.

Variables in the Equation

		B	S.E.	Wald	df	Sig.	Exp(B)	95% C.I.for EXP(B)	
								Lower	Upper
Step 1[a]	rs2259816A	.014	.054	.063	1	.802	1.014	.912	1.127
	MI	.699	.068	106.491	1	.000	2.011	1.761	2.297
	Age	.013	.002	31.777	1	.000	1.013	1.008	1.017
	BMI	.021	.007	8.079	1	.004	1.021	1.006	1.035
	Sex(1)	.053	.062	.735	1	.391	1.054	.934	1.190
	HTN(1)	-.461	.074	38.869	1	.000	.631	.546	.729
	DM(1)	-.269	.060	20.459	1	.000	.764	.680	.858
	OBS(1)	-.129	.085	2.289	1	.130	.879	.745	1.039
	hTG(1)	-.869	.060	207.366	1	.000	.419	.372	.472
	lHDL(1)	.243	.056	18.621	1	.000	1.275	1.142	1.424
	hLDL(1)	-2.450	.087	789.272	1	.000	.086	.073	.102
	Constant	.477	.321	2.217	1	.136	1.612		

a. Variable(s) entered on step 1: rs2259816A, MI, Age, BMI, Sex, HTN, DM, OBS, hTG, lHDL, hLDL.

Variables in the Equation

		B	S.E.	Wald	df	Sig.	Exp(B)	95% C.I.for EXP(B)	
								Lower	Upper
Step 1[a]	rs2259816R	.063	.079	.627	1	.428	1.065	.911	1.244
	Sex(1)	.044	.087	.255	1	.614	1.045	.880	1.241
	MI(1)	-.712	.096	54.990	1	.000	.491	.406	.592
	HTN(1)	-.469	.105	20.120	1	.000	.625	.509	.768
	DM	.299	.085	12.257	1	.000	1.348	1.141	1.593
	OBS(1)	-.128	.120	1.138	1	.286	.880	.695	1.113
	FH(1)	-.231	.101	5.269	1	.022	.793	.651	.967
	hTG(1)	-.866	.085	102.720	1	.000	.420	.356	.497
	lHDL(1)	.240	.080	9.071	1	.003	1.271	1.087	1.486
	hLDL(1)	-2.442	.124	390.610	1	.000	.087	.068	.111
	Age	.014	.003	18.221	1	.000	1.014	1.007	1.020
	BMI	.019	.010	3.547	1	.060	1.020	.999	1.040
	Constant	.989	.458	4.659	1	.031	2.689		

a. Variable(s) entered on step 1: rs2259816R, Sex, MI, HTN, DM, OBS, FH, hTG, lHDL, hLDL, Age, BMI.

Variables in the Equation

		B	S.E.	Wald	df	Sig.	Exp(B)	95% C.I.for EXP(B)	
								Lower	Upper
Step 1[a]	rs2259816D	-.043	.096	.198	1	.656	.958	.794	1.156
	Sex(1)	.044	.087	.248	1	.619	1.045	.880	1.240
	MI(1)	-.715	.096	55.486	1	.000	.489	.405	.590
	HTN(1)	-.467	.105	19.942	1	.000	.627	.511	.769
	DM	.301	.085	12.468	1	.000	1.351	1.143	1.597
	OBS(1)	-.129	.120	1.160	1	.282	.879	.694	1.112
	FH(1)	-.230	.101	5.196	1	.023	.795	.652	.968
	hTG(1)	-.863	.085	102.139	1	.000	.422	.357	.499
	lHDL(1)	.239	.080	9.042	1	.003	1.271	1.087	1.485
	hLDL(1)	-2.446	.124	391.962	1	.000	.087	.068	.110
	Age	.014	.003	18.152	1	.000	1.014	1.007	1.020
	BMI	.019	.010	3.483	1	.062	1.019	.999	1.040
	Constant	1.159	.476	5.920	1	.015	3.185		

a. Variable(s) entered on step 1: rs2259816D, Sex, MI, HTN, DM, OBS, FH, hTG, lHDL, hLDL, Age, BMI.

Variables in the Equation

		B	S.E.	Wald	df	Sig.	Exp(B)	95% C.I.for EXP(B)	
								Lower	Upper
Step 1[a]	rs1169310A	-.034	.053	.414	1	.520	.966	.871	1.072
	MI	.683	.066	107.236	1	.000	1.980	1.740	2.253
	Age	.013	.002	35.275	1	.000	1.013	1.009	1.017
	BMI	.020	.007	7.950	1	.005	1.020	1.006	1.034
	Sex(1)	.015	.060	.064	1	.800	1.015	.902	1.143
	HTN(1)	-.459	.072	40.045	1	.000	.632	.549	.729
	DM(1)	-.279	.058	22.939	1	.000	.757	.675	.848
	OBS(1)	-.135	.083	2.660	1	.103	.874	.743	1.028
	hTG(1)	-.865	.059	214.991	1	.000	.421	.375	.473
	lHDL(1)	.236	.055	18.341	1	.000	1.266	1.136	1.410
	hLDL(1)	-2.438	.084	832.685	1	.000	.087	.074	.103
	Constant	.574	.308	3.471	1	.062	1.775		

a. Variable(s) entered on step 1: rs1169310A, MI, Age, BMI, Sex, HTN, DM, OBS, hTG, lHDL, hLDL.

Variables in the Equation

		B	S.E.	Wald	df	Sig.	Exp(B)	95% C.I.for EXP(B)	
								Lower	Upper
Step 1[a]	rs1169310R	.005	.095	.003	1	.956	1.005	.834	1.211
	Sex(1)	.006	.085	.006	1	.940	1.006	.851	1.190
	MI(1)	-.698	.093	55.679	1	.000	.498	.414	.598
	HTN(1)	-.464	.103	20.461	1	.000	.629	.514	.769
	DM	.312	.084	13.968	1	.000	1.366	1.160	1.610
	OBS(1)	-.135	.117	1.322	1	.250	.874	.694	1.100
	FH(1)	-.229	.099	5.408	1	.020	.795	.655	.965
	hTG(1)	-.857	.083	105.384	1	.000	.424	.360	.500
	lHDL(1)	.232	.078	8.893	1	.003	1.262	1.083	1.470
	hLDL(1)	-2.435	.120	413.931	1	.000	.088	.069	.111
	Age	.014	.003	19.964	1	.000	1.014	1.008	1.020
	BMI	.019	.010	3.475	1	.062	1.019	.999	1.039
	Constant	1.070	.445	5.786	1	.016	2.916		

a. Variable(s) entered on step 1: rs1169310R, Sex, MI, HTN, DM, OBS, FH, hTG, lHDL, hLDL, Age, BMI.

Variables in the Equation

		B	S.E.	Wald	df	Sig.	Exp(B)	95% C.I.for EXP(B)	
								Lower	Upper
Step 1[a]	rs1169310D	-.079	.077	1.032	1	.310	.924	.794	1.076
	Sex(1)	.007	.085	.007	1	.931	1.007	.852	1.191
	MI(1)	-.695	.094	55.220	1	.000	.499	.416	.600
	HTN(1)	-.466	.103	20.644	1	.000	.627	.513	.767
	DM	.309	.084	13.691	1	.000	1.362	1.157	1.605
	OBS(1)	-.133	.117	1.283	1	.257	.876	.696	1.102
	FH(1)	-.229	.099	5.418	1	.020	.795	.655	.964
	hTG(1)	-.861	.084	106.097	1	.000	.423	.359	.498
	lHDL(1)	.233	.078	8.953	1	.003	1.263	1.084	1.471
	hLDL(1)	-2.431	.120	412.336	1	.000	.088	.070	.111
	Age	.014	.003	20.110	1	.000	1.014	1.008	1.020
	BMI	.019	.010	3.564	1	.059	1.019	.999	1.039
	Constant	1.193	.446	7.164	1	.007	3.298		

a. Variable(s) entered on step 1: rs1169310D, Sex, MI, HTN, DM, OBS, FH, hTG, lHDL, hLDL, Age, BMI.

Variables in the Equation

		B	S.E.	Wald	df	Sig.	Exp(B)	95% C.I.for EXP(B)	
								Lower	Upper
Step 1[a]	rs1169313A	-.023	.054	.182	1	.670	.977	.880	1.085
	MI	.682	.067	105.256	1	.000	1.979	1.737	2.254
	Age	.013	.002	36.339	1	.000	1.013	1.009	1.018
	BMI	.019	.007	6.837	1	.009	1.019	1.005	1.033
	Sex(1)	.034	.061	.319	1	.572	1.035	.919	1.166
	HTN(1)	-.458	.073	39.091	1	.000	.632	.548	.730
	DM(1)	-.278	.059	22.397	1	.000	.757	.675	.850
	OBS(1)	-.144	.084	2.962	1	.085	.866	.735	1.020
	hTG(1)	-.901	.060	229.135	1	.000	.406	.361	.456
	lHDL(1)	.237	.056	18.228	1	.000	1.268	1.137	1.413
	hLDL(1)	-2.422	.085	818.285	1	.000	.089	.075	.105
	Constant	.579	.311	3.460	1	.063	1.785		

a. Variable(s) entered on step 1: rs1169313A, MI, Age, BMI, Sex, HTN, DM, OBS, hTG, lHDL, hLDL.

Variables in the Equation

		B	S.E.	Wald	df	Sig.	Exp(B)	95% C.I.for EXP(B)	
								Lower	Upper
Step 1[a]	rs1169313R	.026	.096	.074	1	.785	1.027	.851	1.239
	Sex(1)	.026	.086	.091	1	.763	1.026	.867	1.215
	MI(1)	-.696	.094	54.473	1	.000	.499	.415	.600
	HTN(1)	-.463	.104	19.949	1	.000	.629	.514	.771
	DM	.310	.084	13.535	1	.000	1.363	1.156	1.608
	OBS(1)	-.143	.118	1.459	1	.227	.867	.687	1.093
	FH(1)	-.223	.099	5.041	1	.025	.800	.658	.972
	hTG(1)	-.894	.084	112.714	1	.000	.409	.347	.482
	lHDL(1)	.234	.079	8.863	1	.003	1.264	1.083	1.474
	hLDL(1)	-2.420	.120	406.988	1	.000	.089	.070	.112
	Age	.014	.003	20.415	1	.000	1.014	1.008	1.021
	BMI	.018	.010	2.990	1	.084	1.018	.998	1.038
	Constant	1.063	.451	5.554	1	.018	2.895		

a. Variable(s) entered on step 1: rs1169313R, Sex, MI, HTN, DM, OBS, FH, hTG, lHDL, hLDL, Age, BMI.

Variables in the Equation

		B	S.E.	Wald	df	Sig.	Exp(B)	95% C.I.for EXP(B)	
								Lower	Upper
Step 1[a]	rs1169313D	-.067	.078	.740	1	.390	.935	.802	1.090
	Sex(1)	.027	.086	.096	1	.756	1.027	.868	1.216
	MI(1)	-.693	.094	54.041	1	.000	.500	.416	.602
	HTN(1)	-.465	.104	20.119	1	.000	.628	.512	.770
	DM	.307	.084	13.305	1	.000	1.360	1.153	1.604
	OBS(1)	-.142	.118	1.439	1	.230	.868	.688	1.094
	FH(1)	-.223	.099	5.044	1	.025	.800	.658	.972
	hTG(1)	-.898	.084	113.357	1	.000	.408	.345	.481
	lHDL(1)	.235	.079	8.917	1	.003	1.264	1.084	1.475
	hLDL(1)	-2.416	.120	405.432	1	.000	.089	.071	.113
	Age	.014	.003	20.530	1	.000	1.014	1.008	1.021
	BMI	.018	.010	3.054	1	.081	1.018	.998	1.038
	Constant	1.195	.451	7.016	1	.008	3.304		

a. Variable(s) entered on step 1: rs1169313D, Sex, MI, HTN, DM, OBS, FH, hTG, lHDL, hLDL, Age, BMI.

HNF1a vs Hypertriglyceridaema

Variables in the Equation

		B	S.E.	Wald	df	Sig.	Exp(B)	95% C.I.for EXP(B) Lower	Upper
Step 1[a]	rs2393791A	.129	.054	5.586	1	.018	1.137	1.022	1.265
	MI(1)	-.227	.067	11.508	1	.001	.797	.699	.909
	Age	-.013	.002	36.806	1	.000	.987	.982	.991
	BMI	.027	.007	13.664	1	.000	1.027	1.013	1.042
	Sex(1)	.181	.062	8.486	1	.004	1.198	1.061	1.353
	HTN(1)	.041	.071	.323	1	.570	1.041	.905	1.198
	DM(1)	-.568	.061	86.397	1	.000	.566	.502	.639
	OBS(1)	.121	.085	2.007	1	.157	1.129	.955	1.334
	lHDL(1)	-.888	.055	256.442	1	.000	.411	.369	.459
	hLDL(1)	-.737	.073	102.147	1	.000	.478	.415	.552
	hChol(1)	-.873	.059	220.167	1	.000	.418	.372	.469
	Constant	.445	.313	2.024	1	.155	1.561		

a. Variable(s) entered on step 1: rs2393791A, MI, Age, BMI, Sex, HTN, DM, OBS, lHDL, hLDL, hChol.

Variables in the Equation

		B	S.E.	Wald	df	Sig.	Exp(B)	95% C.I.for EXP(B) Lower	Upper
Step 1[a]	rs2393791R(1)	-.127	.100	1.618	1	.203	.881	.724	1.071
	Sex(1)	.168	.088	3.659	1	.056	1.183	.996	1.405
	MI(1)	-.241	.095	6.485	1	.011	.786	.653	.946
	HTN(1)	.033	.101	.104	1	.747	1.033	.847	1.260
	DM	.602	.088	47.296	1	.000	1.826	1.538	2.167
	OBS(1)	.121	.121	1.004	1	.316	1.129	.891	1.431
	FH(1)	-.257	.097	6.988	1	.008	.773	.639	.936
	hChol(1)	-.866	.083	108.154	1	.000	.421	.357	.495
	lHDL(1)	-.894	.079	129.503	1	.000	.409	.351	.477
	hLDL(1)	-.728	.103	49.664	1	.000	.483	.394	.591
	Age	-.012	.003	15.283	1	.000	.988	.982	.994
	BMI	.025	.010	5.960	1	.015	1.026	1.005	1.047
	Constant	.340	.441	.595	1	.441	1.405		

a. Variable(s) entered on step 1: rs2393791R, Sex, MI, HTN, DM, OBS, FH, hChol, lHDL, hLDL, Age, BMI.

Variables in the Equation

		B	S.E.	Wald	df	Sig.	Exp(B)	95% C.I.for EXP(B)	
								Lower	Upper
Step 1[a]	rs2393791D	.186	.078	5.652	1	.017	1.205	1.033	1.405
	Sex(1)	.170	.088	3.757	1	.053	1.186	.998	1.409
	MI(1)	-.246	.095	6.735	1	.009	.782	.649	.942
	HTN(1)	.035	.101	.116	1	.733	1.035	.849	1.262
	DM	.606	.088	47.898	1	.000	1.834	1.544	2.177
	OBS(1)	.119	.121	.965	1	.326	1.126	.888	1.428
	FH(1)	-.258	.097	7.021	1	.008	.773	.638	.935
	hChol(1)	-.871	.083	109.227	1	.000	.419	.356	.493
	lHDL(1)	-.895	.079	129.633	1	.000	.409	.350	.477
	hLDL(1)	-.731	.103	50.009	1	.000	.481	.393	.589
	Age	-.013	.003	15.730	1	.000	.988	.981	.994
	BMI	.025	.010	5.880	1	.015	1.025	1.005	1.046
	Constant	-.050	.448	.012	1	.911	.951		

a. Variable(s) entered on step 1: rs2393791D, Sex, MI, HTN, DM, OBS, FH, hChol, lHDL, hLDL, Age, BMI.

Variables in the Equation

		B	S.E.	Wald	df	Sig.	Exp(B)	95% C.I.for EXP(B)	
								Lower	Upper
Step 1[a]	rs7310409A	.117	.054	4.639	1	.031	1.124	1.011	1.251
	MI(1)	-.222	.067	11.103	1	.001	.801	.703	.913
	Age	-.014	.002	38.513	1	.000	.986	.982	.991
	BMI	.027	.007	14.189	1	.000	1.028	1.013	1.042
	Sex(1)	.173	.062	7.796	1	.005	1.188	1.053	1.341
	HTN(1)	.030	.071	.182	1	.670	1.031	.896	1.185
	DM(1)	-.582	.061	91.000	1	.000	.559	.496	.630
	OBS(1)	.129	.085	2.299	1	.129	1.138	.963	1.344
	lHDL(1)	-.892	.055	260.590	1	.000	.410	.368	.457
	hLDL(1)	-.725	.073	99.448	1	.000	.484	.420	.558
	hChol(1)	-.872	.059	220.555	1	.000	.418	.373	.469
	Constant	.461	.312	2.183	1	.140	1.585		

a. Variable(s) entered on step 1: rs7310409A, MI, Age, BMI, Sex, HTN, DM, OBS, lHDL, hLDL, hChol.

Variables in the Equation

		B	S.E.	Wald	df	Sig.	Exp(B)	95% C.I.for EXP(B)	
								Lower	Upper
Step 1[a]	rs7310409R	.117	.101	1.360	1	.244	1.125	.923	1.370
	Sex(1)	.161	.088	3.361	1	.067	1.174	.989	1.394
	MI(1)	-.237	.094	6.326	1	.012	.789	.655	.949
	HTN(1)	.022	.101	.047	1	.829	1.022	.839	1.246
	DM	.616	.087	49.760	1	.000	1.851	1.560	2.197
	OBS(1)	.130	.120	1.163	1	.281	1.139	.899	1.442
	FH(1)	-.258	.097	7.049	1	.008	.773	.639	.935
	hChol(1)	-.865	.083	108.314	1	.000	.421	.358	.496
	lHDL(1)	-.897	.078	131.413	1	.000	.408	.350	.475
	hLDL(1)	-.717	.103	48.458	1	.000	.488	.399	.597
	Age	-.013	.003	16.189	1	.000	.987	.981	.993
	BMI	.026	.010	6.246	1	.012	1.026	1.006	1.047
	Constant	.083	.445	.035	1	.852	1.087		

a. Variable(s) entered on step 1: rs7310409R, Sex, MI, HTN, DM, OBS, FH, hChol, lHDL, hLDL, Age, BMI.

Variables in the Equation

		B	S.E.	Wald	df	Sig.	Exp(B)	95% C.I.for EXP(B)	
								Lower	Upper
Step 1[a]	rs7310409D	.168	.078	4.654	1	.031	1.183	1.015	1.379
	Sex(1)	.163	.088	3.451	1	.063	1.177	.991	1.397
	MI(1)	-.239	.094	6.405	1	.011	.787	.654	.948
	HTN(1)	.026	.101	.064	1	.800	1.026	.842	1.251
	DM	.620	.087	50.298	1	.000	1.859	1.566	2.206
	OBS(1)	.127	.121	1.107	1	.293	1.135	.896	1.438
	FH(1)	-.259	.097	7.121	1	.008	.771	.638	.933
	hChol(1)	-.870	.083	109.352	1	.000	.419	.356	.493
	lHDL(1)	-.900	.078	131.940	1	.000	.407	.349	.474
	hLDL(1)	-.719	.103	48.688	1	.000	.487	.398	.596
	Age	-.013	.003	16.306	1	.000	.987	.981	.993
	BMI	.026	.010	6.154	1	.013	1.026	1.005	1.047
	Constant	-.040	.446	.008	1	.928	.960		

a. Variable(s) entered on step 1: rs7310409D, Sex, MI, HTN, DM, OBS, FH, hChol, lHDL, hLDL, Age, BMI.

Variables in the Equation

		B	S.E.	Wald	df	Sig.	Exp(B)	95% C.I.for EXP(B)	
								Lower	Upper
Step 1[a]	rs2259820A	-.130	.054	5.815	1	.016	.878	.789	.976
	MI(1)	-.217	.068	10.261	1	.001	.805	.705	.919
	Age	-.014	.002	40.241	1	.000	.986	.981	.990
	BMI	.031	.007	17.877	1	.000	1.032	1.017	1.047
	Sex(1)	.177	.063	7.995	1	.005	1.194	1.056	1.349
	HTN(1)	.030	.072	.173	1	.677	1.031	.894	1.188
	DM(1)	-.594	.062	91.724	1	.000	.552	.489	.624
	OBS(1)	.153	.086	3.150	1	.076	1.166	.984	1.381
	lHDL(1)	-.897	.056	255.715	1	.000	.408	.365	.455
	hLDL(1)	-.716	.074	93.738	1	.000	.489	.423	.565
	hChol(1)	-.879	.060	216.926	1	.000	.415	.369	.467
	Constant	.719	.323	4.951	1	.026	2.052		

a. Variable(s) entered on step 1: rs2259820A, MI, Age, BMI, Sex, HTN, DM, OBS, lHDL, hLDL, hChol.

Variables in the Equation

		B	S.E.	Wald	df	Sig.	Exp(B)	95% C.I.for EXP(B)	
								Lower	Upper
Step 1[a]	rs2259820R	-.207	.085	5.989	1	.014	.813	.689	.960
	Sex(1)	.168	.089	3.575	1	.059	1.183	.994	1.407
	MI(1)	-.237	.096	6.082	1	.014	.789	.654	.953
	HTN(1)	.025	.103	.061	1	.805	1.026	.839	1.254
	DM	.628	.089	50.111	1	.000	1.874	1.575	2.230
	OBS(1)	.151	.123	1.522	1	.217	1.163	.915	1.479
	FH(1)	-.240	.098	5.952	1	.015	.787	.649	.954
	hChol(1)	-.875	.084	107.238	1	.000	.417	.353	.492
	lHDL(1)	-.902	.079	128.828	1	.000	.406	.347	.474
	hLDL(1)	-.713	.105	46.336	1	.000	.490	.399	.602
	Age	-.013	.003	17.566	1	.000	.987	.980	.993
	BMI	.030	.011	8.035	1	.005	1.030	1.009	1.052
	Constant	.374	.456	.674	1	.412	1.453		

a. Variable(s) entered on step 1: rs2259820R, Sex, MI, HTN, DM, OBS, FH, hChol, lHDL, hLDL, Age, BMI.

Variables in the Equation

		B	S.E.	Wald	df	Sig.	Exp(B)	95% C.I.for EXP(B)	
								Lower	Upper
Step 1[a]	rs2259820D	-.118	.089	1.755	1	.185	.888	.746	1.058
	Sex(1)	.167	.089	3.558	1	.059	1.182	.993	1.406
	MI(1)	-.229	.096	5.695	1	.017	.796	.659	.960
	HTN(1)	.022	.103	.045	1	.832	1.022	.836	1.250
	DM	.625	.089	49.680	1	.000	1.869	1.571	2.224
	OBS(1)	.155	.122	1.593	1	.207	1.167	.918	1.484
	FH(1)	-.236	.098	5.748	1	.017	.790	.651	.958
	hChol(1)	-.872	.084	106.829	1	.000	.418	.354	.493
	lHDL(1)	-.902	.079	128.909	1	.000	.406	.347	.474
	hLDL(1)	-.707	.105	45.656	1	.000	.493	.402	.605
	Age	-.013	.003	17.157	1	.000	.987	.981	.993
	BMI	.030	.011	8.102	1	.004	1.030	1.009	1.052
	Constant	.291	.468	.386	1	.534	1.338		

a. Variable(s) entered on step 1: rs2259820D, Sex, MI, HTN, DM, OBS, FH, hChol, lHDL, hLDL, Age, BMI.

Variables in the Equation

		B	S.E.	Wald	df	Sig.	Exp(B)	95% C.I.for EXP(B)	
								Lower	Upper
Step 1[a]	rs2464196A(1)	-.108	.054	4.038	1	.044	.898	.808	.997
	MI	.232	.067	12.004	1	.001	1.261	1.106	1.438
	Age	-.014	.002	36.899	1	.000	.987	.982	.991
	BMI	.026	.007	12.868	1	.000	1.026	1.012	1.041
	Sex(1)	.169	.062	7.461	1	.006	1.185	1.049	1.338
	HTN(1)	.042	.072	.338	1	.561	1.042	.906	1.199
	DM(1)	-.580	.061	89.181	1	.000	.560	.496	.632
	OBS(1)	.114	.085	1.767	1	.184	1.120	.948	1.324
	lHDL(1)	-.897	.056	260.735	1	.000	.408	.366	.455
	hLDL(1)	-.751	.073	105.360	1	.000	.472	.409	.545
	hChol(1)	-.863	.059	213.549	1	.000	.422	.376	.474
	Constant	.500	.303	2.720	1	.099	1.649		

a. Variable(s) entered on step 1: rs2464196A, MI, Age, BMI, Sex, HTN, DM, OBS, lHDL, hLDL, hChol.

Variables in the Equation

		B	S.E.	Wald	df	Sig.	Exp(B)	95% C.I.for EXP(B)	
								Lower	Upper
Step 1[a]	rs2464196R	.102	.089	1.311	1	.252	1.107	.930	1.317
	Sex(1)	.157	.088	3.192	1	.074	1.170	.985	1.390
	MI(1)	-.248	.095	6.812	1	.009	.781	.648	.940
	HTN(1)	.032	.101	.100	1	.752	1.033	.847	1.259
	DM	.617	.088	49.255	1	.000	1.854	1.560	2.203
	OBS(1)	.112	.121	.862	1	.353	1.119	.883	1.419
	FH(1)	-.275	.098	7.916	1	.005	.760	.627	.920
	hChol(1)	-.857	.084	105.116	1	.000	.425	.360	.500
	lHDL(1)	-.904	.079	131.772	1	.000	.405	.347	.473
	hLDL(1)	-.741	.104	51.244	1	.000	.476	.389	.584
	Age	-.012	.003	15.273	1	.000	.988	.982	.994
	BMI	.024	.010	5.504	1	.019	1.025	1.004	1.046
	Constant	.165	.445	.138	1	.711	1.180		

a. Variable(s) entered on step 1: rs2464196R, Sex, MI, HTN, DM, OBS, FH, hChol, lHDL, hLDL, Age, BMI.

Variables in the Equation

		B	S.E.	Wald	df	Sig.	Exp(B)	95% C.I.for EXP(B)	
								Lower	Upper
Step 1[a]	rs2464196D	.166	.083	3.933	1	.047	1.180	1.002	1.390
	Sex(1)	.157	.088	3.177	1	.075	1.170	.985	1.390
	MI(1)	-.254	.095	7.130	1	.008	.776	.644	.935
	HTN(1)	.037	.101	.130	1	.719	1.037	.850	1.265
	DM	.620	.088	49.639	1	.000	1.859	1.564	2.209
	OBS(1)	.111	.121	.847	1	.357	1.118	.882	1.417
	FH(1)	-.278	.098	8.119	1	.004	.757	.625	.917
	hChol(1)	-.858	.084	105.387	1	.000	.424	.360	.499
	lHDL(1)	-.904	.079	131.768	1	.000	.405	.347	.472
	hLDL(1)	-.746	.104	51.723	1	.000	.474	.387	.581
	Age	-.013	.003	15.500	1	.000	.988	.981	.994
	BMI	.024	.010	5.508	1	.019	1.025	1.004	1.046
	Constant	.021	.453	.002	1	.963	1.021		

a. Variable(s) entered on step 1: rs2464196D, Sex, MI, HTN, DM, OBS, FH, hChol, lHDL, hLDL, Age, BMI.

Variables in the Equation

		B	S.E.	Wald	df	Sig.	Exp(B)	95% C.I.for EXP(B)	
								Lower	Upper
Step 1[a]	rs2259816A	-.117	.055	4.516	1	.034	.889	.798	.991
	MI	.219	.069	10.229	1	.001	1.245	1.089	1.425
	Age	-.014	.002	35.215	1	.000	.987	.982	.991
	BMI	.031	.007	16.959	1	.000	1.031	1.016	1.046
	Sex(1)	.161	.064	6.408	1	.011	1.174	1.037	1.330
	HTN(1)	.041	.073	.322	1	.570	1.042	.904	1.202
	DM(1)	-.577	.063	84.976	1	.000	.562	.497	.635
	OBS(1)	.183	.087	4.381	1	.036	1.201	1.012	1.425
	lHDL(1)	-.890	.057	245.399	1	.000	.411	.367	.459
	hLDL(1)	-.719	.075	91.736	1	.000	.487	.420	.564
	hChol(1)	-.862	.060	204.048	1	.000	.422	.375	.475
	Constant	.427	.322	1.755	1	.185	1.533		

a. Variable(s) entered on step 1: rs2259816A, MI, Age, BMI, Sex, HTN, DM, OBS, lHDL, hLDL, hChol.

Variables in the Equation

		B	S.E.	Wald	df	Sig.	Exp(B)	95% C.I.for EXP(B)	
								Lower	Upper
Step 1[a]	rs2259816R	-.178	.082	4.739	1	.029	.837	.712	.982
	Sex(1)	.151	.090	2.809	1	.094	1.163	.975	1.387
	MI(1)	-.237	.097	5.947	1	.015	.789	.652	.955
	HTN(1)	.036	.103	.124	1	.725	1.037	.847	1.270
	DM	.610	.090	46.440	1	.000	1.841	1.545	2.194
	OBS(1)	.178	.124	2.059	1	.151	1.195	.937	1.523
	FH(1)	-.229	.100	5.255	1	.022	.796	.654	.967
	hChol(1)	-.858	.085	101.091	1	.000	.424	.359	.501
	lHDL(1)	-.896	.080	123.886	1	.000	.408	.349	.478
	hLDL(1)	-.713	.106	44.921	1	.000	.490	.398	.604
	Age	-.013	.003	15.343	1	.000	.987	.981	.994
	BMI	.029	.011	7.377	1	.007	1.029	1.008	1.051
	Constant	.310	.460	.454	1	.501	1.363		

a. Variable(s) entered on step 1: rs2259816R, Sex, MI, HTN, DM, OBS, FH, hChol, lHDL, hLDL, Age, BMI.

Variables in the Equation

		B	S.E.	Wald	df	Sig.	Exp(B)	95% C.I.for EXP(B)	
								Lower	Upper
Step 1[a]	rs2259816D	-.108	.097	1.228	1	.268	.898	.742	1.086
	Sex(1)	.150	.090	2.791	1	.095	1.162	.974	1.386
	MI(1)	-.233	.097	5.770	1	.016	.792	.655	.958
	HTN(1)	.034	.103	.107	1	.744	1.034	.845	1.266
	DM	.606	.089	45.857	1	.000	1.833	1.538	2.184
	OBS(1)	.182	.124	2.161	1	.142	1.200	.941	1.529
	FH(1)	-.230	.100	5.335	1	.021	.794	.653	.966
	hChol(1)	-.855	.085	100.433	1	.000	.425	.360	.503
	lHDL(1)	-.895	.080	123.793	1	.000	.409	.349	.478
	hLDL(1)	-.710	.106	44.589	1	.000	.492	.399	.606
	Age	-.013	.003	14.962	1	.000	.987	.981	.994
	BMI	.029	.011	7.549	1	.006	1.029	1.008	1.051
	Constant	.243	.479	.258	1	.611	1.275		

a. Variable(s) entered on step 1: rs2259816D, Sex, MI, HTN, DM, OBS, FH, hChol, lHDL, hLDL, Age, BMI.

rs1169310A

Variables in the Equation

		B	S.E.	Wald	df	Sig.	Exp(B)	95% C.I.for EXP(B)	
								Lower	Upper
Step 1[a]	rs1169310A	.140	.054	6.711	1	.010	1.150	1.035	1.278
	MI	.232	.067	12.044	1	.001	1.261	1.106	1.437
	Age	-.014	.002	38.741	1	.000	.986	.982	.991
	BMI	.027	.007	14.110	1	.000	1.028	1.013	1.042
	Sex(1)	.164	.062	7.037	1	.008	1.179	1.044	1.331
	HTN(1)	.040	.071	.310	1	.578	1.041	.905	1.197
	DM(1)	-.592	.061	93.750	1	.000	.553	.491	.624
	OBS(1)	.130	.085	2.318	1	.128	1.139	.963	1.345
	lHDL(1)	-.887	.055	256.581	1	.000	.412	.369	.459
	hLDL(1)	-.748	.073	104.880	1	.000	.473	.410	.546
	hChol(1)	-.856	.059	211.013	1	.000	.425	.378	.477
	Constant	.215	.308	.484	1	.486	1.239		

a. Variable(s) entered on step 1: rs1169310A, MI, Age, BMI, Sex, HTN, DM, OBS, lHDL, hLDL, hChol.

Variables in the Equation

		B	S.E.	Wald	df	Sig.	Exp(B)	95% C.I.for EXP(B)	
								Lower	Upper
Step 1[a]	rs1169310R	.145	.096	2.277	1	.131	1.156	.958	1.395
	Sex(1)	.152	.088	3.015	1	.082	1.165	.981	1.383
	MI(1)	-.246	.095	6.782	1	.009	.782	.649	.941
	HTN(1)	.032	.101	.098	1	.755	1.032	.847	1.258
	DM	.627	.087	51.410	1	.000	1.873	1.578	2.223
	OBS(1)	.131	.121	1.182	1	.277	1.140	.900	1.444
	FH(1)	-.266	.097	7.472	1	.006	.766	.633	.927
	hChol(1)	-.849	.083	103.676	1	.000	.428	.363	.504
	lHDL(1)	-.893	.078	129.468	1	.000	.409	.351	.478
	hLDL(1)	-.739	.103	51.000	1	.000	.478	.390	.585
	Age	-.013	.003	16.110	1	.000	.987	.981	.994
	BMI	.026	.010	6.204	1	.013	1.026	1.005	1.047
	Constant	.060	.444	.018	1	.893	1.062		

a. Variable(s) entered on step 1: rs1169310R, Sex, MI, HTN, DM, OBS, FH, hChol, lHDL, hLDL, Age, BMI.

Variables in the Equation

		B	S.E.	Wald	df	Sig.	Exp(B)	95% C.I.for EXP(B)	
								Lower	Upper
Step 1[a]	rs1169310D	.199	.080	6.262	1	.012	1.221	1.044	1.427
	Sex(1)	.152	.088	2.994	1	.084	1.164	.980	1.383
	MI(1)	-.251	.095	7.049	1	.008	.778	.646	.936
	HTN(1)	.035	.101	.120	1	.729	1.036	.849	1.263
	DM	.631	.088	51.942	1	.000	1.880	1.583	2.232
	OBS(1)	.126	.121	1.093	1	.296	1.135	.895	1.438
	FH(1)	-.266	.097	7.448	1	.006	.767	.633	.928
	hChol(1)	-.852	.083	104.405	1	.000	.426	.362	.502
	lHDL(1)	-.894	.079	129.635	1	.000	.409	.351	.477
	hLDL(1)	-.743	.104	51.509	1	.000	.476	.388	.583
	Age	-.013	.003	16.451	1	.000	.987	.981	.993
	BMI	.025	.010	6.006	1	.014	1.026	1.005	1.047
	Constant	-.070	.447	.025	1	.875	.932		

a. Variable(s) entered on step 1: rs1169310D, Sex, MI, HTN, DM, OBS, FH, hChol, lHDL, hLDL, Age, BMI.

rs1169313A vs hypertriglyceridemia

Variables in the Equation

		B	S.E.	Wald	df	Sig.	Exp(B)	95% C.I.for EXP(B)	
								Lower	Upper
Step 1[a]	rs1169313A	.136	.054	6.206	1	.013	1.145	1.029	1.274
	MI	.212	.067	9.878	1	.002	1.236	1.083	1.411
	Age	-.014	.002	38.100	1	.000	.986	.982	.991
	BMI	.028	.007	14.746	1	.000	1.029	1.014	1.043
	Sex(1)	.178	.062	8.122	1	.004	1.195	1.057	1.350
	HTN(1)	.038	.072	.273	1	.601	1.039	.901	1.197
	DM(1)	-.580	.062	88.388	1	.000	.560	.496	.632
	OBS(1)	.130	.086	2.294	1	.130	1.139	.962	1.349
	lHDL(1)	-.897	.056	257.608	1	.000	.408	.365	.455
	hLDL(1)	-.721	.073	96.539	1	.000	.486	.421	.562
	hChol(1)	-.892	.059	225.064	1	.000	.410	.365	.461
	Constant	.195	.312	.391	1	.532	1.215		

a. Variable(s) entered on step 1: rs1169313A, MI, Age, BMI, Sex, HTN, DM, OBS, lHDL, hLDL, hChol.

Variables in the Equation

		B	S.E.	Wald	df	Sig.	Exp(B)	95% C.I.for EXP(B)	
								Lower	Upper
Step 1[a]	rs1169313R	.113	.097	1.343	1	.246	1.119	.925	1.354
	Sex(1)	.167	.088	3.576	1	.059	1.182	.994	1.406
	MI(1)	-.224	.095	5.494	1	.019	.800	.663	.964
	HTN(1)	.030	.102	.089	1	.766	1.031	.844	1.260
	DM	.612	.088	48.053	1	.000	1.844	1.551	2.193
	OBS(1)	.132	.122	1.179	1	.277	1.142	.899	1.450
	FH(1)	-.247	.098	6.303	1	.012	.781	.644	.947
	hChol(1)	-.885	.084	110.843	1	.000	.413	.350	.487
	lHDL(1)	-.902	.079	129.805	1	.000	.406	.348	.474
	hLDL(1)	-.711	.104	46.906	1	.000	.491	.401	.602
	Age	-.013	.003	16.087	1	.000	.987	.981	.993
	BMI	.027	.010	6.612	1	.010	1.027	1.006	1.048
	Constant	.048	.451	.011	1	.916	1.049		

a. Variable(s) entered on step 1: rs1169313R, Sex, MI, HTN, DM, OBS, FH, hChol, lHDL, hLDL, Age, BMI.

Variables in the Equation

		B	S.E.	Wald	df	Sig.	Exp(B)	95% C.I.for EXP(B)	
								Lower	Upper
Step 1[a]	rs1169313D	.215	.080	7.142	1	.008	1.240	1.059	1.452
	Sex(1)	.166	.088	3.526	1	.060	1.181	.993	1.404
	MI(1)	-.231	.096	5.848	1	.016	.794	.658	.957
	HTN(1)	.033	.102	.104	1	.748	1.034	.845	1.263
	DM	.617	.088	48.686	1	.000	1.853	1.558	2.204
	OBS(1)	.127	.122	1.084	1	.298	1.135	.894	1.442
	FH(1)	-.248	.098	6.331	1	.012	.781	.644	.947
	hChol(1)	-.889	.084	111.597	1	.000	.411	.349	.485
	lHDL(1)	-.903	.079	129.906	1	.000	.405	.347	.474
	hLDL(1)	-.718	.104	47.632	1	.000	.488	.398	.598
	Age	-.013	.003	16.496	1	.000	.987	.981	.993
	BMI	.026	.010	6.359	1	.012	1.027	1.006	1.048
	Constant	-.138	.452	.093	1	.761	.871		

a. Variable(s) entered on step 1: rs1169313D, Sex, MI, HTN, DM, OBS, FH, hChol, lHDL, hLDL, Age, BMI.

HNF1a vs low High Density Lipoprotein-Cholesterol

Variables in the Equation

		B	S.E.	Wald	df	Sig.	Exp(B)	95% C.I.for EXP(B)	
								Lower	Upper
Step 1[a]	rs2393791A	-.032	.048	.436	1	.509	.969	.882	1.064
	MI(1)	-.423	.057	54.350	1	.000	.655	.585	.733
	Age	-.002	.002	.996	1	.318	.998	.994	1.002
	BMI	.013	.006	4.101	1	.043	1.013	1.000	1.026
	Sex(1)	.822	.053	237.540	1	.000	2.276	2.050	2.527
	HTN(1)	-.062	.061	1.013	1	.314	.940	.834	1.060
	DM(1)	-.284	.053	29.076	1	.000	.753	.679	.835
	OBS(1)	.114	.076	2.273	1	.132	1.121	.966	1.300
	hLDL(1)	.276	.073	14.380	1	.000	1.317	1.142	1.519
	hChol(1)	.244	.055	19.732	1	.000	1.276	1.146	1.422
	hTG	.878	.055	251.912	1	.000	2.405	2.158	2.681
	Constant	-1.410	.273	26.598	1	.000	.244		

a. Variable(s) entered on step 1: rs2393791A, MI, Age, BMI, Sex, HTN, DM, OBS, hLDL, hChol, hTG.

Variables in the Equation

		B	S.E.	Wald	df	Sig.	Exp(B)	95% C.I.for EXP(B)	
								Lower	Upper
Step 1[a]	rs2393791R(1)	.038	.089	.181	1	.670	1.039	.872	1.237
	Sex(1)	.826	.076	119.595	1	.000	2.285	1.971	2.650
	MI(1)	-.420	.081	26.672	1	.000	.657	.560	.771
	HTN(1)	-.060	.087	.477	1	.490	.942	.795	1.116
	DM	.273	.075	13.145	1	.000	1.313	1.133	1.522
	OBS(1)	.114	.107	1.133	1	.287	1.120	.909	1.382
	FH(1)	.087	.087	1.016	1	.313	1.091	.921	1.293
	hChol(1)	.241	.078	9.633	1	.002	1.273	1.093	1.483
	hTG(1)	-.880	.078	126.507	1	.000	.415	.356	.483
	hLDL(1)	.272	.103	6.998	1	.008	1.313	1.073	1.606
	Age	-.002	.003	.685	1	.408	.998	.993	1.003
	BMI	.013	.009	2.231	1	.135	1.014	.996	1.032
	Constant	-.951	.382	6.190	1	.013	.387		

a. Variable(s) entered on step 1: rs2393791R, Sex, MI, HTN, DM, OBS, FH, hChol, hTG, hLDL, Age, BMI.

Variables in the Equation

		B	S.E.	Wald	df	Sig.	Exp(B)	95% C.I.for EXP(B)	
								Lower	Upper
Step 1[a]	rs2393791D	-.041	.068	.365	1	.546	.960	.839	1.097
	Sex(1)	.826	.076	119.434	1	.000	2.284	1.969	2.648
	MI(1)	-.419	.081	26.580	1	.000	.658	.561	.771
	HTN(1)	-.060	.087	.488	1	.485	.941	.794	1.115
	DM	.272	.075	13.053	1	.000	1.312	1.132	1.521
	OBS(1)	.114	.107	1.141	1	.286	1.121	.909	1.382
	FH(1)	.087	.087	1.020	1	.313	1.091	.921	1.293
	hChol(1)	.243	.078	9.737	1	.002	1.275	1.094	1.485
	hTG(1)	-.882	.078	126.701	1	.000	.414	.355	.483
	hLDL(1)	.273	.103	7.031	1	.008	1.314	1.074	1.607
	Age	-.002	.003	.661	1	.416	.998	.993	1.003
	BMI	.013	.009	2.239	1	.135	1.014	.996	1.032
	Constant	-.855	.390	4.812	1	.028	.425		

a. Variable(s) entered on step 1: rs2393791D, Sex, MI, HTN, DM, OBS, FH, hChol, hTG, hLDL, Age, BMI.

Variables in the Equation

		B	S.E.	Wald	df	Sig.	Exp(B)	95% C.I.for EXP(B)	
								Lower	Upper
Step 1[a]	rs7310409A	-.066	.048	1.890	1	.169	.936	.852	1.028
	MI(1)	-.426	.057	55.498	1	.000	.653	.584	.731
	Age	-.002	.002	1.148	1	.284	.998	.994	1.002
	BMI	.012	.006	3.529	1	.060	1.012	.999	1.025
	Sex(1)	.803	.053	228.514	1	.000	2.233	2.012	2.478
	HTN(1)	-.053	.061	.757	1	.384	.948	.842	1.069
	DM(1)	-.275	.052	27.606	1	.000	.759	.685	.842
	OBS(1)	.104	.075	1.925	1	.165	1.110	.958	1.286
	hLDL(1)	.290	.072	16.070	1	.000	1.337	1.160	1.541
	hChol(1)	.243	.055	19.646	1	.000	1.275	1.145	1.419
	hTG	.881	.055	255.802	1	.000	2.414	2.167	2.690
	Constant	-1.324	.272	23.663	1	.000	.266		

a. Variable(s) entered on step 1: rs7310409A, MI, Age, BMI, Sex, HTN, DM, OBS, hLDL, hChol, hTG.

Variables in the Equation

		B	S.E.	Wald	df	Sig.	Exp(B)	95% C.I.for EXP(B)	
								Lower	Upper
Step 1[a]	rs7310409R	-.067	.089	.566	1	.452	.935	.785	1.114
	Sex(1)	.807	.075	115.080	1	.000	2.242	1.934	2.598
	MI(1)	-.423	.081	27.250	1	.000	.655	.559	.768
	HTN(1)	-.051	.086	.346	1	.557	.951	.803	1.126
	DM	.265	.075	12.529	1	.000	1.304	1.126	1.510
	OBS(1)	.103	.106	.947	1	.331	1.109	.900	1.366
	FH(1)	.083	.086	.921	1	.337	1.086	.917	1.287
	hChol(1)	.240	.078	9.545	1	.002	1.271	1.092	1.479
	hTG(1)	-.884	.078	128.292	1	.000	.413	.355	.482
	hLDL(1)	.287	.102	7.842	1	.005	1.332	1.090	1.629
	Age	-.002	.003	.757	1	.384	.998	.992	1.003
	BMI	.012	.009	1.909	1	.167	1.012	.995	1.030
	Constant	-.786	.387	4.111	1	.043	.456		

a. Variable(s) entered on step 1: rs7310409R, Sex, MI, HTN, DM, OBS, FH, hChol, hTG, hLDL, Age, BMI.

Variables in the Equation

		B	S.E.	Wald	df	Sig.	Exp(B)	95% C.I.for EXP(B)	
								Lower	Upper
Step 1[a]	rs7310409D	-.093	.068	1.879	1	.170	.911	.797	1.041
	Sex(1)	.806	.075	114.732	1	.000	2.239	1.932	2.595
	MI(1)	-.422	.081	27.199	1	.000	.656	.559	.768
	HTN(1)	-.054	.086	.385	1	.535	.948	.801	1.122
	DM	.263	.075	12.313	1	.000	1.301	1.123	1.506
	OBS(1)	.106	.106	.984	1	.321	1.111	.902	1.369
	FH(1)	.083	.086	.934	1	.334	1.087	.918	1.287
	hChol(1)	.243	.078	9.792	1	.002	1.275	1.095	1.484
	hTG(1)	-.886	.078	128.906	1	.000	.412	.354	.480
	hLDL(1)	.288	.102	7.916	1	.005	1.334	1.091	1.631
	Age	-.002	.003	.747	1	.387	.998	.992	1.003
	BMI	.013	.009	1.945	1	.163	1.013	.995	1.031
	Constant	-.718	.388	3.423	1	.064	.488		

a. Variable(s) entered on step 1: rs7310409D, Sex, MI, HTN, DM, OBS, FH, hChol, hTG, hLDL, Age, BMI.

Variables in the Equation

		B	S.E.	Wald	df	Sig.	Exp(B)	95% C.I.for EXP(B)	
								Lower	Upper
Step 1[a]	rs2259820A	.005	.047	.013	1	.911	1.005	.916	1.103
	MI(1)	-.422	.058	52.883	1	.000	.656	.585	.735
	Age	-.002	.002	.767	1	.381	.998	.995	1.002
	BMI	.011	.006	2.695	1	.101	1.011	.998	1.023
	Sex(1)	.789	.054	215.305	1	.000	2.200	1.980	2.445
	HTN(1)	-.064	.062	1.077	1	.299	.938	.831	1.059
	DM(1)	-.272	.053	26.145	1	.000	.762	.687	.846
	OBS(1)	.100	.076	1.716	1	.190	1.105	.952	1.283
	hLDL(1)	.307	.074	17.448	1	.000	1.360	1.177	1.570
	hChol(1)	.249	.056	19.989	1	.000	1.282	1.150	1.430
	hTG	.885	.056	250.757	1	.000	2.424	2.172	2.705
	Constant	-1.416	.281	25.383	1	.000	.243		

a. Variable(s) entered on step 1: rs2259820A, MI, Age, BMI, Sex, HTN, DM, OBS, hLDL, hChol, hTG.

Variables in the Equation

		B	S.E.	Wald	df	Sig.	Exp(B)	95% C.I.for EXP(B)	
								Lower	Upper
Step 1[a]	rs2259820R	.018	.073	.061	1	.806	1.018	.882	1.175
	Sex(1)	.791	.076	108.119	1	.000	2.206	1.900	2.561
	MI(1)	-.419	.082	25.953	1	.000	.658	.560	.773
	HTN(1)	-.063	.087	.524	1	.469	.939	.791	1.114
	DM	.263	.076	11.962	1	.001	1.300	1.121	1.509
	OBS(1)	.100	.108	.861	1	.353	1.105	.895	1.366
	FH(1)	.067	.087	.597	1	.440	1.070	.902	1.269
	hChol(1)	.247	.079	9.848	1	.002	1.280	1.097	1.494
	hTG(1)	-.888	.079	125.834	1	.000	.411	.352	.480
	hLDL(1)	.305	.104	8.612	1	.003	1.357	1.107	1.664
	Age	-.002	.003	.493	1	.482	.998	.993	1.003
	BMI	.011	.009	1.455	1	.228	1.011	.993	1.029
	Constant	-.864	.394	4.801	1	.028	.421		

a. Variable(s) entered on step 1: rs2259820R, Sex, MI, HTN, DM, OBS, FH, hChol, hTG, hLDL, Age, BMI.

Variables in the Equation

		B	S.E.	Wald	df	Sig.	Exp(B)	95% C.I.for EXP(B)	
								Lower	Upper
Step 1[a]	Sex(1)	.791	.076	108.138	1	.000	2.206	1.900	2.561
	MI(1)	-.419	.082	26.064	1	.000	.657	.560	.772
	HTN(1)	-.062	.087	.508	1	.476	.940	.792	1.115
	DM	.263	.076	12.025	1	.001	1.301	1.121	1.510
	OBS(1)	.100	.108	.850	1	.357	1.105	.894	1.365
	FH(1)	.068	.087	.602	1	.438	1.070	.902	1.269
	hChol(1)	.247	.079	9.832	1	.002	1.280	1.097	1.493
	hTG(1)	-.887	.079	125.731	1	.000	.412	.353	.481
	hLDL(1)	.304	.104	8.541	1	.003	1.355	1.105	1.662
	Age	-.002	.003	.498	1	.481	.998	.993	1.003
	BMI	.011	.009	1.443	1	.230	1.011	.993	1.029
	rs2259820D	-.008	.079	.011	1	.918	.992	.850	1.158
	Constant	-.824	.406	4.129	1	.042	.439		

a. Variable(s) entered on step 1: Sex, MI, HTN, DM, OBS, FH, hChol, hTG, hLDL, Age, BMI, rs2259820D.

Variables in the Equation

		B	S.E.	Wald	df	Sig.	Exp(B)	95% C.I.for EXP(B)	
								Lower	Upper
Step 1[a]	rs2464196A(1)	.006	.047	.015	1	.901	1.006	.917	1.103
	MI(1)	-.412	.057	51.415	1	.000	.663	.592	.741
	Age	-.001	.002	.591	1	.442	.999	.995	1.002
	BMI	.012	.006	3.802	1	.051	1.012	1.000	1.025
	Sex(1)	.803	.053	227.014	1	.000	2.232	2.011	2.478
	HTN(1)	-.049	.061	.650	1	.420	.952	.844	1.073
	DM(1)	-.287	.053	29.597	1	.000	.751	.677	.832
	OBS(1)	.118	.075	2.424	1	.119	1.125	.970	1.304
	hLDL(1)	.282	.073	15.021	1	.000	1.326	1.150	1.530
	hChol(1)	.246	.055	19.869	1	.000	1.278	1.148	1.424
	hTG	.886	.055	255.866	1	.000	2.426	2.176	2.704
	Constant	-1.468	.268	30.054	1	.000	.230		

a. Variable(s) entered on step 1: rs2464196A, MI, Age, BMI, Sex, HTN, DM, OBS, hLDL, hChol, hTG.

Variables in the Equation

		B	S.E.	Wald	df	Sig.	Exp(B)	95% C.I.for EXP(B)	
								Lower	Upper
Step 1[a]	rs2464196R	.010	.078	.018	1	.894	1.011	.867	1.178
	Sex(1)	.807	.075	114.238	1	.000	2.240	1.932	2.597
	MI(1)	-.408	.081	25.221	1	.000	.665	.567	.780
	HTN(1)	-.047	.086	.298	1	.585	.954	.805	1.130
	DM	.276	.075	13.395	1	.000	1.318	1.137	1.527
	OBS(1)	.117	.107	1.209	1	.272	1.125	.912	1.386
	FH(1)	.087	.087	1.007	1	.316	1.091	.920	1.293
	hChol(1)	.243	.078	9.743	1	.002	1.276	1.095	1.486
	hTG(1)	-.889	.078	128.502	1	.000	.411	.352	.479
	hLDL(1)	.279	.103	7.297	1	.007	1.321	1.079	1.617
	Age	-.002	.003	.434	1	.510	.998	.993	1.003
	BMI	.013	.009	2.066	1	.151	1.013	.995	1.031
	Constant	-.938	.387	5.882	1	.015	.392		

a. Variable(s) entered on step 1: rs2464196R, Sex, MI, HTN, DM, OBS, FH, hChol, hTG, hLDL, Age, BMI.

Variables in the Equation

		B	S.E.	Wald	df	Sig.	Exp(B)	95% C.I.for EXP(B)	
								Lower	Upper
Step 1[a]	rs2464196D	-.021	.072	.083	1	.774	.979	.850	1.129
	Sex(1)	.807	.075	114.258	1	.000	2.240	1.932	2.597
	MI(1)	-.407	.081	25.099	1	.000	.665	.567	.780
	HTN(1)	-.048	.087	.313	1	.576	.953	.804	1.129
	DM	.275	.075	13.317	1	.000	1.317	1.136	1.526
	OBS(1)	.118	.107	1.224	1	.269	1.125	.913	1.387
	FH(1)	.087	.087	1.000	1	.317	1.091	.920	1.293
	hChol(1)	.244	.078	9.759	1	.002	1.276	1.095	1.487
	hTG(1)	-.890	.078	128.654	1	.000	.411	.352	.479
	hLDL(1)	.280	.103	7.378	1	.007	1.323	1.081	1.620
	Age	-.002	.003	.429	1	.513	.998	.993	1.004
	BMI	.013	.009	2.083	1	.149	1.013	.995	1.031
	Constant	-.892	.392	5.171	1	.023	.410		

a. Variable(s) entered on step 1: rs2464196D, Sex, MI, HTN, DM, OBS, FH, hChol, hTG, hLDL, Age, BMI.

Variables in the Equation

		B	S.E.	Wald	df	Sig.	Exp(B)	95% C.I.for EXP(B)	
								Lower	Upper
Step 1[a]	rs2259816A	.019	.048	.151	1	.698	1.019	.927	1.121
	MI(1)	-.427	.059	52.899	1	.000	.652	.581	.732
	Age	-.002	.002	1.145	1	.285	.998	.994	1.002
	BMI	.012	.006	3.544	1	.060	1.012	.999	1.025
	Sex(1)	.830	.055	231.285	1	.000	2.292	2.060	2.551
	HTN(1)	-.062	.062	.977	1	.323	.940	.832	1.062
	DM(1)	-.271	.054	25.587	1	.000	.763	.687	.847
	OBS(1)	.100	.077	1.663	1	.197	1.105	.950	1.285
	hLDL(1)	.251	.075	11.350	1	.001	1.286	1.111	1.488
	hChol(1)	.247	.056	19.324	1	.000	1.280	1.147	1.429
	hTG	.879	.057	240.813	1	.000	2.408	2.155	2.690
	Constant	-1.422	.284	25.041	1	.000	.241		

a. Variable(s) entered on step 1: rs2259816A, MI, Age, BMI, Sex, HTN, DM, OBS, hLDL, hChol, hTG.

Variables in the Equation

		B	S.E.	Wald	df	Sig.	Exp(B)	95% C.I.for EXP(B)	
								Lower	Upper
Step 1[a]	rs2259816A	.033	.071	.209	1	.648	1.033	.899	1.188
	Sex(1)	.833	.077	116.356	1	.000	2.301	1.978	2.677
	MI(1)	-.423	.083	25.816	1	.000	.655	.557	.771
	HTN(1)	-.060	.088	.468	1	.494	.941	.792	1.119
	DM	.259	.077	11.455	1	.001	1.296	1.115	1.506
	OBS(1)	.101	.109	.851	1	.356	1.106	.893	1.370
	FH(1)	.088	.088	1.000	1	.317	1.092	.919	1.299
	hChol(1)	.245	.080	9.504	1	.002	1.278	1.093	1.493
	hTG(1)	-.882	.080	121.067	1	.000	.414	.354	.484
	hLDL(1)	.248	.106	5.520	1	.019	1.282	1.042	1.576
	Age	-.002	.003	.747	1	.387	.998	.992	1.003
	BMI	.013	.009	1.970	1	.160	1.013	.995	1.031
	Constant	-.895	.398	5.046	1	.025	.409		

a. Variable(s) entered on step 1: rs2259816A, Sex, MI, HTN, DM, OBS, FH, hChol, hTG, hLDL, Age, BMI.

Variables in the Equation

		B	S.E.	Wald	df	Sig.	Exp(B)	95% C.I.for EXP(B)	
								Lower	Upper
Step 1[a]	rs2259816D	.009	.086	.011	1	.915	1.009	.852	1.195
	Sex(1)	.834	.077	116.411	1	.000	2.301	1.978	2.678
	MI(1)	-.424	.083	25.942	1	.000	.655	.556	.771
	HTN(1)	-.060	.088	.457	1	.499	.942	.793	1.120
	DM	.260	.077	11.523	1	.001	1.297	1.116	1.507
	OBS(1)	.100	.109	.838	1	.360	1.105	.892	1.369
	FH(1)	.089	.088	1.010	1	.315	1.093	.919	1.300
	hChol(1)	.245	.079	9.463	1	.002	1.277	1.093	1.492
	hTG(1)	-.881	.080	120.879	1	.000	.414	.354	.485
	hLDL(1)	.247	.106	5.471	1	.019	1.280	1.041	1.574
	Age	-.002	.003	.761	1	.383	.998	.992	1.003
	BMI	.013	.009	1.951	1	.163	1.013	.995	1.031
	Constant	-.864	.416	4.325	1	.038	.421		

a. Variable(s) entered on step 1: rs2259816D, Sex, MI, HTN, DM, OBS, FH, hChol, hTG, hLDL, Age, BMI.

Variables in the Equation

		B	S.E.	Wald	df	Sig.	Exp(B)	95% C.I.for EXP(B)	
								Lower	Upper
Step 1[a]	rs1169310A	-.025	.047	.279	1	.597	.975	.889	1.070
	MI(1)	-.419	.057	53.472	1	.000	.658	.588	.736
	Age	-.002	.002	1.119	1	.290	.998	.994	1.002
	BMI	.012	.006	3.781	1	.052	1.012	1.000	1.025
	Sex(1)	.818	.053	235.822	1	.000	2.266	2.041	2.515
	HTN(1)	-.052	.061	.739	1	.390	.949	.842	1.069
	DM(1)	-.283	.052	29.066	1	.000	.754	.680	.835
	OBS(1)	.104	.075	1.896	1	.168	1.109	.957	1.286
	hLDL(1)	.291	.073	15.967	1	.000	1.337	1.160	1.542
	hChol(1)	.240	.055	19.072	1	.000	1.271	1.141	1.416
	hTG	.877	.055	251.735	1	.000	2.403	2.156	2.677
	Constant	-1.397	.272	26.426	1	.000	.247		

a. Variable(s) entered on step 1: rs1169310A, MI, Age, BMI, Sex, HTN, DM, OBS, hLDL, hChol, hTG.

Variables in the Equation

		B	S.E.	Wald	df	Sig.	Exp(B)	95% C.I.for EXP(B)	
								Lower	Upper
Step 1[a]	rs1169310R	-.007	.085	.007	1	.933	.993	.840	1.174
	Sex(1)	.821	.075	118.515	1	.000	2.273	1.960	2.635
	MI(1)	-.416	.081	26.368	1	.000	.659	.563	.773
	HTN(1)	-.051	.086	.344	1	.557	.951	.803	1.126
	DM	.273	.075	13.243	1	.000	1.314	1.134	1.522
	OBS(1)	.103	.107	.939	1	.332	1.109	.900	1.366
	FH(1)	.078	.087	.816	1	.366	1.081	.913	1.281
	hChol(1)	.238	.078	9.347	1	.002	1.268	1.089	1.477
	hTG(1)	-.879	.078	126.256	1	.000	.415	.356	.484
	hLDL(1)	.287	.103	7.769	1	.005	1.332	1.089	1.630
	Age	-.002	.003	.728	1	.393	.998	.993	1.003
	BMI	.013	.009	2.027	1	.154	1.013	.995	1.031
	Constant	-.881	.386	5.200	1	.023	.414		

a. Variable(s) entered on step 1: rs1169310R, Sex, MI, HTN, DM, OBS, FH, hChol, hTG, hLDL, Age, BMI.

Variables in the Equation

		B	S.E.	Wald	df	Sig.	Exp(B)	95% C.I.for EXP(B)	
								Lower	Upper
Step 1[a]	rs1169310D	-.047	.069	.471	1	.493	.954	.833	1.092
	Sex(1)	.821	.075	118.560	1	.000	2.273	1.961	2.635
	MI(1)	-.415	.081	26.156	1	.000	.660	.563	.774
	HTN(1)	-.052	.086	.364	1	.546	.949	.802	1.124
	DM	.272	.075	13.113	1	.000	1.312	1.133	1.520
	OBS(1)	.105	.107	.965	1	.326	1.110	.901	1.368
	FH(1)	.078	.087	.811	1	.368	1.081	.912	1.281
	hChol(1)	.239	.078	9.413	1	.002	1.270	1.090	1.479
	hTG(1)	-.881	.078	126.664	1	.000	.414	.356	.483
	hLDL(1)	.289	.103	7.885	1	.005	1.335	1.091	1.634
	Age	-.002	.003	.711	1	.399	.998	.993	1.003
	BMI	.013	.009	2.072	1	.150	1.013	.995	1.031
	Constant	-.819	.388	4.455	1	.035	.441		

a. Variable(s) entered on step 1: rs1169310D, Sex, MI, HTN, DM, OBS, FH, hChol, hTG, hLDL, Age, BMI.

Variables in the Equation

		B	S.E.	Wald	df	Sig.	Exp(B)	95% C.I.for EXP(B)	
								Lower	Upper
Step 1[a]	rs1169313A	-.016	.048	.115	1	.734	.984	.896	1.081
	MI(1)	-.444	.058	58.977	1	.000	.642	.573	.719
	Age	-.002	.002	1.487	1	.223	.998	.994	1.001
	BMI	.011	.006	3.232	1	.072	1.012	.999	1.024
	Sex(1)	.785	.054	215.082	1	.000	2.192	1.974	2.435
	HTN(1)	-.058	.062	.882	1	.348	.944	.836	1.065
	DM(1)	-.284	.053	28.805	1	.000	.753	.679	.835
	OBS(1)	.105	.076	1.903	1	.168	1.110	.957	1.289
	hLDL(1)	.290	.073	15.830	1	.000	1.337	1.159	1.542
	hChol(1)	.241	.055	18.904	1	.000	1.272	1.141	1.418
	hTG	.886	.056	252.754	1	.000	2.426	2.175	2.706
	Constant	-1.335	.275	23.634	1	.000	.263		

a. Variable(s) entered on step 1: rs1169313A, MI, Age, BMI, Sex, HTN, DM, OBS, hLDL, hChol, hTG.

Variables in the Equation

	B	S.E.	Wald	df	Sig.	Exp(B)	95% C.I.for EXP(B)

								Lower	Upper
Step 1[a]	rs1169313R	.002	.086	.001	1	.978	1.002	.847	1.187
	Sex(1)	.788	.076	108.029	1	.000	2.198	1.895	2.550
	MI(1)	-.442	.082	29.168	1	.000	.643	.548	.755
	HTN(1)	-.056	.087	.417	1	.519	.945	.797	1.121
	DM	.276	.076	13.266	1	.000	1.317	1.136	1.528
	OBS(1)	.105	.107	.948	1	.330	1.110	.899	1.371
	FH(1)	.068	.087	.618	1	.432	1.071	.903	1.270
	hChol(1)	.239	.078	9.299	1	.002	1.270	1.089	1.481
	hTG(1)	-.888	.079	126.703	1	.000	.412	.353	.480
	hLDL(1)	.287	.103	7.732	1	.005	1.332	1.088	1.631
	Age	-.003	.003	.902	1	.342	.997	.992	1.003
	BMI	.012	.009	1.726	1	.189	1.012	.994	1.030
	Constant	-.804	.391	4.227	1	.040	.448		

a. Variable(s) entered on step 1: rs1169313R, Sex, MI, HTN, DM, OBS, FH, hChol, hTG, hLDL, Age, BMI.

Variables in the Equation

		B	S.E.	Wald	df	Sig.	Exp(B)	95% C.I.for EXP(B)	
								Lower	Upper
Step 1[a]	rs1169313D	-.035	.070	.258	1	.612	.965	.842	1.107
	Sex(1)	.788	.076	108.071	1	.000	2.199	1.895	2.551
	MI(1)	-.440	.082	28.975	1	.000	.644	.548	.756
	HTN(1)	-.057	.087	.434	1	.510	.944	.796	1.120
	DM	.275	.076	13.172	1	.000	1.316	1.135	1.526
	OBS(1)	.105	.107	.961	1	.327	1.111	.900	1.372
	FH(1)	.068	.087	.616	1	.433	1.071	.903	1.270
	hChol(1)	.240	.078	9.342	1	.002	1.271	1.090	1.482
	hTG(1)	-.890	.079	126.984	1	.000	.411	.352	.480
	hLDL(1)	.289	.103	7.825	1	.005	1.335	1.090	1.634
	Age	-.003	.003	.888	1	.346	.997	.992	1.003
	BMI	.012	.009	1.754	1	.185	1.012	.994	1.030
	Constant	-.747	.392	3.638	1	.056	.474		

a. Variable(s) entered on step 1: rs1169313D, Sex, MI, HTN, DM, OBS, FH, hChol, hTG, hLDL, Age, BMI.

HNF1a vs high Low Density Lipoprotein -Cholesterol

Variables in the Equation

		B	S.E.	Wald	df	Sig.	Exp(B)	95% C.I.for EXP(B)	
								Lower	Upper
Step 1[a]	rs2393791A	-.115	.071	2.608	1	.106	.892	.776	1.025
	MI(1)	.383	.084	20.749	1	.000	1.467	1.244	1.730
	Age	-.002	.003	.490	1	.484	.998	.992	1.004
	BMI	-.026	.010	7.080	1	.008	.974	.955	.993
	Sex(1)	-.094	.078	1.457	1	.227	.910	.782	1.060
	HTN(1)	.127	.092	1.925	1	.165	1.136	.949	1.360
	DM(1)	.168	.077	4.751	1	.029	1.183	1.017	1.376
	OBS(1)	-.034	.112	.093	1	.761	.966	.775	1.204
	hChol(1)	-2.419	.086	797.192	1	.000	.089	.075	.105
	hTG	.682	.073	86.355	1	.000	1.977	1.712	2.283
	lHDL(1)	.265	.073	13.128	1	.000	1.303	1.129	1.504
	Constant	-.204	.414	.243	1	.622	.816		

a. Variable(s) entered on step 1: rs2393791A, MI, Age, BMI, Sex, HTN, DM, OBS, hChol, hTG, lHDL.

Variables in the Equation

		B	S.E.	Wald	df	Sig.	Exp(B)	95% C.I.for EXP(B)	
								Lower	Upper
Step 1[a]	rs2393791R(1)	.137	.133	1.059	1	.303	1.147	.883	1.489
	Sex(1)	-.104	.110	.894	1	.344	.901	.725	1.119
	MI(1)	.366	.119	9.437	1	.002	1.442	1.142	1.821
	HTN(1)	.122	.130	.880	1	.348	1.129	.876	1.457
	DM	-.139	.110	1.586	1	.208	.870	.701	1.080
	OBS(1)	-.034	.159	.046	1	.831	.967	.708	1.320
	FH(1)	-.208	.123	2.883	1	.090	.812	.639	1.033
	hChol(1)	-2.410	.121	396.182	1	.000	.090	.071	.114
	hTG(1)	-.673	.104	42.002	1	.000	.510	.416	.625
	lHDL(1)	.258	.103	6.193	1	.013	1.294	1.056	1.585
	Age	-.001	.004	.075	1	.784	.999	.991	1.007
	BMI	-.028	.014	4.013	1	.045	.972	.945	.999
	Constant	.534	.581	.846	1	.358	1.706		

a. Variable(s) entered on step 1: rs2393791R, Sex, MI, HTN, DM, OBS, FH, hChol, hTG, lHDL, Age, BMI.

Variables in the Equation

		B	S.E.	Wald	df	Sig.	Exp(B)	95% C.I.for EXP(B)	
								Lower	Upper
Step 1[a]	rs2393791D	-.151	.099	2.300	1	.129	.860	.708	1.045
	Sex(1)	-.110	.110	.986	1	.321	.896	.722	1.113
	MI(1)	.372	.119	9.734	1	.002	1.450	1.148	1.832
	HTN(1)	.120	.130	.854	1	.355	1.127	.874	1.454
	DM	-.142	.110	1.656	1	.198	.868	.699	1.077
	OBS(1)	-.034	.159	.045	1	.833	.967	.708	1.321
	FH(1)	-.206	.123	2.808	1	.094	.814	.640	1.035
	hChol(1)	-2.407	.121	394.696	1	.000	.090	.071	.114
	hTG(1)	-.675	.104	42.169	1	.000	.509	.415	.624
	lHDL(1)	.256	.103	6.136	1	.013	1.292	1.055	1.583
	Age	-.001	.004	.062	1	.803	.999	.991	1.007
	BMI	-.028	.014	3.987	1	.046	.972	.946	.999
	Constant	.885	.592	2.231	1	.135	2.423		

a. Variable(s) entered on step 1: rs2393791D, Sex, MI, HTN, DM, OBS, FH, hChol, hTG, lHDL, Age, BMI.

Variables in the Equation

		B	S.E.	Wald	df	Sig.	Exp(B)	95% C.I.for EXP(B)	
								Lower	Upper
Step 1[a]	rs7310409A	-.113	.071	2.527	1	.112	.894	.778	1.027
	MI(1)	.379	.084	20.412	1	.000	1.461	1.239	1.722
	Age	-.002	.003	.495	1	.482	.998	.992	1.004
	BMI	-.027	.010	7.185	1	.007	.974	.955	.993
	Sex(1)	-.091	.077	1.383	1	.240	.913	.784	1.063
	HTN(1)	.130	.092	1.999	1	.157	1.138	.951	1.362
	DM(1)	.166	.077	4.655	1	.031	1.180	1.015	1.372
	OBS(1)	-.040	.112	.128	1	.720	.961	.772	1.196
	hChol(1)	-2.436	.086	810.556	1	.000	.088	.074	.103
	hTG	.670	.073	83.764	1	.000	1.953	1.693	2.255
	lHDL(1)	.281	.073	14.904	1	.000	1.325	1.148	1.528
	Constant	-.196	.412	.227	1	.634	.822		

a. Variable(s) entered on step 1: rs7310409A, MI, Age, BMI, Sex, HTN, DM, OBS, hChol, hTG, lHDL.

Variables in the Equation

		B	S.E.	Wald	df	Sig.	Exp(B)	95% C.I.for EXP(B)	
								Lower	Upper
Step 1[a]	rs7310409R	-.155	.135	1.330	1	.249	.856	.657	1.115
	Sex(1)	-.099	.110	.810	1	.368	.906	.730	1.124
	MI(1)	.364	.119	9.390	1	.002	1.439	1.140	1.816
	HTN(1)	.124	.130	.918	1	.338	1.132	.878	1.459
	DM	-.139	.110	1.601	1	.206	.870	.701	1.079
	OBS(1)	-.040	.158	.063	1	.801	.961	.705	1.310
	FH(1)	-.195	.122	2.544	1	.111	.823	.647	1.046
	hChol(1)	-2.427	.121	402.920	1	.000	.088	.070	.112
	hTG(1)	-.662	.104	40.830	1	.000	.516	.421	.632
	lHDL(1)	.274	.103	7.067	1	.008	1.315	1.075	1.610
	Age	-.001	.004	.076	1	.782	.999	.991	1.007
	BMI	-.028	.014	4.023	1	.045	.972	.946	.999
	Constant	.810	.589	1.890	1	.169	2.248		

a. Variable(s) entered on step 1: rs7310409R, Sex, MI, HTN, DM, OBS, FH, hChol, hTG, lHDL, Age, BMI.

Variables in the Equation

		B	S.E.	Wald	df	Sig.	Exp(B)	95% C.I.for EXP(B)	
								Lower	Upper
Step 1[a]	rs7310409D	-.137	.099	1.920	1	.166	.872	.718	1.058
	Sex(1)	-.107	.110	.940	1	.332	.899	.725	1.115
	MI(1)	.367	.119	9.522	1	.002	1.443	1.143	1.821
	HTN(1)	.122	.130	.886	1	.347	1.130	.876	1.456
	DM	-.140	.110	1.631	1	.202	.869	.700	1.078
	OBS(1)	-.039	.158	.061	1	.804	.962	.705	1.311
	FH(1)	-.190	.122	2.413	1	.120	.827	.650	1.051
	hChol(1)	-2.424	.121	401.363	1	.000	.089	.070	.112
	hTG(1)	-.663	.104	40.932	1	.000	.515	.420	.631
	lHDL(1)	.275	.103	7.100	1	.008	1.316	1.075	1.611
	Age	-.001	.004	.082	1	.775	.999	.991	1.007
	BMI	-.028	.014	3.999	1	.046	.972	.946	.999
	Constant	.850	.590	2.076	1	.150	2.340		

a. Variable(s) entered on step 1: rs7310409D, Sex, MI, HTN, DM, OBS, FH, hChol, hTG, lHDL, Age, BMI.

Variables in the Equation

		B	S.E.	Wald	df	Sig.	Exp(B)	95% C.I.for EXP(B)	
								Lower	Upper
Step 1[a]	rs2259820A	.203	.070	8.435	1	.004	1.226	1.068	1.406
	MI(1)	.356	.085	17.489	1	.000	1.428	1.208	1.688
	Age	-.002	.003	.678	1	.410	.998	.992	1.003
	BMI	-.026	.010	6.390	1	.011	.975	.956	.994
	Sex(1)	-.124	.078	2.502	1	.114	.883	.757	1.030
	HTN(1)	.114	.093	1.495	1	.221	1.120	.934	1.344
	DM(1)	.171	.078	4.807	1	.028	1.187	1.018	1.384
	OBS(1)	-.030	.114	.071	1	.789	.970	.776	1.212
	hChol(1)	-2.455	.087	796.972	1	.000	.086	.072	.102
	hTG	.661	.074	78.862	1	.000	1.936	1.673	2.240
	lHDL(1)	.300	.074	16.506	1	.000	1.351	1.168	1.561
	Constant	-.648	.426	2.311	1	.128	.523		

a. Variable(s) entered on step 1: rs2259820A, MI, Age, BMI, Sex, HTN, DM, OBS, hChol, hTG, lHDL.

Variables in the Equation

		B	S.E.	Wald	df	Sig.	Exp(B)	95% C.I.for EXP(B)	
								Lower	Upper
Step 1[a]	rs2259820R	.266	.105	6.454	1	.011	1.305	1.063	1.602
	Sex(1)	-.137	.111	1.515	1	.218	.872	.701	1.085
	MI(1)	.349	.121	8.344	1	.004	1.418	1.119	1.796
	HTN(1)	.101	.131	.596	1	.440	1.107	.855	1.432
	DM	-.149	.112	1.764	1	.184	.862	.692	1.073
	OBS(1)	-.031	.161	.036	1	.849	.970	.707	1.330
	FH(1)	-.183	.124	2.182	1	.140	.832	.653	1.062
	hChol(1)	-2.442	.123	394.232	1	.000	.087	.068	.111
	hTG(1)	-.656	.105	38.700	1	.000	.519	.422	.638
	lHDL(1)	.292	.105	7.759	1	.005	1.339	1.090	1.644
	Age	-.001	.004	.118	1	.731	.999	.991	1.007
	BMI	-.027	.014	3.616	1	.057	.973	.946	1.001
	Constant	.290	.598	.235	1	.628	1.337		

a. Variable(s) entered on step 1: rs2259820R, Sex, MI, HTN, DM, OBS, FH, hChol, hTG, lHDL, Age, BMI.

Variables in the Equation

		B	S.E.	Wald	df	Sig.	Exp(B)	95% C.I.for EXP(B)	
								Lower	Upper
Step 1[a]	rs2259820D	.252	.121	4.348	1	.037	1.286	1.015	1.629
	Sex(1)	-.134	.111	1.456	1	.228	.874	.703	1.087
	MI(1)	.338	.121	7.859	1	.005	1.403	1.107	1.777
	HTN(1)	.109	.131	.689	1	.407	1.115	.862	1.442
	DM	-.149	.112	1.763	1	.184	.862	.692	1.073
	OBS(1)	-.028	.161	.030	1	.863	.973	.709	1.333
	FH(1)	-.193	.124	2.409	1	.121	.825	.647	1.052
	hChol(1)	-2.446	.123	395.787	1	.000	.087	.068	.110
	hTG(1)	-.655	.105	38.612	1	.000	.520	.423	.639
	lHDL(1)	.297	.105	8.025	1	.005	1.345	1.096	1.652
	Age	-.002	.004	.142	1	.706	.998	.990	1.007
	BMI	-.027	.014	3.477	1	.062	.974	.946	1.001
	Constant	.188	.621	.091	1	.763	1.207		

a. Variable(s) entered on step 1: rs2259820D, Sex, MI, HTN, DM, OBS, FH, hChol, hTG, lHDL, Age, BMI.

Variables in the Equation

		B	S.E.	Wald	df	Sig.	Exp(B)	95% C.I.for EXP(B)	
								Lower	Upper
Step 1[a]	rs2464196A(1)	.198	.069	8.157	1	.004	1.220	1.064	1.398
	MI(1)	.401	.084	22.624	1	.000	1.493	1.266	1.762
	Age	-.002	.003	.398	1	.528	.998	.993	1.004
	BMI	-.025	.010	6.422	1	.011	.975	.956	.994
	Sex(1)	-.084	.078	1.167	1	.280	.919	.789	1.071
	HTN(1)	.110	.092	1.431	1	.232	1.117	.932	1.338
	DM(1)	.182	.078	5.483	1	.019	1.199	1.030	1.396
	OBS(1)	-.042	.112	.142	1	.706	.959	.769	1.195
	hChol(1)	-2.435	.086	799.991	1	.000	.088	.074	.104
	hTG	.695	.074	89.234	1	.000	2.005	1.735	2.316
	lHDL(1)	.272	.073	13.752	1	.000	1.313	1.137	1.515
	Constant	-.530	.405	1.711	1	.191	.588		

a. Variable(s) entered on step 1: rs2464196A, MI, Age, BMI, Sex, HTN, DM, OBS, hChol, hTG, lHDL.

Variables in the Equation

		B	S.E.	Wald	df	Sig.	Exp(B)	95% C.I.for EXP(B)	
								Lower	Upper
Step 1[a]	rs2464196R	-.260	.120	4.701	1	.030	.771	.609	.975
	Sex(1)	-.095	.111	.736	1	.391	.909	.732	1.130
	MI(1)	.383	.119	10.286	1	.001	1.467	1.161	1.854
	HTN(1)	.107	.130	.678	1	.410	1.113	.862	1.437
	DM	-.158	.111	2.029	1	.154	.854	.687	1.061
	OBS(1)	-.039	.159	.060	1	.806	.962	.704	1.313
	FH(1)	-.189	.124	2.337	1	.126	.828	.650	1.055
	hChol(1)	-2.426	.122	397.416	1	.000	.088	.070	.112
	hTG(1)	-.689	.104	43.617	1	.000	.502	.409	.616
	lHDL(1)	.266	.104	6.580	1	.010	1.305	1.065	1.600
	Age	-.001	.004	.059	1	.809	.999	.991	1.007
	BMI	-.026	.014	3.479	1	.062	.974	.947	1.001
	Constant	.904	.588	2.360	1	.124	2.470		

a. Variable(s) entered on step 1: rs2464196R, Sex, MI, HTN, DM, OBS, FH, hChol, hTG, lHDL, Age, BMI.

Variables in the Equation

		B	S.E.	Wald	df	Sig.	Exp(B)	95% C.I.for EXP(B)	
								Lower	Upper
Step 1[a]	rs2464196D	-.250	.104	5.778	1	.016	.779	.635	.955
	Sex(1)	-.098	.111	.781	1	.377	.907	.730	1.126
	MI(1)	.393	.120	10.788	1	.001	1.481	1.172	1.872
	HTN(1)	.099	.131	.570	1	.450	1.104	.854	1.425
	DM	-.159	.111	2.059	1	.151	.853	.686	1.060
	OBS(1)	-.045	.159	.081	1	.776	.956	.700	1.305
	FH(1)	-.180	.124	2.127	1	.145	.835	.655	1.064
	hChol(1)	-2.424	.122	396.245	1	.000	.089	.070	.112
	hTG(1)	-.688	.104	43.485	1	.000	.502	.410	.617
	lHDL(1)	.263	.104	6.408	1	.011	1.301	1.061	1.595
	Age	-.001	.004	.053	1	.818	.999	.991	1.007
	BMI	-.027	.014	3.675	1	.055	.973	.947	1.001
	Constant	1.026	.599	2.937	1	.087	2.789		

a. Variable(s) entered on step 1: rs2464196D, Sex, MI, HTN, DM, OBS, FH, hChol, hTG, lHDL, Age, BMI.

Variables in the Equation

		B	S.E.	Wald	df	Sig.	Exp(B)	95% C.I.for EXP(B)	
								Lower	Upper
Step 1[a]	rs2259816A	.164	.072	5.187	1	.023	1.179	1.023	1.358
	MI(1)	.388	.087	20.055	1	.000	1.474	1.244	1.747
	Age	-.003	.003	.924	1	.337	.997	.991	1.003
	BMI	-.028	.010	7.694	1	.006	.972	.953	.992
	Sex(1)	-.088	.080	1.213	1	.271	.915	.782	1.071
	HTN(1)	.142	.094	2.299	1	.129	1.153	.959	1.386
	DM(1)	.217	.079	7.520	1	.006	1.242	1.064	1.451
	OBS(1)	-.090	.115	.606	1	.436	.914	.729	1.146
	hChol(1)	-2.481	.089	772.775	1	.000	.084	.070	.100
	hTG	.661	.076	76.451	1	.000	1.937	1.671	2.247
	lHDL(1)	.249	.075	11.001	1	.001	1.283	1.107	1.487
	Constant	-.495	.434	1.301	1	.254	.610		

a. Variable(s) entered on step 1: rs2259816A, MI, Age, BMI, Sex, HTN, DM, OBS, hChol, hTG, lHDL.

Variables in the Equation

		B	S.E.	Wald	df	Sig.	Exp(B)	95% C.I.for EXP(B)	
								Lower	Upper
Step 1[a]	rs2259816R	.187	.104	3.240	1	.072	1.205	.984	1.477
	Sex(1)	-.106	.114	.860	1	.354	.900	.720	1.125
	MI(1)	.373	.123	9.218	1	.002	1.452	1.141	1.847
	HTN(1)	.132	.133	.996	1	.318	1.142	.880	1.480
	DM	-.186	.113	2.708	1	.100	.830	.665	1.036
	OBS(1)	-.094	.163	.327	1	.567	.911	.661	1.255
	FH(1)	-.236	.126	3.522	1	.061	.790	.618	1.011
	hChol(1)	-2.466	.126	382.239	1	.000	.085	.066	.109
	hTG(1)	-.652	.107	37.000	1	.000	.521	.422	.643
	lHDL(1)	.239	.107	5.018	1	.025	1.269	1.030	1.564
	Age	-.002	.004	.162	1	.687	.998	.990	1.007
	BMI	-.031	.015	4.469	1	.035	.970	.942	.998
	Constant	.582	.610	.912	1	.340	1.790		

a. Variable(s) entered on step 1: rs2259816R, Sex, MI, HTN, DM, OBS, FH, hChol, hTG, lHDL, Age, BMI.

Variables in the Equation

		B	S.E.	Wald	df	Sig.	Exp(B)	95% C.I.for EXP(B)	
								Lower	Upper
Step 1[a]	rs2259816D	.250	.133	3.559	1	.059	1.284	.990	1.665
	Sex(1)	-.101	.114	.791	1	.374	.904	.723	1.130
	MI(1)	.371	.123	9.116	1	.003	1.449	1.139	1.843
	HTN(1)	.137	.133	1.070	1	.301	1.147	.885	1.487
	DM	-.186	.113	2.689	1	.101	.831	.665	1.037
	OBS(1)	-.095	.163	.336	1	.562	.910	.660	1.253
	FH(1)	-.235	.125	3.520	1	.061	.790	.618	1.011
	hChol(1)	-2.471	.126	383.648	1	.000	.085	.066	.108
	hTG(1)	-.654	.107	37.191	1	.000	.520	.422	.642
	lHDL(1)	.243	.107	5.184	1	.023	1.275	1.034	1.571
	Age	-.002	.004	.186	1	.666	.998	.990	1.006
	BMI	-.031	.015	4.411	1	.036	.970	.942	.998
	Constant	.379	.641	.350	1	.554	1.461		

a. Variable(s) entered on step 1: rs2259816D, Sex, MI, HTN, DM, OBS, FH, hChol, hTG, lHDL, Age, BMI.

Variables in the Equation

		B	S.E.	Wald	df	Sig.	Exp(B)	95% C.I.for EXP(B)	
								Lower	Upper
Step 1[a]	rs1169310A	-.175	.071	6.134	1	.013	.840	.731	.964
	MI(1)	.393	.084	21.802	1	.000	1.482	1.256	1.748
	Age	-.002	.003	.404	1	.525	.998	.993	1.004
	BMI	-.028	.010	7.866	1	.005	.973	.954	.992
	Sex(1)	-.091	.078	1.355	1	.244	.913	.784	1.064
	HTN(1)	.132	.092	2.071	1	.150	1.142	.953	1.367
	DM(1)	.183	.077	5.612	1	.018	1.201	1.032	1.397
	OBS(1)	-.051	.112	.209	1	.648	.950	.763	1.184
	hChol(1)	-2.464	.086	815.634	1	.000	.085	.072	.101
	hTG	.692	.074	88.708	1	.000	1.999	1.730	2.308
	lHDL(1)	.282	.073	14.875	1	.000	1.326	1.149	1.531
	Constant	-.086	.413	.044	1	.834	.917		

a. Variable(s) entered on step 1: rs1169310A, MI, Age, BMI, Sex, HTN, DM, OBS, hChol, hTG, lHDL.

Variables in the Equation

		B	S.E.	Wald	df	Sig.	Exp(B)	95% C.I.for EXP(B)	
								Lower	Upper
Step 1[a]	rs1169310R	-.233	.131	3.193	1	.074	.792	.613	1.023
	Sex(1)	-.102	.111	.850	1	.357	.903	.727	1.122
	MI(1)	.377	.119	9.965	1	.002	1.457	1.153	1.841
	HTN(1)	.130	.130	.997	1	.318	1.139	.883	1.469
	DM	-.157	.111	2.005	1	.157	.855	.689	1.062
	OBS(1)	-.052	.159	.109	1	.742	.949	.695	1.295
	FH(1)	-.197	.123	2.566	1	.109	.821	.645	1.045
	hChol(1)	-2.456	.122	405.429	1	.000	.086	.068	.109
	hTG(1)	-.686	.104	43.367	1	.000	.504	.411	.618
	lHDL(1)	.277	.104	7.156	1	.007	1.319	1.077	1.617
	Age	-.001	.004	.055	1	.814	.999	.991	1.007
	BMI	-.030	.014	4.395	1	.036	.971	.944	.998
	Constant	.972	.591	2.699	1	.100	2.642		

a. Variable(s) entered on step 1: rs1169310R, Sex, MI, HTN, DM, OBS, FH, hChol, hTG, lHDL, Age, BMI.

Variables in the Equation

		B	S.E.	Wald	df	Sig.	Exp(B)	95% C.I.for EXP(B)	
								Lower	Upper
Step 1[a]	rs1169310D	-.217	.100	4.704	1	.030	.805	.661	.979
	Sex(1)	-.104	.111	.892	1	.345	.901	.725	1.119
	MI(1)	.383	.119	10.305	1	.001	1.467	1.161	1.854
	HTN(1)	.123	.130	.892	1	.345	1.131	.876	1.459
	DM	-.158	.111	2.039	1	.153	.854	.687	1.061
	OBS(1)	-.049	.159	.095	1	.758	.952	.698	1.300
	FH(1)	-.196	.123	2.527	1	.112	.822	.646	1.047
	hChol(1)	-2.451	.122	403.545	1	.000	.086	.068	.110
	hTG(1)	-.685	.104	43.247	1	.000	.504	.411	.618
	lHDL(1)	.273	.104	6.964	1	.008	1.314	1.073	1.610
	Age	-.001	.004	.045	1	.831	.999	.991	1.007
	BMI	-.029	.014	4.326	1	.038	.971	.944	.998
	Constant	1.039	.593	3.072	1	.080	2.826		

a. Variable(s) entered on step 1: rs1169310D, Sex, MI, HTN, DM, OBS, FH, hChol, hTG, lHDL, Age, BMI.

Variables in the Equation

		B	S.E.	Wald	df	Sig.	Exp(B)	95% C.I.for EXP(B)	
								Lower	Upper
Step 1[a]	rs1169313A	-.160	.071	5.101	1	.024	.852	.742	.979
	MI(1)	.378	.085	20.002	1	.000	1.460	1.237	1.723
	Age	-.002	.003	.479	1	.489	.998	.992	1.004
	BMI	-.027	.010	7.139	1	.008	.974	.955	.993
	Sex(1)	-.112	.078	2.054	1	.152	.894	.768	1.042
	HTN(1)	.149	.092	2.602	1	.107	1.161	.968	1.392
	DM(1)	.148	.078	3.650	1	.056	1.160	.996	1.351
	OBS(1)	-.049	.113	.187	1	.666	.952	.764	1.188
	hChol(1)	-2.449	.087	801.117	1	.000	.086	.073	.102
	hTG	.664	.074	80.923	1	.000	1.942	1.681	2.245
	lHDL(1)	.281	.073	14.733	1	.000	1.325	1.148	1.530
	Constant	-.084	.416	.041	1	.839	.919		

a. Variable(s) entered on step 1: rs1169313A, MI, Age, BMI, Sex, HTN, DM, OBS, hChol, hTG, lHDL.

Variables in the Equation

		B	S.E.	Wald	df	Sig.	Exp(B)	95% C.I.for EXP(B)	
								Lower	Upper
Step 1[a]	rs1169313R	-.186	.130	2.044	1	.153	.830	.644	1.071
	Sex(1)	-.123	.111	1.246	1	.264	.884	.712	1.098
	MI(1)	.361	.120	9.099	1	.003	1.435	1.135	1.815
	HTN(1)	.145	.131	1.240	1	.265	1.157	.895	1.494
	DM	-.123	.111	1.223	1	.269	.884	.711	1.100
	OBS(1)	-.049	.159	.094	1	.759	.952	.697	1.301
	FH(1)	-.190	.124	2.372	1	.124	.827	.649	1.053
	hChol(1)	-2.440	.122	398.274	1	.000	.087	.069	.111
	hTG(1)	-.656	.105	39.400	1	.000	.519	.423	.637
	lHDL(1)	.276	.104	7.085	1	.008	1.318	1.076	1.616
	Age	-.001	.004	.084	1	.772	.999	.991	1.007
	BMI	-.028	.014	3.969	1	.046	.972	.945	1.000
	Constant	.869	.595	2.138	1	.144	2.385		

a. Variable(s) entered on step 1: rs1169313R, Sex, MI, HTN, DM, OBS, FH, hChol, hTG, lHDL, Age, BMI.

Variables in the Equation

		B	S.E.	Wald	df	Sig.	Exp(B)	95% C.I.for EXP(B)	
								Lower	Upper
Step 1[a]	rs1169313D	-.214	.101	4.512	1	.034	.807	.663	.984
	Sex(1)	-.125	.111	1.270	1	.260	.883	.711	1.096
	MI(1)	.371	.120	9.567	1	.002	1.449	1.145	1.833
	HTN(1)	.141	.131	1.161	1	.281	1.151	.891	1.488
	DM	-.124	.111	1.247	1	.264	.883	.710	1.098
	OBS(1)	-.047	.160	.085	1	.770	.954	.698	1.305
	FH(1)	-.188	.124	2.307	1	.129	.829	.650	1.056
	hChol(1)	-2.437	.122	396.592	1	.000	.087	.069	.111
	hTG(1)	-.659	.105	39.641	1	.000	.517	.422	.635
	lHDL(1)	.274	.104	6.944	1	.008	1.315	1.073	1.611
	Age	-.001	.004	.072	1	.789	.999	.991	1.007
	BMI	-.028	.014	3.930	1	.047	.972	.945	1.000
	Constant	.989	.597	2.747	1	.097	2.688		

a. Variable(s) entered on step 1: rs1169313D, Sex, MI, HTN, DM, OBS, FH, hChol, hTG, lHDL, Age, BMI.

HNF1a vs Family History

rs2393791 vs

Variables in the Equation

		B	S.E.	Wald	df	Sig.	Exp(B)	95% C.I.for EXP(B)	
								Lower	Upper
Step 1[a]	rs2393791R(1)	-.041	.108	.143	1	.705	.960	.777	1.186
	Sex(1)	.290	.093	9.670	1	.002	1.336	1.113	1.604
	MI(1)	.258	.098	6.982	1	.008	1.294	1.069	1.568
	HTN(1)	.035	.099	.123	1	.726	1.035	.852	1.258
	DM	-.833	.093	80.398	1	.000	.435	.362	.521
	OBS(1)	-.020	.129	.024	1	.877	.980	.760	1.263
	hChol(1)	-.191	.099	3.743	1	.053	.826	.681	1.002
	hTG(1)	-.240	.097	6.068	1	.014	.787	.650	.952
	lHDL(1)	.094	.087	1.160	1	.282	1.098	.926	1.303
	hLDL(1)	-.229	.121	3.598	1	.058	.795	.627	1.008
	Age	-.023	.003	50.365	1	.000	.977	.971	.983
	BMI	.043	.010	17.029	1	.000	1.044	1.023	1.065
	Constant	-.848	.445	3.629	1	.057	.428		

a. Variable(s) entered on step 1: rs2393791R, Sex, MI, HTN, DM, OBS, hChol, hTG, lHDL, hLDL, Age, BMI.

Variables in the Equation

		B	S.E.	Wald	df	Sig.	Exp(B)	95% C.I.for EXP(B)	
								Lower	Upper
Step 1[a]	rs2393791D	-.011	.084	.016	1	.901	.990	.839	1.168
	Sex(1)	.290	.093	9.713	1	.002	1.337	1.114	1.605
	MI(1)	.259	.098	7.032	1	.008	1.296	1.070	1.569
	HTN(1)	.033	.099	.111	1	.739	1.034	.851	1.256
	DM	-.834	.093	80.481	1	.000	.434	.362	.521
	OBS(1)	-.019	.129	.023	1	.881	.981	.761	1.264
	hLDL(1)	-.229	.121	3.572	1	.059	.796	.628	1.009
	hChol(1)	-.191	.099	3.735	1	.053	.826	.681	1.003
	hTG(1)	-.241	.097	6.131	1	.013	.786	.649	.951
	lHDL(1)	.094	.087	1.167	1	.280	1.099	.926	1.303
	Age	-.023	.003	50.562	1	.000	.977	.971	.983
	BMI	.043	.010	17.108	1	.000	1.044	1.023	1.066
	Constant	-.866	.456	3.601	1	.058	.421		

a. Variable(s) entered on step 1: rs2393791D, Sex, MI, HTN, DM, OBS, hLDL, hChol, hTG, lHDL, Age, BMI.

Variables in the Equation

		B	S.E.	Wald	df	Sig.	Exp(B)	95% C.I.for EXP(B)	
								Lower	Upper
Step 1[a]	rs7310409R	.069	.108	.409	1	.523	1.072	.867	1.325
	Sex(1)	.275	.093	8.751	1	.003	1.316	1.097	1.579
	MI(1)	.251	.097	6.650	1	.010	1.286	1.062	1.557
	HTN(1)	.038	.099	.146	1	.702	1.039	.855	1.261
	DM	-.843	.093	82.659	1	.000	.430	.359	.516
	OBS(1)	-.027	.129	.043	1	.836	.974	.756	1.254
	hLDL(1)	-.215	.121	3.165	1	.075	.807	.637	1.022
	hChol(1)	-.198	.099	4.016	1	.045	.821	.676	.996
	hTG(1)	-.241	.097	6.132	1	.013	.786	.650	.951
	lHDL(1)	.088	.087	1.036	1	.309	1.092	.921	1.295
	Age	-.023	.003	51.410	1	.000	.977	.971	.983
	BMI	.042	.010	15.993	1	.000	1.043	1.021	1.064
	Constant	-.900	.453	3.950	1	.047	.407		

a. Variable(s) entered on step 1: rs7310409R, Sex, MI, HTN, DM, OBS, hLDL, hChol, hTG, lHDL, Age, BMI.

Variables in the Equation

		B	S.E.	Wald	df	Sig.	Exp(B)	95% C.I.for EXP(B)	
								Lower	Upper
Step 1[a]	rs7310409D	-.014	.084	.029	1	.865	.986	.836	1.163
	Sex(1)	.276	.093	8.820	1	.003	1.318	1.098	1.581
	MI(1)	.253	.097	6.724	1	.010	1.288	1.064	1.559
	HTN(1)	.035	.099	.122	1	.726	1.035	.852	1.257
	DM	-.844	.093	82.735	1	.000	.430	.358	.516
	OBS(1)	-.026	.129	.042	1	.838	.974	.756	1.254
	hLDL(1)	-.214	.121	3.131	1	.077	.808	.637	1.023
	hChol(1)	-.197	.099	3.993	1	.046	.821	.677	.996
	hTG(1)	-.243	.097	6.222	1	.013	.785	.648	.949
	lHDL(1)	.089	.087	1.053	1	.305	1.093	.922	1.296
	Age	-.023	.003	51.663	1	.000	.977	.971	.983
	BMI	.042	.010	16.062	1	.000	1.043	1.022	1.064
	Constant	-.796	.455	3.062	1	.080	.451		

a. Variable(s) entered on step 1: rs7310409D, Sex, MI, HTN, DM, OBS, hLDL, hChol, hTG, lHDL, Age, BMI.

Variables in the Equation

		B	S.E.	Wald	df	Sig.	Exp(B)	95% C.I.for EXP(B)	
								Lower	Upper
Step 1[a]	rs2259820R	.000	.090	.000	1	.996	1.000	.838	1.194
	Sex(1)	.266	.094	8.076	1	.004	1.305	1.086	1.568
	MI(1)	.252	.099	6.537	1	.011	1.287	1.061	1.562
	HTN(1)	.044	.100	.195	1	.658	1.045	.859	1.272
	DM	-.828	.094	77.883	1	.000	.437	.364	.525
	OBS(1)	-.033	.131	.062	1	.803	.968	.749	1.251
	hLDL(1)	-.212	.122	3.012	1	.083	.809	.636	1.028
	hChol(1)	-.208	.100	4.337	1	.037	.812	.668	.988
	hTG(1)	-.222	.098	5.103	1	.024	.801	.660	.971
	lHDL(1)	.073	.088	.692	1	.405	1.076	.906	1.277
	Age	-.023	.003	49.298	1	.000	.977	.971	.984
	BMI	.040	.011	14.033	1	.000	1.040	1.019	1.062
	Constant	-.765	.460	2.759	1	.097	.465		

a. Variable(s) entered on step 1: rs2259820R, Sex, MI, HTN, DM, OBS, hLDL, hChol, hTG, lHDL, Age, BMI.

Variables in the Equation

		B	S.E.	Wald	df	Sig.	Exp(B)	95% C.I.for EXP(B)	
								Lower	Upper
Step 1[a]	rs2259820D	-.162	.095	2.933	1	.087	.850	.706	1.024
	Sex(1)	.266	.094	8.062	1	.005	1.305	1.086	1.568
	MI(1)	.252	.099	6.530	1	.011	1.287	1.061	1.561
	HTN(1)	.050	.100	.245	1	.621	1.051	.864	1.278
	DM	-.821	.094	76.710	1	.000	.440	.366	.529
	OBS(1)	-.034	.131	.066	1	.797	.967	.748	1.250
	hLDL(1)	-.217	.122	3.154	1	.076	.805	.633	1.023
	hChol(1)	-.208	.100	4.336	1	.037	.812	.668	.988
	hTG(1)	-.218	.098	4.927	1	.026	.804	.663	.975
	lHDL(1)	.074	.088	.707	1	.400	1.077	.907	1.278
	Age	-.023	.003	49.100	1	.000	.977	.971	.984
	BMI	.039	.011	13.839	1	.000	1.040	1.019	1.062
	Constant	-.477	.474	1.016	1	.313	.620		

a. Variable(s) entered on step 1: rs2259820D, Sex, MI, HTN, DM, OBS, hLDL, hChol, hTG, lHDL, Age, BMI.

Variables in the Equation

		B	S.E.	Wald	df	Sig.	Exp(B)	95% C.I.for EXP(B)	
								Lower	Upper
Step 1[a]	rs2464196R	.151	.094	2.558	1	.110	1.163	.967	1.399
	Sex(1)	.295	.093	9.979	1	.002	1.343	1.119	1.613
	MI(1)	.263	.098	7.186	1	.007	1.301	1.073	1.576
	HTN(1)	.040	.099	.161	1	.688	1.041	.856	1.265
	DM	-.837	.093	80.364	1	.000	.433	.361	.520
	OBS(1)	-.006	.130	.002	1	.965	.994	.771	1.282
	hLDL(1)	-.215	.122	3.108	1	.078	.807	.635	1.024
	hChol(1)	-.193	.099	3.790	1	.052	.824	.678	1.001
	hTG(1)	-.257	.098	6.936	1	.008	.773	.638	.936
	lHDL(1)	.094	.087	1.151	1	.283	1.098	.925	1.303
	Age	-.023	.003	50.881	1	.000	.977	.971	.983
	BMI	.043	.010	16.720	1	.000	1.044	1.022	1.065
	Constant	-1.069	.452	5.588	1	.018	.343		

a. Variable(s) entered on step 1: rs2464196R, Sex, MI, HTN, DM, OBS, hLDL, hChol, hTG, lHDL, Age, BMI.

Variables in the Equation

		B	S.E.	Wald	df	Sig.	Exp(B)	95% C.I.for EXP(B)	
								Lower	Upper
Step 1[a]	rs2464196D	-.002	.090	.000	1	.985	.998	.837	1.191
	Sex(1)	.295	.093	9.982	1	.002	1.343	1.118	1.613
	MI(1)	.263	.098	7.213	1	.007	1.301	1.074	1.577
	HTN(1)	.036	.099	.131	1	.718	1.037	.853	1.260
	DM	-.842	.093	81.313	1	.000	.431	.359	.517
	OBS(1)	-.005	.130	.001	1	.972	.995	.772	1.284
	hLDL(1)	-.210	.122	2.968	1	.085	.811	.639	1.029
	hChol(1)	-.193	.099	3.793	1	.051	.824	.678	1.001
	hTG(1)	-.260	.098	7.107	1	.008	.771	.636	.933
	lHDL(1)	.092	.087	1.119	1	.290	1.097	.924	1.301
	Age	-.023	.003	50.992	1	.000	.977	.971	.983
	BMI	.043	.010	16.944	1	.000	1.044	1.023	1.065
	Constant	-.884	.460	3.685	1	.055	.413		

a. Variable(s) entered on step 1: rs2464196D, Sex, MI, HTN, DM, OBS, hLDL, hChol, hTG, lHDL, Age, BMI.

Variables in the Equation

		B	S.E.	Wald	df	Sig.	Exp(B)	95% C.I.for EXP(B)	
								Lower	Upper
Step 1[a]	rs2259816R	-.087	.089	.952	1	.329	.917	.771	1.091
	Sex(1)	.304	.096	10.093	1	.001	1.355	1.124	1.635
	MI(1)	.276	.100	7.646	1	.006	1.318	1.084	1.604
	HTN(1)	.039	.102	.150	1	.699	1.040	.852	1.269
	DM	-.817	.095	74.180	1	.000	.442	.367	.532
	OBS(1)	.030	.133	.053	1	.819	1.031	.795	1.337
	hLDL(1)	-.263	.124	4.504	1	.034	.769	.603	.980
	hChol(1)	-.200	.101	3.888	1	.049	.819	.672	.999
	hTG(1)	-.208	.100	4.338	1	.037	.812	.668	.988
	lHDL(1)	.096	.089	1.156	1	.282	1.100	.924	1.310
	Age	-.023	.003	49.672	1	.000	.977	.970	.983
	BMI	.047	.011	19.559	1	.000	1.048	1.027	1.070
	Constant	-.909	.465	3.824	1	.051	.403		

a. Variable(s) entered on step 1: rs2259816R, Sex, MI, HTN, DM, OBS, hLDL, hChol, hTG, lHDL, Age, BMI.

Variables in the Equation

		B	S.E.	Wald	df	Sig.	Exp(B)	95% C.I.for EXP(B)	
								Lower	Upper
Step 1[a]	rs2259816D	-.062	.104	.351	1	.554	.940	.766	1.154
	Sex(1)	.302	.096	9.986	1	.002	1.353	1.122	1.632
	MI(1)	.278	.100	7.735	1	.005	1.320	1.086	1.606
	HTN(1)	.038	.102	.140	1	.708	1.039	.851	1.268
	DM	-.818	.095	74.448	1	.000	.441	.366	.531
	OBS(1)	.034	.133	.064	1	.800	1.034	.797	1.341
	hLDL(1)	-.260	.124	4.423	1	.035	.771	.605	.982
	hChol(1)	-.198	.101	3.838	1	.050	.820	.672	1.000
	hTG(1)	-.211	.100	4.449	1	.035	.810	.666	.985
	lHDL(1)	.095	.089	1.142	1	.285	1.100	.924	1.309
	Age	-.023	.003	49.472	1	.000	.977	.970	.983
	BMI	.047	.011	19.666	1	.000	1.048	1.027	1.071
	Constant	-.921	.486	3.586	1	.058	.398		

a. Variable(s) entered on step 1: rs2259816D, Sex, MI, HTN, DM, OBS, hLDL, hChol, hTG, lHDL, Age, BMI.

Variables in the Equation

		B	S.E.	Wald	df	Sig.	Exp(B)	95% C.I.for EXP(B)	
								Lower	Upper
Step 1[a]	rs1169310R	.076	.103	.543	1	.461	1.079	.881	1.322
	Sex(1)	.288	.093	9.549	1	.002	1.334	1.111	1.602
	MI(1)	.244	.098	6.200	1	.013	1.276	1.053	1.545
	HTN(1)	.024	.099	.057	1	.811	1.024	.843	1.244
	DM	-.850	.093	83.539	1	.000	.427	.356	.513
	OBS(1)	-.023	.129	.033	1	.857	.977	.758	1.259
	hLDL(1)	-.222	.121	3.341	1	.068	.801	.632	1.016
	hChol(1)	-.200	.099	4.097	1	.043	.818	.674	.994
	hTG(1)	-.248	.097	6.506	1	.011	.780	.645	.944
	lHDL(1)	.084	.087	.939	1	.332	1.088	.917	1.290
	Age	-.024	.003	52.787	1	.000	.977	.970	.983
	BMI	.042	.010	16.146	1	.000	1.043	1.022	1.064
	Constant	-.888	.451	3.868	1	.049	.412		

a. Variable(s) entered on step 1: rs1169310R, Sex, MI, HTN, DM, OBS, hLDL, hChol, hTG, lHDL, Age, BMI.

Variables in the Equation

		B	S.E.	Wald	df	Sig.	Exp(B)	95% C.I.for EXP(B)	
								Lower	Upper
Step 1[a]	rs1169310D	.030	.086	.121	1	.728	1.030	.870	1.220
	Sex(1)	.289	.093	9.619	1	.002	1.336	1.112	1.603
	MI(1)	.244	.098	6.217	1	.013	1.276	1.054	1.546
	HTN(1)	.023	.099	.054	1	.816	1.023	.842	1.243
	DM	-.851	.093	83.691	1	.000	.427	.356	.512
	OBS(1)	-.025	.129	.036	1	.849	.976	.757	1.257
	hLDL(1)	-.222	.121	3.338	1	.068	.801	.632	1.016
	hChol(1)	-.201	.099	4.112	1	.043	.818	.674	.993
	hTG(1)	-.249	.097	6.530	1	.011	.780	.644	.944
	lHDL(1)	.084	.087	.939	1	.333	1.088	.917	1.290
	Age	-.024	.003	52.825	1	.000	.977	.970	.983
	BMI	.042	.010	16.180	1	.000	1.043	1.022	1.064
	Constant	-.846	.455	3.465	1	.063	.429		

a. Variable(s) entered on step 1: rs1169310D, Sex, MI, HTN, DM, OBS, hLDL, hChol, hTG, lHDL, Age, BMI.

Variables in the Equation

		B	S.E.	Wald	df	Sig.	Exp(B)	95% C.I.for EXP(B)	
								Lower	Upper
Step 1[a]	rs1169313R	.059	.105	.322	1	.571	1.061	.864	1.303
	Sex(1)	.283	.094	9.092	1	.003	1.327	1.104	1.595
	MI(1)	.229	.099	5.367	1	.021	1.257	1.036	1.526
	HTN(1)	.019	.100	.037	1	.847	1.020	.837	1.241
	DM	-.836	.094	79.439	1	.000	.434	.361	.521
	OBS(1)	-.024	.130	.033	1	.855	.976	.756	1.261
	hLDL(1)	-.216	.122	3.133	1	.077	.806	.635	1.023
	hChol(1)	-.193	.100	3.728	1	.054	.825	.678	1.003
	hTG(1)	-.231	.098	5.504	1	.019	.794	.655	.963
	lHDL(1)	.075	.088	.723	1	.395	1.077	.907	1.279
	Age	-.023	.003	49.965	1	.000	.977	.971	.983
	BMI	.041	.011	15.123	1	.000	1.042	1.020	1.063
	Constant	-.876	.457	3.673	1	.055	.416		

a. Variable(s) entered on step 1: rs1169313R, Sex, MI, HTN, DM, OBS, hLDL, hChol, hTG, lHDL, Age, BMI.

Variables in the Equation

		B	S.E.	Wald	df	Sig.	Exp(B)	95% C.I.for EXP(B)	
								Lower	Upper
Step 1[a]	rs1169313D	.004	.086	.002	1	.966	1.004	.847	1.189
	Sex(1)	.283	.094	9.119	1	.003	1.327	1.105	1.595
	MI(1)	.229	.099	5.391	1	.020	1.258	1.036	1.527
	HTN(1)	.018	.100	.031	1	.860	1.018	.836	1.239
	DM	-.837	.094	79.686	1	.000	.433	.360	.520
	OBS(1)	-.025	.130	.036	1	.850	.976	.756	1.260
	hLDL(1)	-.215	.122	3.106	1	.078	.807	.635	1.024
	hChol(1)	-.193	.100	3.737	1	.053	.824	.678	1.003
	hTG(1)	-.232	.098	5.549	1	.018	.793	.654	.962
	lHDL(1)	.075	.088	.723	1	.395	1.077	.907	1.279
	Age	-.023	.003	50.011	1	.000	.977	.971	.983
	BMI	.041	.011	15.160	1	.000	1.042	1.021	1.064
	Constant	-.811	.459	3.120	1	.077	.444		

a. Variable(s) entered on step 1: rs1169313D, Sex, MI, HTN, DM, OBS, hLDL, hChol, hTG, lHDL, Age, BMI.

HNF1a Suppl data 5

HNF1a haplotyping for the various disease traits. Pooled is the frequency of the haplotype in the whole study population; The studied SNPs are (1) rs2393791, (2) rs7310409, (3) rs2259820, (4) rs2464196, (5) rs2259816, (6) rs1169310 and (7) rs1169313 arranged sequentially by their chromosomal positions, and blocks represent the range of variants constituting the respective haplotype.

Block	Haplotype	Pooled	Cases	Controls	χ^2	P-value
**Myocardial infarction**						
1-7	CATATAC	0.505	0.516	0.486	7.502	0.0062
	TGCGGGT	0.356	0.346	0.375	7.423	0.0064
	CATAGAC	0.011	0.009	0.014	6.055	0.0139
1-6	CATATA	0.504	0.514	0.484	7.48	0.0062
	TGCGGG	0.357	0.349	0.374	5.809	0.0159
	CATAGA	0.011	0.009	0.015	7.659	0.0056
2-7	ATATAC	0.505	0.515	0.486	6.902	0.0086
	GCGGGT	0.357	0.347	0.374	6.471	0.011
	ATAGAC	0.011	0.009	0.014	6.097	0.0135
1-5	CATAT	0.503	0.513	0.483	7.683	0.0056
	TGCGG	0.361	0.353	0.377	5.217	0.0224
	CATAG	0.012	0.009	0.016	7.282	0.007
3-7	TATAC	0.514	0.525	0.494	7.966	0.0048
	CGGGT	0.396	0.387	0.412	5.329	0.021
	TAGAC	0.011	0.009	0.015	6.643	0.01
2-6	ATATA	0.504	0.514	0.485	7.015	0.0081
	GCGGG	0.359	0.351	0.374	4.936	0.0263
	ATAGA	0.011	0.009	0.015	7.643	0.0057
2-5	ATAT	0.503	0.513	0.484	6.699	0.0096
	GCGG	0.363	0.355	0.378	4.643	0.0312
	ATAG	0.012	0.010	0.016	5.223	0.0223
3-6	TATA	0.514	0.525	0.493	8.363	0.0038
	CGGG	0.398	0.390	0.413	4.32	0.0377
	TAGA	0.012	0.010	0.016	5.817	0.0159
4-7	ATAC	0.515	0.525	0.496	7.258	0.0071
	GGGT	0.395	0.387	0.412	5.722	0.0168
	AGAC	0.012	0.010	0.016	6.865	0.0088
1-4	CATA	0.513	0.521	0.498	4.364	0.0367
	TGCG	0.369	0.359	0.387	7.31	0.0069

Block	Haplotype	Pooled	Cases	Controls	$\chi 2$	P-value
**Type 2 diabetes mellitus**						
1-7	CATATAC	0.505	0.515	0.494	3.944	0.047
1-6	CATATA	0.504	0.513	0.493	3.588	0.0582
2-7	ATATAC	0.505	0.514	0.493	3.731	0.0534
	ACGGGT	0.04	0.037	0.045	3.68	0.0551
1-5	CATAT	0.503	0.512	0.492	3.824	0.0505
2-6	ATATA	0.504	0.513	0.494	3.477	0.0622
	ACGGG	0.04	0.037	0.045	3.659	0.0558
2-5	ATAT	0.503	0.512	0.492	3.629	0.0568
	ACGG	0.041	0.038	0.045	3.288	0.0698
1-4	CATA	0.513	0.523	0.502	3.964	0.0465
**Obesity**						
1-7	CACGTAC	0.047	0.042	0.053	5.159	0.0231
1-6	CACGTA	0.047	0.042	0.053	5.827	0.0158
	CACGGG	0.035	0.039	0.032	3.496	0.0615
2-7	ACGTAC	0.047	0.041	0.053	6.168	0.013
	ACGGGT	0.04	0.046	0.036	5.042	0.0247
1-5	CACGT	0.047	0.042	0.053	5.012	0.0252
2-6	ACGTA	0.047	0.041	0.053	6.394	0.0114
	ACGGG	0.04	0.047	0.036	5.842	0.0156
	ACGT	0.047	0.042	0.052	4.835	0.0279
2-5	ACGG	0.041	0.046	0.038	3.261	0.071

Block	Haplotype	Pooled	Cases	Controls	χ^2	P-value
**Hypercholesterolemia**						
1-7	TGCGGGT	0.356	0.342	0.363	3.934	0.0473
1-6	TGCGGG	0.357	0.345	0.363	3.191	0.074
2-7	GCGGGT	0.357	0.342	0.363	3.927	0.0475
1-4	TGCG	0.369	0.356	0.375	3.306	0.069
**Hypertriglyceridemia**						
1-7	TGCGGGT	0.356	0.373	0.351	3.703	0.0543
1-6	TGCGGG	0.357	0.376	0.352	4.261	0.039
2-7	GCGGGT	0.357	0.373	0.351	3.606	0.0576
1-5	TGCGG	0.361	0.380	0.355	4.611	0.0318
3-7	CGGGT	0.396	0.415	0.390	4.233	0.0397
2-6	GCGGG	0.359	0.377	0.353	3.902	0.0482
1-4	TGCG	0.369	0.386	0.364	3.411	0.0648
4-7	GGGT	0.395	0.414	0.390	4.274	0.0387
2-5	GCGG	0.363	0.380	0.357	3.783	0.0518
**Low High Density Lipoprotein-Cholesterol**						
1-7	CATAGAC	0.011	0.009	0.013	3.811	0.0509
	TGTATAC	0.011	0.008	0.013	5.189	0.0227
1-6	CATAGA	0.011	0.009	0.014	5.166	0.023
	TGTATA	0.01	0.008	0.013	5.061	0.0245
2-7	GTATAC	0.012	0.009	0.014	6.333	0.0119
	ATAGAC	0.011	0.009	0.013	3.783	0.0518
1-5	TGTAT	0.013	0.010	0.015	3.698	0.0545
	CATAG	0.012	0.009	0.014	4.463	0.0346
3-7	TAGAC	0.011	0.009	0.014	4.601	0.032
2-6	GTATA	0.012	0.009	0.014	5.737	0.0166
	ATAGA	0.011	0.009	0.014	5.159	0.0231
4-7	AGAC	0.012	0.010	0.014	3.964	0.0465
2-5	GTAT	0.013	0.010	0.016	4.94	0.0262
	ATAG	0.012	0.010	0.014	3.426	0.0642
3-6	TAGA	0.012	0.010	0.014	3.817	0.0507
2-4	GTA	0.014	0.011	0.017	4.633	0.0314
